# Metagenomics of the Svalbard Reindeer Rumen Microbiome Reveals Abundance of Polysaccharide Utilization Loci

**DOI:** 10.1371/journal.pone.0038571

**Published:** 2012-06-06

**Authors:** Phillip B. Pope, Alasdair K. Mackenzie, Ivan Gregor, Wendy Smith, Monica A. Sundset, Alice C. McHardy, Mark Morrison, Vincent G.H. Eijsink

**Affiliations:** 1 Department of Chemistry, Biotechnology and Food Science, Norwegian University of Life Sciences, Ås, Norway; 2 Max-Planck Research Group for Computational Genomics and Epidemiology, Max-Planck Institute for Informatics, Saarbrücken, Germany; 3 Department for Algorithmic Bioinformatics, Heinrich-Heine University Düsseldorf, Düsseldorf, Germany; 4 CSIRO Livestock Industries, Queensland Bioscience Precinct, St Lucia, Australia; 5 Department of Arctic and Marine Biology, University of Tromsø, Tromsø, Norway; Auburn University, United States of America

## Abstract

Lignocellulosic biomass remains a largely untapped source of renewable energy predominantly due to its recalcitrance and an incomplete understanding of how this is overcome in nature. We present here a compositional and comparative analysis of metagenomic data pertaining to a natural biomass-converting ecosystem adapted to austere arctic nutritional conditions, namely the rumen microbiome of Svalbard reindeer (*Rangifer tarandus platyrhynchus*). Community analysis showed that deeply-branched cellulolytic lineages affiliated to the Bacteroidetes and Firmicutes are dominant, whilst sequence binning methods facilitated the assemblage of metagenomic sequence for a dominant and novel Bacteroidales clade (SRM-1). Analysis of unassembled metagenomic sequence as well as metabolic reconstruction of SRM-1 revealed the presence of multiple polysaccharide utilization loci-like systems (PULs) as well as members of more than 20 glycoside hydrolase and other carbohydrate-active enzyme families targeting various polysaccharides including cellulose, xylan and pectin. Functional screening of cloned metagenome fragments revealed high cellulolytic activity and an abundance of PULs that are rich in endoglucanases (GH5) but devoid of other common enzymes thought to be involved in cellulose degradation. Combining these results with known and partly re-evaluated metagenomic data strongly indicates that much like the human distal gut, the digestive system of herbivores harbours high numbers of deeply branched and as-yet uncultured members of the Bacteroidetes that depend on PUL-like systems for plant biomass degradation.

## Introduction

Understanding the enzymology of plant biomass conversion is a key issue in the world’s desire to establish a sustainable bio-based economy. Whilst current available enzyme technology is insufficiently effective, many free-living organisms readily deconstruct plant biomass by enzyme-driven hydrolysis to take advantage of this material as a nutrient source. In particular, obligate herbivores have evolved to maintain a symbiotic relationship with a specialized consortium of gut microbes (microbiomes) that underpins lignocellulose deconstruction. Current paradigms for microbial lignocellulose degradation in gut microbiomes are centered on well known key cellulolytic enzymes (i.e. GH5, GH6, GH7, GH9 and GH48; see [1] for enzyme classification) and multi-enzyme cellulosome complexes [2].

Accumulating knowledge indicates that these paradigms are not unique and that nature has additional, as yet poorly understood tools to accomplish lignocellulose degradation. For instance, the gut bacterium *Fibrobacter succinogenes* has long been known to degrade crystalline cellulose and other plant structural polysaccharides at a rate exceeding that of most other microorganisms. However genome studies indicate this bacterium lacks both GH6, processive GH9, and GH48 representatives and cellulosome structures [3]. The absence or poor representation of key enzymes and cellulosomes in cellulolytic gut microbiome communities has been further exemplified by recent metagenome sequencing projects in the hindgut of termites [4], the foregut of marsupials [5] and the rumen of cows [6], [7]. Here we have explored this further by studying the rumen microbiome of the Svalbard reindeer, a herbivore whose extreme habitat and diet are very different compared to the herbivores studied so far.

Svalbard reindeer (*Rangifer tarandus platyrhynchus*) live under austere nutritional conditions on the high-arctic archipelago of Svalbard (74–80°N lat.), where snow and ice cover most vegetation for more than eight months of the year. In winter time, the reindeer feed on poor quality forages that are high in lignocellulose. Body reserves are not sufficient for winter survival, and it has been estimated that only 10–30% of the daily energy expenditure during the dark part of winter can be covered by mobilization of fat [8]. Therefore, optimal utilization of the feed during winter time is crucial for survival and metabolic capabilities of the rumen microbiome are likely to play a central role. Indeed, studies on cultivable members of the microbiome showed high levels of bacteria capable of degrading various forms of cellulose and heteroxylans [8], whilst a limited cultivation-independent study suggested that the rumen microbiomes of Svalbard reindeer are dominated by novel species [9].

Svalbard reindeer research to date indicates the presence of a microbiome with high microbial diversity, powerful functional capabilities in terms of cellulose degradation, and a considerable degree of novelty. We present here a compositional and comparative analysis of metagenomic data for the rumen microbiome of Svalbard reindeer during winter. Novel bacterial lineages were identified and nucleotide composition-based sequence binning using PhyloPythiaS [10] facilitated the production of a 0.9 Mb assemblage of DNA representing one of the novel Bacteroidales clades numerically dominant in this community. Further *in silico* analysis revealed the presence of polysaccharide utilization loci-like systems (PULs) that resemble multiprotein starch utilization systems (Sus) [11] as well as the presence of carbohydrate-active enzymes targeting a broad spectrum of polysaccharides, in both this clade and the unassembled metagenome. Additional functional screens of fosmid libraries and data-mining of existing metagenome datasets revealed cellulose-degrading loci and PUL-like systems at an exceptionally high frequency in the Svalbard reindeer rumen as well as other herbivore gut environments [7], [12].

## Results and Discussion

### Metagenomic Sequence Generation

Total DNA was extracted from rumen samples collected from two Svalbard reindeer (SR1 and SR2) feeding on natural winter pasture, when the percentage of fibre degrading bacteria is believed to be at its maximum [8], [9]. A 454 pyrosequencing scheme with four technical replicate PCR reactions (each with a unique barcode) for each animal (2 animals × 4 replicates: 8 samples in total), was used to obtain ∼80,000 sequence reads from PCR-amplified V1–V3 regions of bacterial 16S rRNA genes (average read length ∼490 nt). In order to eliminate noise introduced during PCR and sequencing of 16S rRNA genes, operational taxonomic units (OTUs: defined using a 97% sequence identity threshold) were only included in the community analysis if their representatives were found in at least four of the eight samples.

To facilitate access to the metagenome and subsequent descriptions of microbial community function, approximately 503 Mb of raw single- and paired-end shotgun reads were generated from pooled rumen microbiome community DNA (2 animals). A proportion of the reads could be assembled into contigs greater than 500 nt (a total of 32,073 contigs, 26 Mb in total) with the largest contig being 17,446 nt. Paired-end sequences were used to construct 1364 scaffolds (scaffolds represent one or more contigs ordered and oriented using paired-end reads) for taxonomic binning using PhyloPythiaS (avg: 3986 nt, largest 46,178 nt; 5.44 Mb in total). Another 1.2 Mb of metagenomic DNA sequence was obtained by assembling and manual editing of the sequences of selected fosmids.

### Microbial Community Composition and the Dominance of the Bacteroidetes

**Figure 1:**
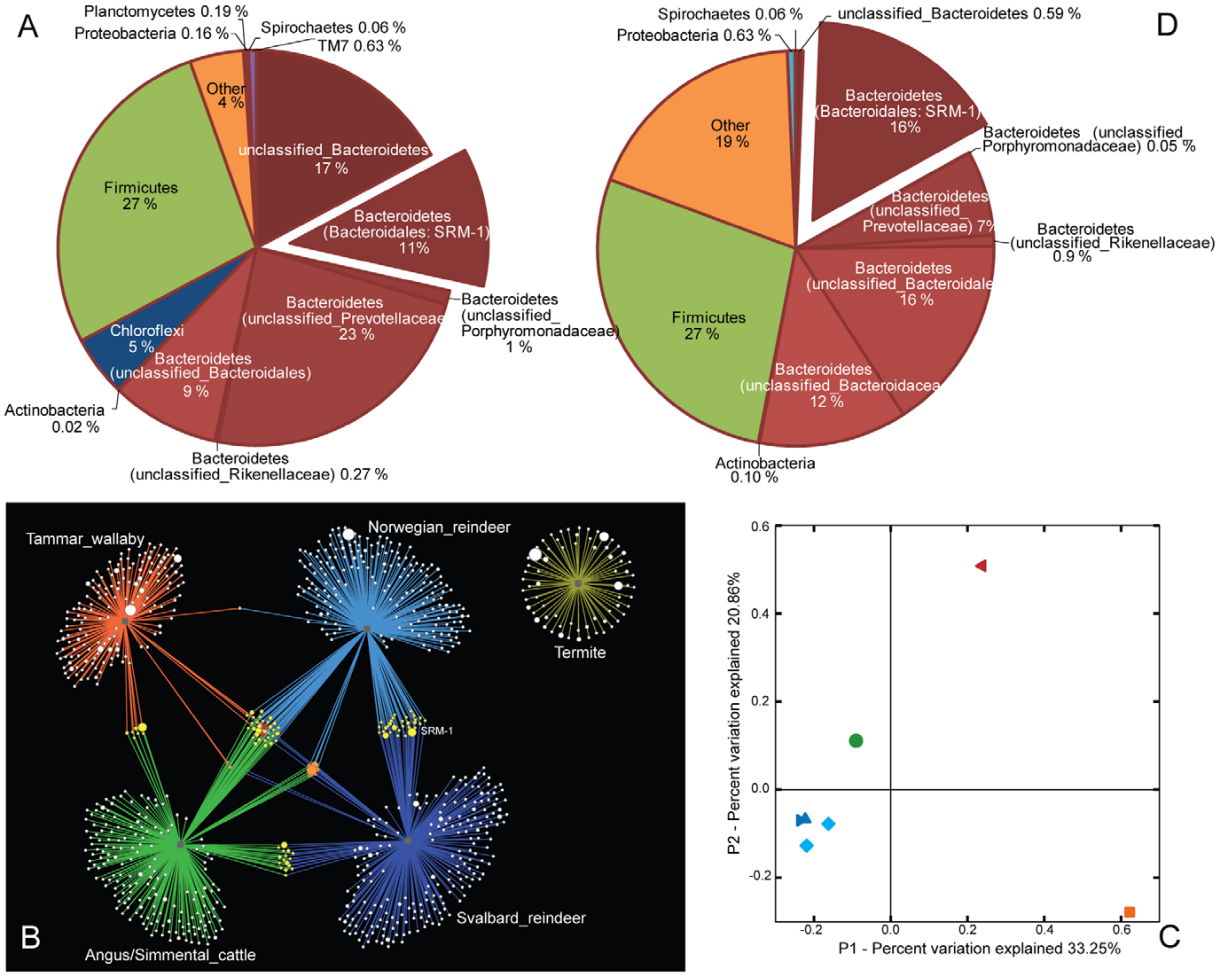
Microbial community analysis of the Svalbard reindeer rumen microbiome and comparison with microbiomes from other selected gut environments. (**A**) Diversity and relative abundance of the most abundant bacterial taxa identified in the rumen of the Svalbard reindeer based on phylogenetic analysis of 16S rRNA genes. (**B**) OTU network map showing OTU interactions between all rarefied samples from the Svalbard reindeer, Norwegian reindeer feeding on a commercial feed, Tammar wallaby, rumen and termite. OTUs are represented by dots and dot sizes reflect sequence counts within the OTU. Dot colour indicates the number of microbiomes in which the OTU was found (1 =  white, 2 =  yellow, 3 =  orange, 4 =  red). The lines radiating from each of the five grey dots link the OTUs to their source microbiomes: Svalbard reindeer, dark blue (this study); Norwegian reindeer, light blue (this study; dataset included for comparative purposes only); Termite_PL3, yellow [4]; bovine, green [6]; Tammar wallaby, orange [5]. (**C**) Principal coordinate axes (PCoA) for the unweighted UniFrac analyses are coloured by host animal; Svalbard reindeer (2 samples: ▸▴) dark-blue; Norwegian reindeer (2 samples: ♦♦) light-blue; bovine rumen (•) green; Tammar wallaby (◂) red; termite (▪) orange. (**D**) Composition of the Svalbard reindeer rumen metagenome sequence dataset, based on sequence composition-based binning of 1394 assembled scaffolds (∼5.4 Mb) using PhyloPythiaS. For a complete *rrs* inventory and comparisons between the two Svalbard reindeer samples at an OTU definition of 97% ID see Table S1.

The microbial community structure was determined using both 16S rRNA gene amplicon pyrosequencing and sequence-composition binning (PhyloPythiaS) of scaffolds assembled from metagenomic sequences (Figure 1). Using 16S rRNA gene analysis we determined there was little variation between the two animals used for this study: 90.4% and 91.5% of OTUs found in the rumen communities of animals SR1 and SR2, respectively, were shared between the two samples (Figure 1, Table S1). There were only a few instances of large differences in OTU relative abundance between the two samples (Table S1).

Comparison of OTUs against the Ribosomal Database Project [13] revealed that the Bacteroidetes and Firmicutes were largely predominant constituting 61% and 27% of the gene amplicon sequences respectively (Figure 1a). Dominance of these phyla is commonly observed in gut microbiomes. However, at an OTU-level as many as 85% of the OTUs could not be classified to a Genus-rank (Table S1), whilst the 20 most abundant OTUs demonstrated low sequence identities with cultured representatives (Figure S1). This suggests that the OTUs generally are only distantly related to any of the cultivated species from other gut environments. Furthermore, OTU-level comparisons using network maps showed a limited degree of shared OTUs between the Svalbard reindeer and other foregut-digesting herbivores, including the Norwegian reindeer (*Rangifer tarandus tarandus*) fed a commercial pelleted feed (Figure 1b). Community-level comparisons with Unifrac, which analyze phylogenetic lineages and not just shared OTUs, showed that the microbiome of the Svalbard reindeer rumen was more similar to that of the Norwegian reindeer than to those from other herbivores (Figure 1c). A small core-set of six OTUs was present in all ruminants (both reindeer species and cow), as well as two more that were present in all foregut digesters (macropods included). Interestingly these eight OTUs were all affiliated to either Ruminococcaceae or Lachnospiraceae lineages (Firmicutes) (Figure 1b, Table S1). Recent broad-scale phylogenetic analysis of the human gastrointestinal microbiome has identified highly prevalent core phylogroups belonging to the Lachnospiraceae [14], and evidence presented here suggests that such groups exist for foregut digesters as well. Most OTUs in the Svalbard reindeer rumen were present at low abundance, however several deeply branched unique members of the Bacteroidales (Bacteroidetes) were found in high abundance (Table S1). One OTU in particular, hereafter referred to as SRM-1, comprised 11% of the total 16S rRNA gene dataset. The SRM-1 OTU was found in both reindeer species only (Figure 1b) and has never before been reported. It demonstrates only 91% sequence identity to its closest cultured relative, *Bacteroidales genomosp* P1.

Taxonomic analysis of the metagenome sequences by sequence-composition binning of assembled scaffolds with PhyloPythiaS revealed a similar community structure to that observed when using 16S rRNA gene amplicon pyrosequencing analysis, with ∼75% of scaffolds assigned to the Bacteroidetes and Firmicutes (Figure 1d, Table S2). The dominance of SRM-1 was confirmed with over 880 kb (∼16%) of scaffold sequence being assigned to this OTU. Noteworthy contrasts between the two approaches included: (1) the absence of metagenomic scaffolds binned to the phylum Chloroflexi, which had several numerically abundant OTUs in the 16S rRNA gene amplicon pyrosequencing analysis (Figure 1a, Figure S1), and (2) the higher percentage of metagenomic scaffolds that were assigned as “other”. We hypothesize that due to the deep-branching nature of the Chloroflexi OTUs (the closest cultured relative has 90–91% 16S sequence identity) and an under-representation of Chloroflexi genetic information available for PhyloPythiaS training (Table S3), metagenomic scaffolds originating from Chloroflexi species were not detected and instead assigned as “others” (assignments not extending deeper than Domain-rank). This could explain both inconsistencies, at least in part.

Overall, both analysis yielded a considerable proportion of shallow assignments, with 60% of the PhyloPythiaS assignments (Table S2) and 53% of the OTU lineages (Table S1) not extending deeper than an Order-rank of classification. This confirms that the Svalbard rumen microbiome consists of unique bacterial lineages, with limited similarity to published sequence data from other organisms and/or environments.

### Plant Polysaccharide Degradative enzymes in the Svalbard Reindeer Rumen Microbiome and the Dominant, Novel and Saccharolytic Bacteroidales Clade SRM-1

In order to characterise the biomass-degrading capabilities of the Svalbard reindeer rumen microbiome gene-centric metagenomic datasets were constructed and annotation efforts focused on identifying genes putatively encoding carbohydrate-active genes. Both filtered unassembled reads (Table 1) and assembled contigs and scaffolds (for SRM-1; Table S4) were subjected to automated annotation using the Integrated Microbial Genomes with Microbiome Samples (IMG/MER) system [15]. Carbohydrate-active enzymes were identified using pfamHMMs and grouped according to major functional role (see www.cazy.org and [1] for a description of the classification system).

In total more than 5000 putative GH (Glycoside Hydrolase) gene fragments were recovered from 300 Mb of filtered unassembled reads and over 400 of these were putative cellulases, mainly belonging to families GH5 and GH9 (Table 1). Limited numbers of gene fragments associated with cellulosome complexes were also detected, including genes encoding GH48s (n = 5) as well as cohesin (n = 52) and dockerin (n = 92) modules. Their detection was notable since cellulosome components generally are scantly found in gut metagenomes. However, it is in accordance with reports showing that cellulolytic bacteria associated with cellulosome complexes occur in the microbiome of ruminants [16], including the Svalbard reindeer [8]. The analysis showed a broad profile of hemicellulases and non-cellulase polysaccharide degrading enzymes (Table 1), including a high-proportion of enzymes involved in depolymerisation of the major grass hemicellulose, glucuronoarabinoxylan (e.g. GH51 α-L-arabinofuranosidases and GH67 glucoronidases) and enzymes acting on xylo-oligosaccharides (e.g. GH1–3 and GH43). Despite the snow cover in winter, grass species are still significant components of the Svalbard reindeer diet accounting for 14–26% of daily feed intake [17]. Additionally, many of the identified GH-encoding genes are putatively involved in the deconstruction of polysaccharides that are prevalent in the cell walls of dicots and non-vascular plants. Predicted abundant enzyme activities include hydrolysis of galacturonans (GH28) and rhamnoses (GH78) commonly found in pectin, as well as hydrolysis of mannans (GH5 and GH26) and xyloglucans (GH5, GH16 and GH74). These predicted activities are consistent with the fact that the Svalbard reindeer winter diet is dominated by the dicot *Salix polaris* (dwarf shrub; 4–16%), *Saxifraga* spp. (evergreen; 0–48%) and bryophytes (non-vascular mosses; 10–54%) [17]. Both types of plants possess cell walls that typically have higher levels of xyloglucans, mannans and pectins than grasses [18], [19]. Interestingly the analysis also revealed an abundance of Sus-like genes that may be part of PULs (see Table 1 and below for further discussion).

**Table 1:** Overview of the occurrence of GHs targeting plant structural polysaccharides in five herbivore metagenomes*.

	Predominant activity	Macropod	Termite	Bovine [^6^]	Bovine [^7^]	Reindeer
Cellulases						
GH5	cellulases	10	56	8	1451	287
GH6	endoglucanases	0	0	0	0	0
GH7	endoglucanases	0	0	0	1	0
GH9	endoglucanases	0	9	6	795	109
GH44	endoglucanases	0	6	0	99^†^	5
GH45	endoglucanases	0	4	0	115	0
GH48	cellobiohydrolases	0	0	0	3	5
**Total**		**10 (4)**	**75 (20)**	**14 (2)**	**2464 (13)**	**406 (8)**
**Endohemicellulases**						
GH8	endoxylanases	1	5	4	329	35
GH10	endo-1,4-β-xylanases	11	46	7	1025	190
GH11	xylanases	0	14	1	165	8
GH12	xyloglucanases	0	0	0	0	0
GH26	β-mannanase & xylanases	5	15	5	369	153
GH28	galacturonases	2	6	5	472	120
GH53	endo-1,4-β-galactanases	9	12	17	483^†^	125
**Total**		**28 (10)**	**98 (26)**	**39 (6)**	**2843 (15)**	**631 (12)**
**Xyloglucanases**						
GH16	xyloglucanases	4	1	1	483	116
GH74	xyloglucanases	1	7	0	385^†^	44
**Total**		**5 (2)**	**8 (2)**	**1 (0)**	**868 (5)**	**160 (3)**
**Debranching enzymes**						
GH51	α-L-arabinofuranosidases	12	18	64	1249^†^	488
GH54	α-L-arabinofuranosidases	0	0	1	76^†^	23
GH62	α-L-arabinofuranosidases	0	0	0	1	0
GH67	α-glucuronidases	5	10	0	120	74
GH78	α-L-rhamnosidases	25	0	34	1260	313
**Total**		**42 (15)**	**18 (5)**	**99 (14)**	**2706 (15)**	**898 (17)**
**Oligosaccharide-degrading enzymes**						
GH1	β-glucosidases	61	22	10	253	122
GH2	β-galactosidases	24	23	186	1436	716
GH3	β-glucosidases	72	69	176	2844	844
GH29	α-L-fucosidases	2	0	74	939	268
GH35	β-galactosidases	3	3	12	158	39
GH38	α-mannosidases	3	11	17	272	116
GH39	β-xylosidases	1	3	2	315	76
GH42	β-galactosidases	8	24	11	374	95
GH43	arabino/xylosidases	10	16	61	2932^†^	787
GH52	β-xylosidases	0	3	0	1^†^	2
**Total**		**184 (70)**	**174 (47)**	**549 (78)**	**9524 (52)**	**3065 (60)**
**Other domains associated with GHs**						
Cohesin		0	0	0	80	52
Dockerin		41	0	8	188	92
SusC		36	0	9	3110^¶^	1122
SusD		42	0	11	1889^¶^	685
**Metagenome size**		**0.054 Gb^‡^**	**0.062 Gb^‡^**	**0.026 Gb^§^**	**268 Gb^‡^**	**0.30 Gb^§^**

* GHs are grouped according to their major functional role in the degradation of plant fiber. The numbers in parentheses represent the percentages of these groups relative to the total number of GH’s presented in this table. In addition to GHs, the table shows data for four other selected proteins possibly involved in biomass turnover; see main text for details. Note that some of the GH profiles were derived from contigs^‡^ rather than (filtered) unassembled reads^§^, explaining part of the differences in absolute gene numbers. ¶ Data calculated in this study using searches against the rumen metagenome dataset [7] using SusC (PF00593) and SusD (PF07980) HMMs.^ †^Pfam HMMs not available for CAZy families; data generated by dbCAN (http://csbl.bmb.uga.edu/dbCANdev/index.php).

PhyloPythiaS produced a 0.9 Mb assemblage from the metagenomic contigs and scaffolds it assigned to the dominant SRM-1 Bacteroidales population. More than 20 carbohydrate-active families targeting various hemicelluloses, pectins and cellulose were identified in this assembly, suggesting the presumptive SRM-1 strain is well adapted to the reindeer diet (Table S4). Genes encoding presumptive endoxylanases (GH8 and GH10), β-xylosidases (GH3, GH30 and GH43), α-L-arabinofuranosidases (GH51 and GH53), α-glucuronidases (GH67), and endopolygalacturonase (GH28) and acetyl xylan esterases (CE1 and CE4) were identified. The SRM-1 reassembly also includes presumptive GH5 and GH9 endo-β-1,4-glucanases, and GH3 β-glucosidases, as well as Sus proteins. However, the partial assembly did not contain any known cellobiohydrolases (GH6, GH7, or GH48), nor were any dockerin or cohesin modules present; suggesting a non-cellulosomal mode of polysaccharide hydrolysis. Interestingly, a putative polyphenol oxidoreductase laccase was also identified and suggests that, along with the presence of CE1 and CE4 esterases, this bacterium may have an effective strategy for the deconstruction of “non-core” lignin.

### Functional Screens and Gene-mining Linking Cellulose Degradation to the Bacteroidales and PULs

**Figure 2:**
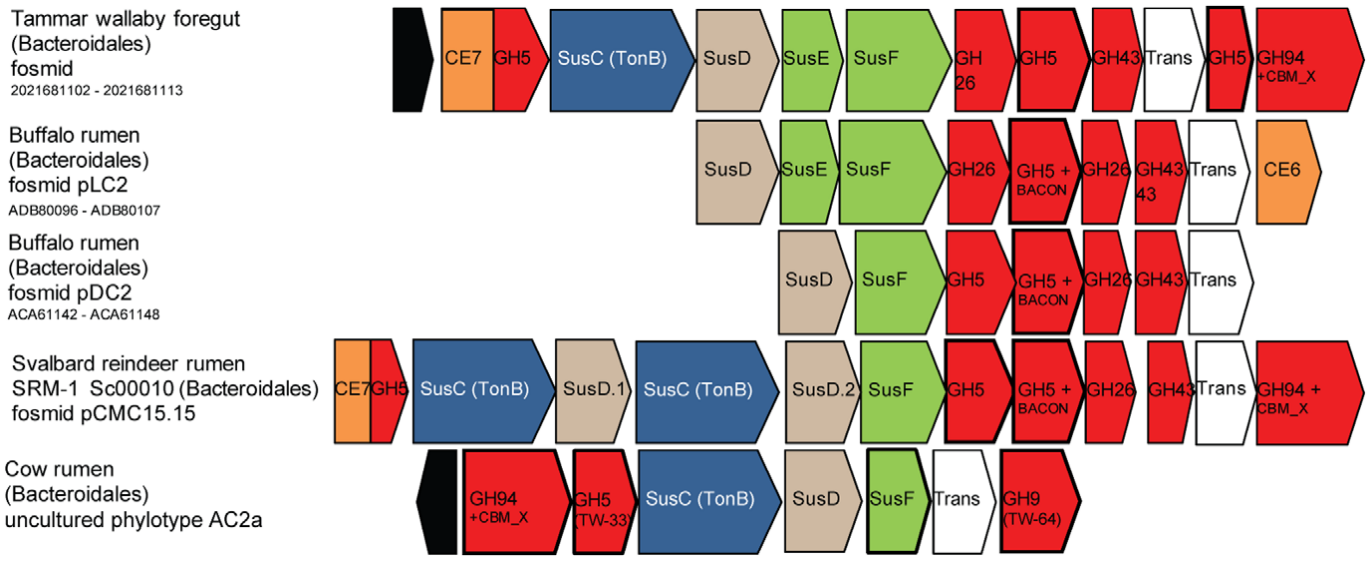
Comparative gene organization of presumptive cellulolytic PULs identified in the Svalbard reindeer rumen and other gut environments. Gene clusters were recovered from the cow rumen metagenome (partial genome of an as-yet uncultured Bacteroidales phylotype AC2a) and sequenced cellulolytic fosmids constructed from environmental DNA originating from the gut microbiomes of the wallaby foregut and the reindeer and buffalo rumen (upper four clusters). All fosmid sequences originate from Bacteroidetes, according to PhyloPythiaS binning, and for all cellulolytic activity has been detected in functional screens. Green genes represent SusE/SusF-like genes predicted to encode outer-membrane proteins whose function is currently unknown. Black genes encode putative response-regulators. BACON: indicates a carbohydrate binding domain identified by [40]. TonB: indicates members of the TonB-dependent receptor family, a group of outer membrane spanning β-barrel proteins that transport solutes and macromolecules. TW-64 and TW-33 correspond to sample IDs for the GH9 and GH5 genes (respectively) encoded within the AC2a PUL, from which expressed proteins were tested positive for hydrolytic activity on various cellulosic substrates by Hess et al., [7]. GenBank accession numbers and/or IMG Gene Object ID numbers are provided. Gene IDs for the AC2a PUL are provided in Table S5.

The frequency of fosmid clones testing positive for CMCase activity was the highest reported so far (48 positive from ∼5,000 screened, corresponding to ∼1% of the clones and a hit rate of 1 per 3.5 Mb screened; [20]). The majority of the sequenced fosmid clones were predicted to be derived from Bacteroidetes-affiliated lineages (Table 2 **)**. More than 70% of the scaffolds constructed carry one or more genes encoding a presumptive GH5 endoglucanase. However, two scaffolds (Sc00001 & Sc Sc00021) did not possess any GH families representing known endoglucanases, suggesting members of the Bacteroidales might also possess novel cellulose-degrading mechanisms. Further studies, e.g. employing random transposon mutagenesis, are needed to identify exactly which genes are responsible for the enzymatic activity encoded by these fosmids. The presumptive GH5 endoglucanase genes were most often located in close proximity to multi-gene PULs which were all found on fosmids assigned to Bacteroidetes, including one fosmid assigned to SRM-1 (see Figure 2, Table 2). Their gene-organisation included Sus-like outer membrane proteins homologous to SusC and SusD, which are essential for the import and degradation of starch by the Sus initially described in the human gut bacterium *Bacteroides thetaiotaomicron* [21]. SusC- and SusD- like genes which typify a Sus-like PUL, function together with other hypothetical outer-membrane proteins (SusE/F-like) and linked carbohydrate-active enzymes [11]. Whilst originally characterised on starch, Sus-like PULs encoded in human gut Bacteroidetes have since been described with capabilities to degrade other plant polysaccharides that include pectins and hemicellulosic substrates [22]. Predictions of polysaccharide degradation by Sus-like PULs in other environments have previously been made [11], [23], but so far there are only few studies addressing their importance in the herbivore gut. Their involvement in xylan degradation by the rumen bacterium *Prevotella bryantii* has been illustrated by gene transcriptome studies [23], whereas their potential role in cellulose degradation has been suggested on the basis of the metagenome described for the anaerobic microbiome of the Tammar wallaby foregut [5].

**Table 2:** Summary of glycoside hydrolases and Sus proteins encoded within scaffolds reconstructed from sequenced fosmids selected from functional genomic screens.

Scaffold	GH2	GH3	GH5	GH9	GH10	GH16	GH20	GH23	GH26	GH28	GH29	GH31	GH33	GH35	GH43	GH51	GH53	GH55	GH74	GH76	GH78	GH94	GH109	GH117	PL1	PL22	CE1	CE7	CE11	*susC*	*susD*	*susE/F*	Binning	Sb.
Sc00001																																	SRM-1	C
Sc00002	1		1					1	2																			2	1	1	1	2	Prevotellaceae	C
Sc00003			2						1		1																1						Bacteroidaceae	C
Sc00004	1		1	1				1			1			1	1								1				1			2			Bacteroidaceae	C
Sc00005			1				1																	1						1	1	2	Prevotellaceae	C
Sc00006			2^BACON^																											1	1		Rikenellaceae	C
Sc00007	1	3			1										1							1^CBM_X^					3						Bacteroidaceae	X
Sc00008	1		2^BACON^							1			1			1^CBM4^		1	1						2					1	1	2	Prevotellaceae	C
Sc00009			1										1													1	1						Rikenellaceae	C
Sc00010	1		3^BACON^						1						1							1^CBM_X^					1	1		2	2	1	SRM-1	C
Sc00011			1																														Bacteroidaceae	C
Sc00012	1		2						1^BACON^						1												1						Bacteroidaceae	C
Sc00013																1																	SRM-1	C
Sc00015					1^CBM6x3^					1		1									1												Prevotellaceae	X
Sc00016			1^BACON^																	2	1									1	1	2	Clostridiales	C
Sc00017			1																														Ruminococcaceae	C
Sc00018					1^CBM6^										2^CBM6^	1^CBM4^																	Prevotellaceae	X
Sc00019		1																															SRM-1	C
Sc00020			2 ^BACON^						2						1					1								1		1	1	1	Prevotellaceae	C
Sc00021																	1													1	1	1	Bacteroidaceae	C
Sc00022			1			1																											δ-proteobacteria	C
Sc00023			1								1	1																					Clostridiales	C
Sc00024						1																											Prevotellaceae	C
Sc00025		1	2 ^BACON^						1 ^BACON^						1															1	1	2	Bacteroidaceae	C
Sc00026			2 ^BACON^						2 ^BACON^																					1	1	2	Bacteroidaceae	C
Sc00028			2 ^BACON^																														Bacteroidaceae	C
Sc00031	1	1	1						1			2																					Rikenellaceae	C

**Sb.** Substrate. **C** Cellulose degrading activity detected using carboxymethyl cellulose as a substrate. **X** Xylan degrading activity detected using birchwood xylan as a substrate. Carbohydrate binding modules (CBM or BACON) are indicated in superscript text. Note that Sc00001 and Sc00021 do not contain any gene(s) coding for proteins known to be involved in the degradation of cellulose. Partial scaffolds not encoding documented cellulose active genes are excluded (n = 13).

Having observed the abundance of Sus-like proteins in the Svalbard reindeer microbiome, we re-evaluated existing herbivore metagenomic data. Cellulolytic screens using fosmids constructed from the metagenome of the buffalo rumen microbiome have previously identified GH5 endoglucanases affiliated to the Bacteroidetes [12]; closer examination of flanking genetic regions revealed the presence of Sus-like PULs (Figure 2). Gene-mining within the rumen metagenome constructed by Hess et al. [7], revealed a hitherto non-detected abundance of Sus-like genes (Table 1) as well as a cellulase-linked PUL encoded within the partial genome assembled for the as-yet uncultured Bacteroidetes phylotype AC2a (Figure 2, Table S5). The cellulases (GH5 and GH9) and a GH94 cellobiose phosphorylase encoded within the AC2a PUL suggest activity against cellulosic substrates (Figure 2). Interestingly, Hess et al. showed that both these cellulase genes expressed proteins with enzymatic activity against either CMC or avicel and pretreated *Miscanthus* substrates [7] (Table S5). Overall these findings suggest that PULs including those linked with cellulases are widespread in herbivore gut environments and may play a major role with respect to plant biomass degradation, similar to what has been demonstrated in the human distal gut. Taken together the accumulated data also suggests involvement of Sus-like proteins in cellulose degradation, a process which is not predominant in the human gut but essential in herbivores.

### Concluding Remarks

The high-Arctic Svalbard reindeer survive under austere nutritional conditions relying on the ability of the rumen microbiome to digest poor quality mosses and fibrous plants available in winter. The metagenomic analyses in this study illustrate that the Svalbard reindeer rumen is host to novel and numerically dominant bacterial lineages. The deeply branched and numerically dominant SRM-1 lineage is predicted to play a key role in the deconstruction of plant biomass by producing an array of glycoside hydrolases targeting cellulose, hemicelluloses, pectin and other oligosaccharides. Functional screening and sequencing of fosmid libraries revealed CMCase positive clones affiliated to the abundant SRM-1 clade that lacked known endoglucanases, inferring the possibility of novel cellulolytic mechanisms that are yet to be characterised. This approach also identified Bacteroidetes-affiliated GH5-linked PULs, which included one linked to SRM-1. The finding of similar cellulase-linked PULs in the cow and buffalo rumen and macropod foregut microbiomes adds further weight to the hypothesis that these structures perhaps represent a key adaptation to growth on cellulose by Bacteroidetes species. It would seem that these PULs and their constituent genes present important targets for further research, possibly representing completely novel mechanisms for enzymatic cellulose conversion in anaerobic gut environments.

## Materials and Methods

### Reindeer Sampling and Ethics Statement

Rumen contents were sampled from two adult, female Svalbard reindeer (*Rangifer tarandus platyrhynchus*) (SR1 and SR2) aged between 1.5 and 4.5 years and grazing on their natural winter pastures in Bjørndalen near Longyearbyen, Svalbard (Norway) on January 31^st^ 2010. The Svalbard Environmental Protection Fund “Svalbard miljøvernfond,” Acting Environment Manager Per Kyrre Reymert and Conservation Advisor Tor Punsvik, approved the use of Svalbard Reindeer in this study (Permission reference number: 2009/00420-4). The Svalbard Environmental Protection Fund is the competent authority for sampling from wild animal populations. The reindeer were euthanized by an experienced hunter by shooting followed by exsanguination, which is an approved procedure under the Norwegian Animal Welfare Act. Since the reindeer were euthanized for the post mortem collection of samples, the procedure did not require ethical approval from the Norwegian Animal Research Authority (NARA).

In addition, for comparative *rrs* analysis, two adult Norwegian reindeer (*Rangifer tarandus tarandus*) (NR1 and NR2) fed a commercially available pelleted concentrate feed for reindeer (RF-80; Felleskjøpet, Norway), were sampled from the domestic herd at the University of Tromsø, Norway (December 2009). NR1 and NR2 were purchased from local reindeer herders and maintained in the animal research facility at the University of Tromsø. This facility (and staff) has been inspected and approved by NARA, and therefore fulfills current animal welfare criteria. The reindeer were euthanized by trained personnel by stunning followed by exsanguination, which is an approved procedure under the Norwegian Animal Welfare Act.

Rumen contents were transferred immediately after slaughter to sterile containers and frozen at −80°C.

### Cell Dissociation and DNA Extraction

Cell dissociation from plant material and DNA extraction were performed on individual samples (SR1, SR2, NR1 and NR2) and pooled Svalbard reindeer samples that contained equal amounts of SR1 and SR2 material. To desorb and recover microbes adhered to plant biomass 5–10 g of the pooled samples were centrifuged at 14 000 rpm for 2 minutes, and the pellets were resuspended in dissociation buffer and subjected to a dissociation and DNA extraction procedure described by Rosewarne et al. [24].

### 16S rRNA Gene Amplicon Sequencing

Bacterial *rrs* genes were amplified from the individual NR1, NR2, SR1 and SR2 metagenomic DNA samples using the forward primer (5′- CCT ATC CCC TGT GTG CCT TGG CAG TCT CAG CAA CAG CTA GAG TTT GAT CCT GG -3′), which contained the 454 Life Sciences primer B sequence and the broadly conserved bacterial primer 27F, and the reverse primer (5′-CCA TCT CAT CCC TGC GTG TCT CCG ACT CAG NNN NNN NNT TAC CGC GGC TGC T -3′), which contained the 454 Life Sciences primer A sequence, the broadly-conserved bacterial primer 515R and a unique 8-nt multiplex identifier (MID) used to tag each amplicon (designated by NNNNNNNN) [25]. Four technical replicate PCR reactions (each with a unique barcode) were performed for each DNA sample. Pyrosequencing of *rrs* gene amplicons was performed on the 454 Genome Sequencer FLX-Titanium system according to manufacturer’s instructions (454 Life Sciences). Signal processing and base calling were performed using the bundled 454 Data Analysis Software version 2.3.

### Phylogenetic Analysis of 16S rRNA Gene Sequences

*Rrs* gene sequences were processed using the QIIME software package [26] and removed from the analysis if they were <350 or >550 nt in length, contained ambiguous bases, had a mean quality score <25, contained a homopolymer run exceeding 6 nt, or did not contain a primer or barcode sequence. Similar sequences were clustered into operational taxonomic units (OTUs) using UCLUST software [27] and a 97% sequence identity threshold. To eliminate noise and possible artifacts introduced during PCR and sequencing, OTUs were filtered so that only those that contained representatives from a minimum of 4 samples were used, and the most abundant sequence in each OTU was chosen as the representative sequence. As an added precaution chimeras were removed from the representative set using Chimera Slayer as previous work suggests that chimera formation is reproducible across technical replicates [28]. Representative sequences (accession numbers JN802705 - JN803885, SRM-1: JN802985) were aligned against the Greengenes core set [29] using PyNAST software [30] with a minimum alignment length of 150 and a minimum identity of 75%. Taxonomy was assigned to each OTU using the Ribosomal Database Project (RDP) classifier [13] with a minimum support threshold of 80% and using the RDP taxonomic nomenclature. The alignments were then filtered to remove gaps and hypervariable regions using a Lane mask [26], and an maximum-likelihood tree was constructed from the filtered alignment using FastTree [31]. Prior to comparison of reindeer *rrs* gene sequences with wallaby, rumen and termite samples, each *rrs* dataset was randomly “subsampled” using QIIME to normalize each dataset and remove sample heterogeneity. An unweighted UniFrac distance matrix [32] was constructed from the phylogenetic tree and visualised using principal coordinates analysis. The OTU network maps were generated using QIIME and visualised with Cytoscape [33].

### Metagenome Processing: Shotgun Library Preparation, Sequencing and Assembly

Shotgun sequencing runs were performed on libraries prepared from pooled Svalbard reindeer rumen community DNA using the 454 Genome Sequencer FLX-Titanium single- and paired-end protocols (total 1,453,100 reads, 503 Mb). Sequencing reads were assembled using Newbler (GSassembler v. 2.3) resulting in 32,073 contigs ≥500 nt, totalling 26 Mb and 1364 scaffolds totalling 5.44 Mb. Due to low assembly (334,500 out of 1,453,100: ∼23%), unassembled single-end reads were used for community GH profile analysis. Importantly this approach also ensured that differences in species abundance distribution were incorporated (i.e. dominant populations producing multiple hits to the same gene will be weighted in the analysis). Unassembled sequencing reads with degenerate bases (“Ns”) were removed along with all replicate sequences that were detected using the following parameters: 0.9 (90% ID), length difference requirement  = 0 and 3 beginning bases checked [34]. A total of 695,636 (300 Mb) reads passed this quality filtering. The raw sequencing reads and the assembled metagenome dataset have been deposited at the NCBI Short Read Archive under BioProject ID PRJNA73677 and accession number SRA046345.1.

### Fosmid Constructioxn, Screening, Sequencing and Assembly

A 36 kb insert fosmid library was cloned in pCC1Fos (Epicentre Corp.) using previously described methods [24]. Fosmid clones bearing endoglucanase and/or xylanase activity were detected by plating the *E. coli* library on LB-chloramphenicol agar plates containing either 0.2% (w/v) carboxymethylcellulose or 0.2% (w/v) birchwood xylan (Sigma). Recombinant strains were plated and incubated overnight at 37°C. The plates were then stained with Congo red dye and de-stained with 1M NaCl to reveal zones of hydrolysis. Positive colonies were isolated and reexamined to confirm activity. This approach yielded 48 fosmid clones positive for carboxymethylcellulose hydrolysis (∼5,000 screened) and three positive for xylan hydrolysis (∼1,000 screened). These 51 clones were subsequently sequenced and assembled. Fosmid copy numbers were enhanced using Epicentre protocols, and the fosmid DNA was purified using Qiagen MiniPrep columns. Equimolar amounts of the fosmids were pooled together (∼20 µg total DNA) and both a 3 kb paired-end library and a 454 standard shotgun library were constructed. Both libraries were directly sequenced with the 454 Life Sciences Genome Sequencer GS FLX and assembled into 40 scaffolds using Newbler. Redundancy was observed within the assembly with several fosmids overlapping at least one other fosmid, however no instances of fosmid replicates were observed. For nine fosmids, insert sequences were completely assembled, with no gaps. In total, 1.2 Mb of metagenomic DNA sequence was assembled and manually edited.

### Gene Annotation and Phylogenetic Analysis

Three metagenomic datasets (unassembled single-end reads, assembled contigs ≥500 nt, and assembled fosmids) from the Svalbard reindeer rumen microbiome were annotated via the IMG/M-ER annotation pipeline and loaded as independent data sets into IMG/M-ER [15] (http://img.jgi.doe.gov/cgi-bin/m/main.cgi), a data-management and analysis platform for genomic and metagenomic data based on IMG [35]. Complete annotated data for the fosmid scaffolds, assembled contigs and unassembled reads can also be accessed through the IMG/MER (http://img.jgi.doe.gov) under Taxon Object ID 2199352020, 2081372005 and 2088090000 respectively. Putative genes were called with a combination of GeneMark.hmm for Prokaryotes (v. 2.4) [36], MetaGene [37], Prodigal (v2.00) [38] and multiBLASTx. Datasets from the Hess et al., rumen metagenome [7] were downloaded from ftp://ftp.jgi-psf.org/pub/rnd2/Cow_Rumen/. This included genome bins for as-yet uncultured bacteria (ftp://ftp.jgi-psf.org/pub/rnd2/Cow_Rumen/cow_rumen_genome_bins.tar.gz) and putative genes for the total dataset that included genome bins as well as assembled metagenome contigs (ftp://ftp.jgi-psf.org/pub/rnd2/Cow_Rumen/metagenemark_predictions.faa.gz).

Searches for glycoside hydrolases (GHs) of selected functional classes (e.g. cellulases, hemicellulases, debranching enzymes, “others”) were performed with pfam HMMs (Pfam version 24.0 and HMMER v3.0), named in accordance with the CAZy nomenclature scheme [1]. The specific cut-off was set to Gathering Threshold (HMMER). For those GH families for which there is currently no representation in Pfam, HMMs were generated using hmmbuild (HMMER) and multiple sequence alignments of representative sequences selected from the CAZy database.

## Binning

Assembled metagenomic contigs, scaffolds and fosmids were binned (classified) using PhyloPythiaS [10], a kmer-based taxonomic classifier. The classifier was trained to include clades at the taxonomic ranks of domain, phylum, class, order and family and the clade “uncultured Bacteroidales bacterium” (SRM-1). The models include all clades covered by two or more species at the corresponding ranks among 2193 sequenced microbial isolates and clade SRM-1 (Table S3). The classifier consists of an ensemble of six structural support vector machines (SVMs) models, created by using fragments of 1, 3, 5, 10, 15 and 50 kb in length, respectively, for training (see [10] for details). For SRM-1, thirteen assembled scaffolds (a total of 205,517 bp) were used for training, which were assigned unambiguously through a combination of high read coverage, consistent GC% and affiliation of selected phylogenetic marker genes [39] to the order Bacteroidales. Sample specific sequences for other clades were obtained by similarity searches and application of the lowest common ancestor algorithm on taxonomic identifiers of the best-scoring hits. Input fragments of a particular length were generated by using a sliding window with a step size of one-tenth of the generated fragment size (for example 5 kb for 50-kb fragments) on sample-derived sequences and a step size of generated fragment length on the isolate sequences. The classifier was then used to assign all assembled scaffolds and contigs larger than 500 bp. Results of this binning process were loaded into IMG/M-ER to allow independent analysis of the component populations.

## Supporting Information

Table S1 **Operational taxonomic units (OTU) representatives of 16S rRNA gene sequences obtained from the rumen microbiome of the Svalbard reindeer.** * Hierarchical taxonomic assignment for each OTU calculated using the RDP naïve Bayesian Classifier [13]. Lineages are displayed only where OTUs could be assigned with an 80% bootstrap confidence estimate. SR1 and SR2 indicate animal number and a-d indicate PCR replicates used for OTU filtering (see Materials and Methods). Rows highlighted in yellow indicate OTUs shared with all ruminant and foregut samples (see Text and Figure 1b)
(DOC)

Table S2 **Phylogenetic profile of the Svalbard reindeer rumen metagenome sequence dataset, based on sequence composition-based binning of assembled scaffolds using PhyloPythiaS.** Values described in non-bold text represent sub-category counts for order/family lineages within the Bacteroidetes and Firmicutes.
(DOC)

Table S3 **Input clades, sample specific data and genomes/Whole Genome Shotgun submissions (WGS) used for PhyloPythiaS training models.** * temporary ncbid for SRM-1.
(DOC)

Table S4 **Glycoside hydrolases and related proteins recovered from the putative partial SRM-1 genome.** * indicates best match; † indicates contig is linked to an uncultured *Bacteroidales* bacterium scaffold, based on PhyloPythiaS analysis; +SP indicates signal peptide detected. Taxonomic assignment was predicted using GC%, high coverage (greater than 6x) and PhyloPythiaS binning.
(DOC)

Table S5 **A cellulase-linked PUL encoded within the as-yet uncultured Bacteroidales phylotype AC2a genome bin, reconstructed from the rumen metagenome** [**7**] **.** * Gene ID’s are in the following format: NODE_ORF (see [7]). All data downloaded from ftp://ftp.jgi-psf.org/pub/rnd2/Cow_Rumen/† Sample ID and Substrate are as in Figure 3 and Table S6 from Hess et al. [7].
(DOC)

Figure S1 **Relative abundance of the 20 most dominant bacterial taxa in the Svalbard reindeer rumen microbiome.** Percentages are calculated against the total number of 16S rRNA gene sequences recovered. The closest cultured relative of each OTU and the sequence similarity % ID is indicated in parentheses. The lineage of each OTU is indicated by colour of text: Bacteroidetes maroon, Chloroflexi blue and Firmicutes green.
(TIF)
