# Correction: Metagenomics of the Svalbard Reindeer Rumen Microbiome Reveals Abundance of Polysaccharide Utilization Loci

**DOI:** 10.1371/journal.pone.0104612

**Published:** 2014-07-31

**Authors:** The PLOS ONE Staff

The columns in Table 2 are misaligned. Please see the corrected Table 2, labelled File S1, in the Supporting Information section of this correction.

## Supporting Information

File S1 Corrected Table 2.
(DOC)
