# Supplementary material for: Metagenomics of the Svalbard Reindeer Rumen Microbiome Reveals Abundance of Polysaccharide Utilization Loci
Source: PLoS One. 2012 Jun 6;7(6):e38571. doi: 10.1371/journal.pone.0038571 (PMC3368933; doi:10.1371/journal.pone.0038571)
Supplement: Table S1 — Operational taxonomic units (OTU) representatives of 16S rRNA gene sequences obtained from the rumen microbiome of the Svalbard reindeer. * Hierarchical taxonomic assignment for each OTU calculated using the RDP naïve Bayesian Classifier [13]. Lineages are displayed only where OTUs could be assigned with an 80% bootstrap confidence estimate. SR1 and SR2 indicate animal number and a-d indicate PCR replicates used for OTU filtering (see Materials and Methods). Rows highlighted in yellow indicate OTUs shared with all ruminant and foregut samples (see Text and Figure 1b) (DOC) [file pone.0038571.s001.doc]

**Table S1. Operational taxonomic units (OTU) representatives of 16S rRNA gene sequences obtained from the rumen microbiome of the Svalbard reindeer.**

| **OTU ID** | **aSR1** | | **bSR1** | **cSR1** | **dSR1** | **aSR2** | **bSR2** | **cSR2** | **dSR2** | **TOTAL** | | **%** | | **Consensus Lineage*** |
| --- | --- | --- | --- | --- | --- | --- | --- | --- | --- | --- | --- | --- | --- | --- |
| SRM-1 | | 86 | 137 | 122 | 175 | 938 | 1376 | 1186 | 1395 | 5415 | | 11.3 | | Bacteroidetes;Bacteroidetes;Bacteroidales |
| 13505 | 97 | | 199 | 135 | 216 | 389 | 581 | 534 | 536 | 2687 | | 5.60 | | Bacteroidetes |
| 1382 | 143 | | 194 | 179 | 271 | 280 | 370 | 327 | 351 | 2115 | | 4.41 | | Bacteroidetes |
| 7594 | 210 | | 285 | 290 | 357 | 0 | 0 | 0 | 1 | 1143 | | 2.38 | | Bacteroidetes;Bacteroidetes;Bacteroidales;Prevotellaceae |
| 12349 | 9 | | 14 | 10 | 21 | 228 | 294 | 231 | 326 | 1133 | | 2.36 | | Bacteroidetes;Bacteroidetes;Bacteroidales;Prevotellaceae;Prevotella |
| 12909 | 34 | | 63 | 53 | 84 | 174 | 234 | 178 | 258 | 1078 | | 2.25 | | Bacteroidetes;Bacteroidetes;Bacteroidales;Prevotellaceae |
| 488 | 15 | | 35 | 20 | 25 | 181 | 243 | 203 | 258 | 980 | | 2.04 | | Bacteroidetes;Bacteroidetes;Bacteroidales;Prevotellaceae;Prevotella |
| 2509 | 77 | | 97 | 84 | 105 | 123 | 151 | 106 | 155 | 898 | | 1.87 | | Chloroflexi;Anaerolineae |
| 3260 | 83 | | 115 | 91 | 119 | 77 | 122 | 87 | 110 | 804 | | 1.68 | | Chloroflexi;Anaerolineae |
| 3042 | 9 | | 21 | 13 | 28 | 113 | 160 | 134 | 190 | 668 | | 1.39 | | Bacteroidetes;Bacteroidetes;Bacteroidales;Prevotellaceae;Prevotella |
| 11748 | 54 | | 117 | 94 | 112 | 46 | 67 | 63 | 62 | 615 | | 1.28 | | Firmicutes;Clostridia;Clostridiales |
| 15858 | 113 | | 123 | 126 | 183 | 0 | 5 | 3 | 1 | 554 | | 1.15 | | Bacteroidetes |
| 15278 | 89 | | 112 | 101 | 133 | 5 | 7 | 2 | 2 | 451 | | 0.94 | | Bacteroidetes |
| 7193 | 0 | | 3 | 7 | 7 | 74 | 107 | 95 | 121 | 414 | | 0.86 | | Bacteroidetes;Bacteroidetes;Bacteroidales;Prevotellaceae;Prevotella |
| 1166 | 22 | | 39 | 27 | 56 | 43 | 92 | 59 | 68 | 406 | | 0.85 | | Bacteroidetes;Bacteroidetes;Bacteroidales;Prevotellaceae;Prevotella |
| 12979 | 49 | | 70 | 51 | 91 | 20 | 47 | 31 | 35 | 394 | | 0.82 | | Bacteroidetes;Bacteroidetes;Bacteroidales |
| 3732 | 70 | | 102 | 95 | 93 | 1 | 16 | 6 | 10 | 393 | | 0.82 | | Bacteroidetes;Bacteroidetes;Bacteroidales;Porphyromonadaceae |
| 7817 | 3 | | 4 | 6 | 9 | 73 | 116 | 84 | 88 | 383 | | 0.80 | | Bacteroidetes;Bacteroidetes;Bacteroidales;Prevotellaceae;Prevotella |
| 11299 | 30 | | 33 | 18 | 39 | 46 | 77 | 47 | 88 | 378 | | 0.79 | | Bacteroidetes |
| 14681 | 34 | | 68 | 64 | 82 | 17 | 34 | 16 | 31 | 346 | | 0.72 | | Bacteroidetes;Bacteroidetes;Bacteroidales |
| 2541 | 2 | | 6 | 2 | 4 | 66 | 91 | 59 | 80 | 310 | | 0.65 | | Bacteroidetes;Bacteroidetes;Bacteroidales;Prevotellaceae |
| 6540 | 2 | | 7 | 6 | 5 | 47 | 89 | 74 | 72 | 302 | | 0.63 | | Bacteroidetes;Bacteroidetes;Bacteroidales;Prevotellaceae;Prevotella |
| 15723 | 6 | | 6 | 9 | 11 | 35 | 79 | 78 | 78 | 302 | | 0.63 | | Bacteroidetes |
| 11365 | 22 | | 55 | 28 | 47 | 38 | 30 | 38 | 43 | 301 | | 0.63 | | Bacteroidetes;Bacteroidetes;Bacteroidales;Prevotellaceae;Hallella |
| 1873 | 29 | | 40 | 51 | 56 | 23 | 18 | 18 | 37 | 272 | | 0.57 | | Firmicutes;Clostridia;Clostridiales;Lachnospiraceae |
| 1206 | 16 | | 30 | 14 | 36 | 38 | 42 | 58 | 26 | 260 | | 0.54 | | Firmicutes;Clostridia;Clostridiales;Veillonellaceae;Succiniclasticum |
| 21379 | 35 | | 56 | 64 | 73 | 2 | 12 | 4 | 9 | 255 | | 0.53 | | Bacteroidetes;Bacteroidetes;Bacteroidales;Prevotellaceae |
| 73 | 20 | | 30 | 34 | 33 | 33 | 35 | 37 | 28 | 250 | | 0.52 | | Firmicutes;Clostridia;Clostridiales;Ruminococcaceae |
| 13292 | 30 | | 33 | 34 | 33 | 27 | 39 | 19 | 35 | 250 | | 0.52 | | Chloroflexi;Anaerolineae |
| 4068 | 16 | | 30 | 23 | 36 | 26 | 30 | 35 | 44 | 240 | | 0.50 | | Bacteroidetes;Bacteroidetes;Bacteroidales;Prevotellaceae;Prevotella |
| 20574 | 2 | | 3 | 1 | 5 | 45 | 51 | 53 | 72 | 232 | | 0.48 | | Bacteroidetes;Bacteroidetes;Bacteroidales |
| 4986 | 39 | | 59 | 50 | 59 | 4 | 8 | 3 | 5 | 227 | | 0.47 | | Bacteroidetes;Bacteroidetes;Bacteroidales;Prevotellaceae;Prevotella |
| 18154 | 25 | | 54 | 62 | 71 | 0 | 0 | 0 | 0 | 212 | | 0.44 | | Bacteroidetes;Bacteroidetes;Bacteroidales |
| 784 | 8 | | 15 | 8 | 13 | 38 | 46 | 43 | 39 | 210 | | 0.44 | | Firmicutes;Clostridia |
| 5724 | 3 | | 6 | 7 | 11 | 40 | 47 | 32 | 57 | 203 | | 0.42 | | Firmicutes;Clostridia;Clostridiales |
| 14049 | 22 | | 24 | 14 | 30 | 19 | 38 | 25 | 29 | 201 | | 0.42 | | Firmicutes;Clostridia;Clostridiales;Lachnospiraceae |
| 19572 | 32 | | 44 | 52 | 49 | 2 | 1 | 5 | 3 | 188 | | 0.39 | | Bacteroidetes;Bacteroidetes;Bacteroidales;Prevotellaceae;Hallella |
| 14674 | 16 | | 17 | 12 | 13 | 28 | 17 | 26 | 49 | 178 | | 0.37 | | Bacteroidetes;Bacteroidetes;Bacteroidales;Prevotellaceae;Prevotella |
| 1156 | 13 | | 21 | 20 | 35 | 12 | 30 | 18 | 24 | 173 | | 0.36 | | Firmicutes;Clostridia;Clostridiales;Ruminococcaceae |
| 18824 | 12 | | 11 | 10 | 11 | 21 | 50 | 27 | 28 | 170 | | 0.35 | | Bacteroidetes;Bacteroidetes;Bacteroidales |
| 20726 | 21 | | 27 | 28 | 46 | 8 | 14 | 6 | 19 | 169 | | 0.35 | | Chloroflexi;Anaerolineae |
| 18648 | 2 | | 3 | 1 | 6 | 30 | 49 | 32 | 39 | 162 | | 0.34 | | Bacteroidetes;Bacteroidetes;Bacteroidales;Porphyromonadaceae |
| 23595 | 8 | | 5 | 6 | 15 | 23 | 33 | 22 | 49 | 161 | | 0.34 | | Bacteroidetes;Bacteroidetes;Bacteroidales;Prevotellaceae;Prevotella |
| 17741 | 13 | | 15 | 10 | 8 | 18 | 38 | 27 | 31 | 160 | | 0.33 | | Firmicutes;Clostridia;Clostridiales;Lachnospiraceae |
| 14077 | 8 | | 4 | 9 | 24 | 22 | 33 | 25 | 33 | 158 | | 0.33 | | Firmicutes;Clostridia;Clostridiales;Lachnospiraceae; Pseudobutyrivibrio |
| 14071 | 5 | | 19 | 9 | 9 | 22 | 33 | 32 | 25 | 154 | | 0.32 | | Firmicutes;Clostridia;Clostridiales;Ruminococcaceae |
| 7156 | 14 | | 18 | 17 | 20 | 12 | 26 | 16 | 28 | 151 | | 0.31 | | Firmicutes;Clostridia;Clostridiales;Lachnospiraceae |
| 10756 | 10 | | 20 | 17 | 28 | 13 | 17 | 17 | 20 | 142 | | 0.30 | | Bacteroidetes;Bacteroidetes;Bacteroidales;Prevotellaceae;Prevotella |
| 19168 | 0 | | 0 | 1 | 1 | 30 | 37 | 31 | 40 | 140 | | 0.29 | | Bacteroidetes;Bacteroidetes;Bacteroidales |
| 5958 | 2 | | 6 | 8 | 8 | 20 | 29 | 30 | 35 | 138 | | 0.29 | | Firmicutes;Clostridia |
| 23158 | 12 | | 28 | 12 | 21 | 16 | 16 | 13 | 18 | 136 | | 0.28 | | Bacteria |
| 15763 | 16 | | 17 | 16 | 32 | 8 | 17 | 10 | 20 | 136 | | 0.28 | | Firmicutes;Clostridia;Clostridiales;Lachnospiraceae |
| 22109 | 21 | | 37 | 37 | 41 | 0 | 0 | 0 | 0 | 136 | | 0.28 | | Bacteroidetes;Bacteroidetes;Bacteroidales |
| 984 | 17 | | 23 | 22 | 34 | 13 | 9 | 9 | 6 | 133 | | 0.28 | | Firmicutes;Clostridia;Clostridiales;Lachnospiraceae |
| 12542 | 7 | | 22 | 15 | 24 | 11 | 16 | 15 | 16 | 126 | | 0.26 | | Firmicutes |
| 15744 | 5 | | 17 | 13 | 24 | 9 | 19 | 16 | 19 | 122 | | 0.25 | | Bacteroidetes;Bacteroidetes;Bacteroidales;Rikenellaceae;Rikenella |
| 1295 | 13 | | 17 | 22 | 22 | 15 | 8 | 13 | 8 | 118 | | 0.25 | | Bacteroidetes;Bacteroidetes;Bacteroidales |
| 10084 | 0 | | 2 | 1 | 0 | 25 | 27 | 25 | 34 | 114 | | 0.24 | | Bacteroidetes;Bacteroidetes;Bacteroidales;Prevotellaceae;Prevotella |
| 11838 | 10 | | 25 | 22 | 45 | 3 | 2 | 3 | 1 | 111 | | 0.23 | | Bacteroidetes;Bacteroidetes;Bacteroidales |
| 23225 | 2 | | 4 | 3 | 2 | 32 | 26 | 18 | 21 | 108 | | 0.23 | | Firmicutes;Clostridia;Clostridiales;Lachnospiraceae |
| 3243 | 8 | | 23 | 3 | 19 | 7 | 18 | 11 | 17 | 106 | | 0.22 | | Bacteroidetes;Bacteroidetes;Bacteroidales |
| 12675 | 0 | | 1 | 2 | 7 | 22 | 26 | 19 | 27 | 104 | | 0.22 | | Firmicutes;Clostridia;Clostridiales;Lachnospiraceae |
| 6538 | 3 | | 6 | 1 | 6 | 18 | 31 | 17 | 20 | 102 | | 0.21 | | Firmicutes;Clostridia;Clostridiales;Ruminococcaceae |
| 19551 | 15 | | 22 | 18 | 31 | 3 | 1 | 3 | 3 | 96 | | 0.20 | | Bacteroidetes |
| 4058 | 0 | | 2 | 3 | 0 | 19 | 23 | 25 | 23 | 95 | | 0.20 | | Bacteroidetes;Bacteroidetes;Bacteroidales;Prevotellaceae |
| 2336 | 9 | | 13 | 8 | 17 | 7 | 16 | 8 | 17 | 95 | | 0.20 | | Bacteroidetes;Bacteroidetes;Bacteroidales |
| 11795 | 6 | | 17 | 13 | 18 | 7 | 15 | 5 | 14 | 95 | | 0.20 | | Bacteria |
| 16357 | 10 | | 14 | 20 | 34 | 5 | 4 | 5 | 2 | 94 | | 0.20 | | Firmicutes;Clostridia;Clostridiales |
| 7492 | 0 | | 1 | 0 | 1 | 23 | 22 | 19 | 26 | 92 | | 0.19 | | Bacteroidetes;Bacteroidetes;Bacteroidales;Prevotellaceae;Prevotella |
| 11028 | 14 | | 15 | 17 | 27 | 5 | 3 | 2 | 5 | 88 | | 0.18 | | Firmicutes;Clostridia |
| 12726 | 6 | | 5 | 5 | 9 | 14 | 20 | 18 | 10 | 87 | | 0.18 | | Firmicutes;Clostridia;Clostridiales;Ruminococcaceae;Ruminococcus |
| 19144 | 8 | | 15 | 15 | 11 | 10 | 7 | 8 | 13 | 87 | | 0.18 | | Firmicutes;Clostridia;Clostridiales;Incertae Sedis XIII;Anaerovorax |
| 10484 | 5 | | 11 | 3 | 11 | 11 | 16 | 12 | 17 | 86 | | 0.18 | | Bacteroidetes |
| 6887 | 6 | | 15 | 10 | 15 | 10 | 9 | 5 | 16 | 86 | | 0.18 | | Bacteroidetes |
| 22618 | 12 | | 22 | 23 | 29 | 0 | 0 | 0 | 0 | 86 | | 0.18 | | Bacteroidetes;Bacteroidetes;Bacteroidales |
| 10721 | 7 | | 4 | 10 | 6 | 13 | 18 | 13 | 14 | 85 | | 0.18 | | Firmicutes;Clostridia;Clostridiales;Lachnospiraceae |
| 17814 | 6 | | 15 | 18 | 15 | 11 | 7 | 6 | 5 | 83 | | 0.17 | | Firmicutes;Clostridia;Clostridiales;Ruminococcaceae |
| 15265 | 0 | | 4 | 0 | 6 | 13 | 16 | 22 | 17 | 78 | | 0.16 | | Bacteroidetes |
| 1483 | 2 | | 8 | 6 | 6 | 13 | 12 | 12 | 19 | 78 | | 0.16 | | Firmicutes;Clostridia;Clostridiales;Ruminococcaceae |
| 8149 | 6 | | 13 | 6 | 14 | 6 | 11 | 7 | 13 | 76 | | 0.16 | | Bacteroidetes;Bacteroidetes;Bacteroidales |
| 1317 | 13 | | 15 | 10 | 16 | 2 | 6 | 8 | 6 | 76 | | 0.16 | | Firmicutes;Clostridia;Clostridiales |
| 20259 | 7 | | 9 | 9 | 12 | 8 | 7 | 10 | 13 | 75 | | 0.16 | | Firmicutes;Clostridia;Clostridiales;Lachnospiraceae |
| 3446 | 8 | | 12 | 9 | 11 | 8 | 5 | 12 | 6 | 71 | | 0.15 | | Firmicutes;Clostridia;Clostridiales;Ruminococcaceae |
| 808 | 10 | | 4 | 10 | 14 | 8 | 11 | 7 | 6 | 70 | | 0.15 | | Bacteria |
| 16664 | 14 | | 17 | 20 | 19 | 0 | 0 | 0 | 0 | 70 | | 0.15 | | TM7;TM7_genera_incertae_sedis |
| 8032 | 1 | | 0 | 0 | 2 | 10 | 20 | 19 | 17 | 69 | | 0.14 | | Firmicutes;Clostridia;Clostridiales;Ruminococcaceae |
| 22850 | 10 | | 15 | 7 | 13 | 6 | 8 | 6 | 4 | 69 | | 0.14 | | Firmicutes;Clostridia;Clostridiales;Ruminococcaceae |
| 22919 | 6 | | 20 | 15 | 22 | 3 | 0 | 1 | 1 | 68 | | 0.14 | | Bacteroidetes;Bacteroidetes;Bacteroidales |
| 10824 | 8 | | 9 | 7 | 6 | 3 | 9 | 13 | 12 | 67 | | 0.14 | | Firmicutes;Clostridia |
| 14890 | 10 | | 13 | 8 | 9 | 10 | 5 | 6 | 6 | 67 | | 0.14 | | Chloroflexi;Anaerolineae |
| 7490 | 10 | | 13 | 19 | 19 | 2 | 1 | 2 | 1 | 67 | | 0.14 | | Firmicutes;Clostridia;Clostridiales;Ruminococcaceae |
| 7515 | 0 | | 0 | 2 | 1 | 10 | 20 | 12 | 20 | 65 | | 0.14 | | Bacteroidetes;Bacteroidetes;Bacteroidales;Prevotellaceae;Prevotella |
| 19713 | 0 | | 0 | 1 | 2 | 11 | 18 | 16 | 17 | 65 | | 0.14 | | Bacteroidetes;Bacteroidetes;Bacteroidales;Prevotellaceae;Prevotella |
| 19490 | 4 | | 1 | 5 | 4 | 8 | 15 | 13 | 15 | 65 | | 0.14 | | Firmicutes;Clostridia;Clostridiales;Ruminococcaceae |
| 3798 | 3 | | 13 | 8 | 7 | 7 | 10 | 9 | 8 | 65 | | 0.14 | | Firmicutes;Clostridia;Clostridiales;Ruminococcaceae;Anaerotruncus |
| 5931 | 11 | | 17 | 9 | 18 | 3 | 2 | 3 | 2 | 65 | | 0.14 | | Firmicutes;Clostridia;Clostridiales;Ruminococcaceae |
| 8956 | 2 | | 7 | 5 | 8 | 11 | 10 | 12 | 9 | 64 | | 0.13 | | Firmicutes;Clostridia;Clostridiales;Ruminococcaceae;Sporobacter |
| 6393 | 4 | | 12 | 12 | 13 | 7 | 4 | 7 | 5 | 64 | | 0.13 | | Bacteroidetes;Bacteroidetes;Bacteroidales |
| 19447 | 9 | | 17 | 8 | 29 | 0 | 0 | 0 | 1 | 64 | | 0.13 | | Bacteroidetes |
| 4126 | 10 | | 14 | 12 | 4 | 6 | 5 | 6 | 6 | 63 | | 0.13 | | Firmicutes;Clostridia;Clostridiales;Lachnospiraceae |
| 10900 | 6 | | 17 | 20 | 12 | 2 | 4 | 2 | 0 | 63 | | 0.13 | | Bacteroidetes;Bacteroidetes;Bacteroidales;Prevotellaceae |
| 3635 | 8 | | 12 | 14 | 21 | 5 | 2 | 0 | 1 | 63 | | 0.13 | | Firmicutes;Clostridia;Clostridiales |
| 20662 | 2 | | 7 | 7 | 3 | 10 | 18 | 7 | 8 | 62 | | 0.13 | | Firmicutes;Clostridia;Clostridiales;Lachnospiraceae |
| 6221 | 0 | | 5 | 3 | 3 | 7 | 13 | 13 | 15 | 59 | | 0.12 | | Firmicutes;Clostridia;Clostridiales;Ruminococcaceae;Ruminococcus |
| 16931 | 5 | | 11 | 10 | 21 | 1 | 2 | 2 | 7 | 59 | | 0.12 | | Firmicutes;Clostridia;Clostridiales;Lachnospiraceae |
| 19600 | 2 | | 2 | 0 | 0 | 14 | 20 | 8 | 12 | 58 | | 0.12 | | Bacteroidetes;Bacteroidetes;Bacteroidales |
| 10512 | 0 | | 2 | 1 | 0 | 10 | 16 | 12 | 17 | 58 | | 0.12 | | Bacteroidetes;Bacteroidetes;Bacteroidales;Prevotellaceae;Prevotella |
| 21170 | 2 | | 1 | 2 | 4 | 14 | 12 | 13 | 10 | 58 | | 0.12 | | Firmicutes;Clostridia;Clostridiales;Ruminococcaceae |
| 14261 | 1 | | 4 | 1 | 5 | 9 | 10 | 11 | 17 | 58 | | 0.12 | | Bacteria |
| 7678 | 9 | | 11 | 21 | 16 | 0 | 0 | 1 | 0 | 58 | | 0.12 | | Bacteroidetes;Bacteroidetes;Bacteroidales;Prevotellaceae |
| 6374 | 4 | | 1 | 1 | 2 | 10 | 17 | 6 | 15 | 56 | | 0.12 | | Bacteroidetes |
| 16839 | 1 | | 3 | 4 | 3 | 10 | 10 | 15 | 10 | 56 | | 0.12 | | Bacteroidetes |
| 1908 | 2 | | 7 | 7 | 8 | 9 | 10 | 7 | 6 | 56 | | 0.12 | | Firmicutes;Clostridia;Clostridiales;Ruminococcaceae |
| 5524 | 2 | | 1 | 1 | 2 | 8 | 13 | 12 | 16 | 55 | | 0.11 | | Firmicutes;Clostridia;Clostridiales;Ruminococcaceae |
| 11154 | 2 | | 3 | 4 | 4 | 9 | 11 | 11 | 11 | 55 | | 0.11 | | Bacteroidetes;Bacteroidetes;Bacteroidales |
| 1543 | 3 | | 5 | 2 | 6 | 5 | 13 | 11 | 10 | 55 | | 0.11 | | Firmicutes;Clostridia;Clostridiales;Lachnospiraceae |
| 20103 | 8 | | 10 | 8 | 15 | 1 | 5 | 3 | 5 | 55 | | 0.11 | | Firmicutes;Clostridia |
| 20288 | 2 | | 4 | 5 | 16 | 10 | 5 | 3 | 10 | 55 | | 0.11 | | Firmicutes;Clostridia;Clostridiales;Lachnospiraceae |
| 2160 | 2 | | 2 | 1 | 2 | 3 | 17 | 13 | 14 | 54 | | 0.11 | | Firmicutes;Clostridia |
| 10 | 4 | | 5 | 1 | 9 | 8 | 11 | 7 | 9 | 54 | | 0.11 | | Bacteroidetes |
| 16063 | 0 | | 1 | 1 | 1 | 13 | 13 | 12 | 12 | 53 | | 0.11 | | Bacteroidetes;Bacteroidetes;Bacteroidales;Prevotellaceae;Prevotella |
| 19751 | 4 | | 4 | 2 | 5 | 8 | 10 | 9 | 11 | 53 | | 0.11 | | Firmicutes;Clostridia;Clostridiales |
| 23646 | 4 | | 12 | 4 | 5 | 4 | 4 | 15 | 5 | 53 | | 0.11 | | Firmicutes;Clostridia;Clostridiales;Ruminococcaceae |
| 590 | 6 | | 4 | 10 | 10 | 4 | 2 | 8 | 9 | 53 | | 0.11 | | Firmicutes;Clostridia;Clostridiales;Lachnospiraceae |
| 17954 | 4 | | 11 | 5 | 14 | 7 | 4 | 5 | 3 | 53 | | 0.11 | | Firmicutes;Clostridia;Clostridiales |
| 8888 | 7 | | 11 | 9 | 16 | 2 | 3 | 0 | 5 | 53 | | 0.11 | | Bacteroidetes |
| 11899 | 1 | | 11 | 4 | 6 | 5 | 12 | 9 | 4 | 52 | | 0.11 | | Firmicutes;Clostridia;Clostridiales;Ruminococcaceae |
| 23235 | 8 | | 10 | 5 | 9 | 5 | 3 | 4 | 8 | 52 | | 0.11 | | Bacteroidetes |
| 12994 | 6 | | 25 | 9 | 12 | 0 | 0 | 0 | 0 | 52 | | 0.11 | | Bacteroidetes;Bacteroidetes;Bacteroidales;Prevotellaceae;Prevotella |
| 11198 | 5 | | 8 | 12 | 14 | 3 | 3 | 3 | 4 | 52 | | 0.11 | | Proteobacteria;Deltaproteobacteria;Desulfuromonales;Desulfuromonaceae;Desulfuromonas |
| 22233 | 1 | | 2 | 1 | 2 | 5 | 14 | 14 | 12 | 51 | | 0.11 | | Firmicutes;Clostridia;Clostridiales;Ruminococcaceae |
| 8280 | 4 | | 5 | 7 | 5 | 10 | 7 | 4 | 9 | 51 | | 0.11 | | Firmicutes;Clostridia |
| 6556 | 3 | | 10 | 12 | 5 | 6 | 5 | 6 | 4 | 51 | | 0.11 | | Firmicutes;Clostridia;Clostridiales |
| 11514 | 4 | | 8 | 6 | 9 | 5 | 5 | 9 | 5 | 51 | | 0.11 | | Bacteroidetes;Bacteroidetes;Bacteroidales |
| 19809 | 2 | | 8 | 8 | 10 | 3 | 7 | 6 | 7 | 51 | | 0.11 | | Firmicutes;Clostridia;Clostridiales;Ruminococcaceae |
| 8620 | 5 | | 7 | 8 | 16 | 2 | 3 | 4 | 6 | 51 | | 0.11 | | Firmicutes;Clostridia |
| 18682 | 1 | | 2 | 1 | 0 | 4 | 18 | 12 | 12 | 50 | | 0.10 | | Firmicutes;Clostridia;Clostridiales;Ruminococcaceae |
| 6513 | 1 | | 0 | 1 | 1 | 8 | 11 | 9 | 19 | 50 | | 0.10 | | Firmicutes;Clostridia;Clostridiales;Ruminococcaceae;Ruminococcus |
| 4846 | 8 | | 7 | 11 | 5 | 1 | 5 | 7 | 5 | 49 | | 0.10 | | Bacteroidetes;Bacteroidetes;Bacteroidales;Prevotellaceae |
| 1187 | 12 | | 2 | 10 | 9 | 2 | 10 | 1 | 3 | 49 | | 0.10 | | Bacteroidetes;Bacteroidetes;Bacteroidales |
| 4076 | 2 | | 4 | 4 | 12 | 7 | 3 | 8 | 9 | 49 | | 0.10 | | Firmicutes;Clostridia;Clostridiales;Ruminococcaceae;Ruminococcus |
| 3805 | 4 | | 13 | 11 | 9 | 0 | 1 | 5 | 5 | 48 | | 0.10 | | Bacteroidetes;Bacteroidetes;Bacteroidales;Prevotellaceae;Prevotella |
| 3955 | 7 | | 6 | 5 | 12 | 4 | 3 | 5 | 6 | 48 | | 0.10 | | Firmicutes;Clostridia;Clostridiales;Lachnospiraceae |
| 9591 | 6 | | 4 | 8 | 12 | 5 | 3 | 8 | 2 | 48 | | 0.10 | | Firmicutes;Clostridia;Clostridiales |
| 17165 | 8 | | 12 | 8 | 15 | 0 | 1 | 3 | 1 | 48 | | 0.10 | | TM7;TM7_genera_incertae_sedis |
| 13 | 1 | | 1 | 0 | 0 | 11 | 9 | 11 | 14 | 47 | | 0.10 | | Bacteroidetes;Bacteroidetes;Bacteroidales |
| 20760 | 0 | | 4 | 2 | 4 | 6 | 10 | 6 | 15 | 47 | | 0.10 | | Firmicutes;Clostridia;Clostridiales |
| 15171 | 6 | | 4 | 5 | 5 | 5 | 10 | 2 | 10 | 47 | | 0.10 | | Firmicutes;Clostridia |
| 12071 | 7 | | 12 | 4 | 22 | 0 | 0 | 1 | 0 | 46 | | 0.10 | | Bacteria |
| 17654 | 2 | | 6 | 8 | 10 | 0 | 7 | 5 | 7 | 45 | | 0.09 | | Firmicutes;Clostridia;Clostridiales |
| 13346 | 7 | | 6 | 10 | 13 | 0 | 3 | 4 | 2 | 45 | | 0.09 | | Bacteria |
| 4699 | 5 | | 9 | 5 | 14 | 3 | 4 | 3 | 1 | 44 | | 0.09 | | Firmicutes;Clostridia;Clostridiales;Ruminococcaceae;Sporobacter |
| 9875 | 3 | | 1 | 2 | 1 | 10 | 11 | 8 | 7 | 43 | | 0.09 | | Bacteria |
| 12991 | 3 | | 6 | 9 | 6 | 2 | 6 | 3 | 8 | 43 | | 0.09 | | Bacteria |
| 9202 | 0 | | 0 | 1 | 0 | 7 | 13 | 9 | 12 | 42 | | 0.09 | | Bacteroidetes |
| 10214 | 0 | | 0 | 0 | 1 | 9 | 6 | 19 | 7 | 42 | | 0.09 | | Bacteria |
| 1151 | 5 | | 2 | 5 | 5 | 2 | 5 | 11 | 7 | 42 | | 0.09 | | Firmicutes;Clostridia;Clostridiales;Lachnospiraceae |
| 12348 | 8 | | 6 | 6 | 11 | 4 | 2 | 3 | 2 | 42 | | 0.09 | | Firmicutes;Clostridia;Clostridiales |
| 3339 | 1 | | 0 | 0 | 1 | 10 | 10 | 7 | 12 | 41 | | 0.09 | | Firmicutes;Clostridia;Clostridiales;Ruminococcaceae;Ruminococcus |
| 15508 | 3 | | 4 | 4 | 5 | 4 | 8 | 3 | 10 | 41 | | 0.09 | | Firmicutes;Clostridia;Clostridiales |
| 13857 | 4 | | 8 | 7 | 9 | 5 | 4 | 4 | 0 | 41 | | 0.09 | | Firmicutes |
| 5559 | 3 | | 3 | 0 | 0 | 9 | 9 | 6 | 10 | 40 | | 0.08 | | Firmicutes;Clostridia;Clostridiales;Lachnospiraceae |
| 3786 | 1 | | 2 | 1 | 0 | 8 | 11 | 7 | 10 | 40 | | 0.08 | | Firmicutes |
| 13540 | 0 | | 1 | 1 | 3 | 7 | 12 | 7 | 9 | 40 | | 0.08 | | Firmicutes;Clostridia;Clostridiales;Lachnospiraceae |
| 22728 | 1 | | 1 | 0 | 4 | 7 | 10 | 10 | 7 | 40 | | 0.08 | | Firmicutes;Clostridia;Clostridiales;Veillonellaceae |
| 5581 | 3 | | 2 | 3 | 7 | 4 | 7 | 11 | 3 | 40 | | 0.08 | | Bacteria |
| 15538 | 1 | | 7 | 3 | 8 | 1 | 7 | 4 | 9 | 40 | | 0.08 | | Firmicutes;Clostridia;Clostridiales |
| 18461 | 0 | | 0 | 0 | 0 | 10 | 10 | 7 | 12 | 39 | | 0.08 | | Bacteroidetes;Bacteroidetes;Bacteroidales |
| 5555 | 0 | | 0 | 0 | 0 | 4 | 12 | 11 | 12 | 39 | | 0.08 | | Bacteroidetes;Bacteroidetes;Bacteroidales;Prevotellaceae |
| 18728 | 5 | | 3 | 3 | 0 | 4 | 10 | 10 | 4 | 39 | | 0.08 | | Bacteroidetes;Bacteroidetes;Bacteroidales;Prevotellaceae |
| 634 | 0 | | 0 | 0 | 2 | 4 | 8 | 12 | 13 | 39 | | 0.08 | | Firmicutes;Clostridia;Clostridiales;Ruminococcaceae;Ruminococcus |
| 15 | 3 | | 6 | 6 | 2 | 2 | 5 | 7 | 8 | 39 | | 0.08 | | Bacteroidetes;Bacteroidetes;Bacteroidales |
| 23683 | 0 | | 1 | 2 | 4 | 8 | 9 | 10 | 5 | 39 | | 0.08 | | Firmicutes;Clostridia;Clostridiales;Lachnospiraceae;Pseudobutyrivibrio |
| 7816 | 2 | | 6 | 2 | 5 | 7 | 7 | 6 | 4 | 39 | | 0.08 | | Firmicutes;Clostridia;Clostridiales;Lachnospiraceae |
| 9936 | 3 | | 8 | 6 | 8 | 1 | 6 | 2 | 5 | 39 | | 0.08 | | Bacteria |
| 19372 | 1 | | 3 | 2 | 0 | 5 | 9 | 9 | 9 | 38 | | 0.08 | | Bacteroidetes;Bacteroidetes;Bacteroidales;Prevotellaceae |
| 17291 | 0 | | 3 | 1 | 1 | 12 | 9 | 5 | 7 | 38 | | 0.08 | | Bacteroidetes |
| 3640 | 2 | | 10 | 8 | 3 | 2 | 4 | 5 | 4 | 38 | | 0.08 | | Firmicutes;Clostridia;Clostridiales;Lachnospiraceae |
| 5910 | 3 | | 4 | 2 | 4 | 4 | 7 | 7 | 7 | 38 | | 0.08 | | TM7;TM7_genera_incertae_sedis |
| 18412 | 1 | | 0 | 0 | 1 | 9 | 8 | 7 | 11 | 37 | | 0.08 | | Bacteroidetes;Bacteroidetes;Bacteroidales;Prevotellaceae;Prevotella |
| 17821 | 3 | | 5 | 1 | 4 | 4 | 3 | 4 | 13 | 37 | | 0.08 | | Planctomycetes;Planctomycetacia;Planctomycetales;Planctomycetaceae |
| 19496 | 3 | | 3 | 5 | 9 | 2 | 5 | 4 | 6 | 37 | | 0.08 | | Firmicutes;Clostridia |
| 20963 | 2 | | 9 | 3 | 4 | 1 | 7 | 6 | 4 | 36 | | 0.08 | | Bacteria |
| 2325 | 5 | | 6 | 4 | 7 | 3 | 1 | 5 | 5 | 36 | | 0.08 | | Firmicutes;Clostridia;Clostridiales |
| 10196 | 5 | | 4 | 6 | 11 | 1 | 0 | 6 | 3 | 36 | | 0.08 | | Firmicutes;Clostridia;Clostridiales;Lachnospiraceae |
| 16016 | 6 | | 7 | 6 | 12 | 3 | 0 | 1 | 1 | 36 | | 0.08 | | Firmicutes;Clostridia;Clostridiales;Ruminococcaceae |
| 8909 | 1 | | 0 | 0 | 0 | 5 | 14 | 4 | 11 | 35 | | 0.07 | | Bacteroidetes;Bacteroidetes;Bacteroidales |
| 15097 | 0 | | 1 | 1 | 2 | 8 | 4 | 6 | 13 | 35 | | 0.07 | | Firmicutes;Clostridia;Clostridiales;Ruminococcaceae |
| 2318 | 0 | | 4 | 0 | 3 | 5 | 6 | 11 | 6 | 35 | | 0.07 | | Bacteria |
| 2617 | 2 | | 3 | 5 | 3 | 8 | 3 | 1 | 10 | 35 | | 0.07 | | Bacteroidetes |
| 1741 | 3 | | 6 | 7 | 6 | 4 | 3 | 5 | 1 | 35 | | 0.07 | | Firmicutes;Clostridia;Clostridiales;Ruminococcaceae |
| 2372 | 4 | | 9 | 7 | 6 | 2 | 3 | 3 | 1 | 35 | | 0.07 | | Bacteroidetes;Bacteroidetes;Bacteroidales |
| 7135 | 1 | | 2 | 4 | 7 | 4 | 6 | 6 | 5 | 35 | | 0.07 | | Firmicutes;Clostridia;Clostridiales;Ruminococcaceae |
| 15922 | 4 | | 13 | 8 | 10 | 0 | 0 | 0 | 0 | 35 | | 0.07 | | Firmicutes;Clostridia;Clostridiales;Lachnospiraceae |
| 13971 | 0 | | 0 | 0 | 0 | 4 | 15 | 9 | 6 | 34 | | 0.07 | | Bacteroidetes;Bacteroidetes;Bacteroidales |
| 618 | 0 | | 6 | 1 | 0 | 3 | 9 | 8 | 7 | 34 | | 0.07 | | Firmicutes;Clostridia;Clostridiales |
| 4362 | 0 | | 1 | 1 | 2 | 10 | 10 | 6 | 4 | 34 | | 0.07 | | Firmicutes;Clostridia;Clostridiales;Ruminococcaceae;Ruminococcus |
| 16210 | 2 | | 3 | 2 | 3 | 7 | 4 | 5 | 8 | 34 | | 0.07 | | Firmicutes;Clostridia;Clostridiales;Lachnospiraceae |
| 22588 | 2 | | 1 | 4 | 3 | 8 | 4 | 4 | 8 | 34 | | 0.07 | | Bacteroidetes;Bacteroidetes;Bacteroidales |
| 15845 | 4 | | 6 | 6 | 3 | 2 | 7 | 3 | 3 | 34 | | 0.07 | | Firmicutes;Clostridia;Clostridiales |
| 20931 | 5 | | 8 | 5 | 9 | 3 | 0 | 2 | 2 | 34 | | 0.07 | | Firmicutes;Clostridia |
| 6506 | 0 | | 0 | 0 | 0 | 7 | 12 | 9 | 5 | 33 | | 0.07 | | Bacteroidetes;Bacteroidetes;Bacteroidales;Prevotellaceae;Prevotella |
| 9556 | 2 | | 2 | 1 | 3 | 7 | 5 | 12 | 1 | 33 | | 0.07 | | Firmicutes;Clostridia;Clostridiales;Lachnospiraceae;Coprococcus |
| 3579 | 1 | | 3 | 1 | 5 | 2 | 6 | 9 | 6 | 33 | | 0.07 | | Bacteria |
| 14331 | 2 | | 9 | 2 | 7 | 2 | 6 | 3 | 2 | 33 | | 0.07 | | Firmicutes;Clostridia;Clostridiales;Ruminococcaceae |
| 2930 | 2 | | 6 | 10 | 8 | 1 | 0 | 2 | 4 | 33 | | 0.07 | | Firmicutes;Clostridia;Clostridiales;Lachnospiraceae |
| 14023 | 3 | | 2 | 2 | 2 | 4 | 4 | 4 | 11 | 32 | | 0.07 | | Chloroflexi;Anaerolineae |
| 5084 | 0 | | 0 | 4 | 3 | 5 | 7 | 5 | 8 | 32 | | 0.07 | | Firmicutes |
| 15504 | 0 | | 0 | 0 | 5 | 1 | 10 | 7 | 9 | 32 | | 0.07 | | Firmicutes;Clostridia;Clostridiales;Lachnospiraceae |
| 2610 | 2 | | 4 | 3 | 5 | 2 | 7 | 5 | 4 | 32 | | 0.07 | | Firmicutes;Clostridia;Clostridiales;Lachnospiraceae |
| 12394 | 7 | | 3 | 2 | 6 | 1 | 5 | 2 | 6 | 32 | | 0.07 | | Firmicutes;Clostridia;Clostridiales;Ruminococcaceae;Ruminococcus |
| 19446 | 2 | | 4 | 2 | 6 | 1 | 5 | 4 | 8 | 32 | | 0.07 | | Bacteria |
| 913 | 6 | | 7 | 4 | 7 | 3 | 1 | 3 | 1 | 32 | | 0.07 | | Chloroflexi;Anaerolineae |
| 20623 | 1 | | 9 | 10 | 12 | 0 | 0 | 0 | 0 | 32 | | 0.07 | | Bacteroidetes |
| 17640 | 4 | | 2 | 1 | 4 | 1 | 7 | 5 | 7 | 31 | | 0.06 | | Firmicutes;Clostridia;Clostridiales;Lachnospiraceae;Lachnospiraceae Incertae Sedis |
| 10795 | 6 | | 6 | 4 | 15 | 0 | 0 | 0 | 0 | 31 | | 0.06 | | Bacteroidetes;Bacteroidetes;Bacteroidales;Prevotellaceae;Hallella |
| 14543 | 0 | | 0 | 0 | 0 | 3 | 10 | 7 | 10 | 30 | | 0.06 | | Bacteroidetes;Bacteroidetes;Bacteroidales;Prevotellaceae;Prevotella |
| 3808 | 0 | | 1 | 0 | 1 | 3 | 7 | 10 | 8 | 30 | | 0.06 | | Firmicutes;Clostridia;Clostridiales;Lachnospiraceae |
| 8319 | 3 | | 3 | 1 | 5 | 2 | 4 | 6 | 6 | 30 | | 0.06 | | Firmicutes;Clostridia;Clostridiales;Lachnospiraceae |
| 4179 | 5 | | 7 | 1 | 6 | 1 | 4 | 2 | 4 | 30 | | 0.06 | | Firmicutes;Clostridia |
| 7451 | 0 | | 5 | 4 | 6 | 5 | 2 | 2 | 6 | 30 | | 0.06 | | TM7;TM7_genera_incertae_sedis |
| 10774 | 3 | | 5 | 9 | 8 | 0 | 1 | 2 | 2 | 30 | | 0.06 | | Firmicutes;Clostridia;Clostridiales;Veillonellaceae;Succiniclasticum |
| 6821 | 9 | | 5 | 4 | 9 | 0 | 1 | 1 | 1 | 30 | | 0.06 | | Bacteroidetes |
| 17605 | 5 | | 8 | 4 | 11 | 0 | 0 | 0 | 2 | 30 | | 0.06 | | Bacteroidetes |
| 18196 | 0 | | 0 | 0 | 0 | 2 | 9 | 9 | 9 | 29 | | 0.06 | | Bacteroidetes;Bacteroidetes;Bacteroidales;Prevotellaceae |
| 924 | 0 | | 1 | 1 | 0 | 7 | 4 | 6 | 10 | 29 | | 0.06 | | Bacteria |
| 3840 | 3 | | 2 | 3 | 0 | 2 | 6 | 7 | 6 | 29 | | 0.06 | | Bacteroidetes;Bacteroidetes;Bacteroidales;Prevotellaceae |
| 18556 | 0 | | 0 | 0 | 1 | 1 | 6 | 9 | 12 | 29 | | 0.06 | | Bacteroidetes;Bacteroidetes;Bacteroidales |
| 1700 | 1 | | 0 | 0 | 3 | 2 | 8 | 6 | 9 | 29 | | 0.06 | | Bacteroidetes;Bacteroidetes;Bacteroidales |
| 138 | 1 | | 1 | 5 | 3 | 5 | 7 | 3 | 4 | 29 | | 0.06 | | Firmicutes;Clostridia;Clostridiales |
| 19448 | 1 | | 4 | 1 | 4 | 2 | 7 | 7 | 3 | 29 | | 0.06 | | Firmicutes;Clostridia;Clostridiales;Lachnospiraceae |
| 14645 | 3 | | 2 | 2 | 5 | 4 | 7 | 1 | 5 | 29 | | 0.06 | | Firmicutes;Clostridia;Clostridiales;Lachnospiraceae;Lachnospiraceae Incertae Sedis |
| 17882 | 2 | | 0 | 2 | 6 | 6 | 7 | 4 | 2 | 29 | | 0.06 | | Bacteroidetes;Bacteroidetes;Bacteroidales |
| 11137 | 4 | | 17 | 1 | 7 | 0 | 0 | 0 | 0 | 29 | | 0.06 | | Bacteroidetes;Bacteroidetes;Bacteroidales |
| 1621 | 4 | | 6 | 8 | 11 | 0 | 0 | 0 | 0 | 29 | | 0.06 | | Bacteroidetes;Bacteroidetes;Bacteroidales;Prevotellaceae |
| 21630 | 1 | | 0 | 0 | 0 | 8 | 4 | 7 | 8 | 28 | | 0.06 | | Firmicutes;Clostridia;Clostridiales;Ruminococcaceae |
| 2584 | 2 | | 2 | 0 | 2 | 5 | 8 | 2 | 7 | 28 | | 0.06 | | Firmicutes;Clostridia;Clostridiales;Lachnospiraceae |
| 13789 | 2 | | 7 | 5 | 3 | 5 | 1 | 4 | 1 | 28 | | 0.06 | | Firmicutes;Clostridia;Clostridiales;Lachnospiraceae |
| 14178 | 3 | | 3 | 5 | 4 | 4 | 2 | 5 | 2 | 28 | | 0.06 | | Firmicutes;Clostridia;Clostridiales;Lachnospiraceae |
| 14560 | 6 | | 6 | 7 | 4 | 1 | 0 | 2 | 2 | 28 | | 0.06 | | Firmicutes;Clostridia;Clostridiales;Ruminococcaceae |
| 15675 | 1 | | 6 | 1 | 5 | 3 | 5 | 2 | 5 | 28 | | 0.06 | | Firmicutes;Clostridia;Clostridiales;Ruminococcaceae |
| 6837 | 2 | | 3 | 2 | 6 | 2 | 4 | 6 | 3 | 28 | | 0.06 | | Firmicutes |
| 6347 | 8 | | 6 | 4 | 10 | 0 | 0 | 0 | 0 | 28 | | 0.06 | | Bacteroidetes;Bacteroidetes;Bacteroidales;Prevotellaceae |
| 171 | 0 | | 0 | 0 | 0 | 5 | 9 | 6 | 7 | 27 | | 0.06 | | Bacteroidetes;Bacteroidetes;Bacteroidales;Prevotellaceae;Prevotella |
| 3079 | 2 | | 3 | 0 | 1 | 2 | 5 | 9 | 5 | 27 | | 0.06 | | Bacteria |
| 7238 | 1 | | 4 | 2 | 1 | 6 | 3 | 5 | 5 | 27 | | 0.06 | | Firmicutes;Clostridia;Clostridiales;Lachnospiraceae |
| 2447 | 3 | | 8 | 1 | 3 | 3 | 3 | 3 | 3 | 27 | | 0.06 | | Bacteroidetes;Bacteroidetes;Bacteroidales;Prevotellaceae |
| 13389 | 2 | | 5 | 8 | 3 | 3 | 5 | 1 | 0 | 27 | | 0.06 | | Firmicutes;Clostridia;Clostridiales |
| 595 | 2 | | 14 | 6 | 5 | 0 | 0 | 0 | 0 | 27 | | 0.06 | | Bacteroidetes;Bacteroidetes;Bacteroidales;Prevotellaceae |
| 8063 | 0 | | 7 | 4 | 6 | 3 | 1 | 4 | 2 | 27 | | 0.06 | | Firmicutes;Clostridia;Clostridiales |
| 1404 | 3 | | 6 | 5 | 6 | 2 | 3 | 0 | 2 | 27 | | 0.06 | | Bacteroidetes;Bacteroidetes;Bacteroidales |
| 9804 | 5 | | 6 | 3 | 7 | 0 | 2 | 0 | 4 | 27 | | 0.06 | | Firmicutes;Clostridia;Clostridiales;Ruminococcaceae |
| 3967 | 0 | | 0 | 0 | 0 | 6 | 5 | 5 | 10 | 26 | | 0.05 | | Bacteroidetes;Bacteroidetes;Bacteroidales;Prevotellaceae;Prevotella |
| 1932 | 2 | | 0 | 0 | 0 | 4 | 12 | 2 | 6 | 26 | | 0.05 | | Firmicutes;Clostridia;Clostridiales;Ruminococcaceae |
| 16110 | 1 | | 1 | 0 | 2 | 3 | 8 | 5 | 6 | 26 | | 0.05 | | Bacteroidetes;Bacteroidetes;Bacteroidales |
| 15982 | 1 | | 0 | 1 | 2 | 6 | 7 | 6 | 3 | 26 | | 0.05 | | Bacteria |
| 17381 | 2 | | 2 | 2 | 2 | 6 | 3 | 1 | 8 | 26 | | 0.05 | | Firmicutes;Clostridia;Clostridiales;Ruminococcaceae;Ruminococcus |
| 4615 | 0 | | 3 | 3 | 3 | 3 | 5 | 6 | 3 | 26 | | 0.05 | | Firmicutes;Clostridia;Clostridiales;Lachnospiraceae |
| 5902 | 3 | | 5 | 2 | 4 | 1 | 6 | 1 | 4 | 26 | | 0.05 | | Firmicutes;Clostridia;Clostridiales |
| 3216 | 1 | | 5 | 5 | 7 | 0 | 2 | 2 | 4 | 26 | | 0.05 | | Firmicutes;Clostridia;Clostridiales;Lachnospiraceae |
| 4797 | 2 | | 1 | 1 | 8 | 5 | 2 | 5 | 2 | 26 | | 0.05 | | Firmicutes;Clostridia;Clostridiales;Lachnospiraceae |
| 4527 | 1 | | 0 | 1 | 0 | 5 | 9 | 5 | 4 | 25 | | 0.05 | | Bacteroidetes;Bacteroidetes;Bacteroidales |
| 13038 | 2 | | 7 | 6 | 1 | 1 | 2 | 2 | 4 | 25 | | 0.05 | | Firmicutes;Clostridia;Clostridiales;Lachnospiraceae |
| 22727 | 0 | | 2 | 1 | 2 | 1 | 4 | 9 | 6 | 25 | | 0.05 | | Firmicutes;Clostridia;Clostridiales;Lachnospiraceae |
| 17391 | 3 | | 3 | 1 | 2 | 1 | 6 | 1 | 8 | 25 | | 0.05 | | Bacteria |
| 3337 | 0 | | 1 | 2 | 2 | 3 | 6 | 5 | 6 | 25 | | 0.05 | | Bacteroidetes;Bacteroidetes;Bacteroidales |
| 14006 | 6 | | 3 | 2 | 2 | 2 | 4 | 2 | 4 | 25 | | 0.05 | | Firmicutes;Clostridia;Clostridiales |
| 4418 | 0 | | 4 | 4 | 2 | 2 | 3 | 5 | 5 | 25 | | 0.05 | | Bacteroidetes |
| 5621 | 0 | | 1 | 2 | 3 | 3 | 8 | 3 | 5 | 25 | | 0.05 | | Bacteroidetes |
| 21558 | 3 | | 0 | 3 | 5 | 3 | 5 | 2 | 4 | 25 | | 0.05 | | Firmicutes;Clostridia;Clostridiales;Ruminococcaceae |
| 17064 | 4 | | 5 | 3 | 6 | 3 | 0 | 2 | 2 | 25 | | 0.05 | | Firmicutes;Clostridia;Clostridiales;Lachnospiraceae |
| 7925 | 3 | | 3 | 3 | 9 | 1 | 3 | 1 | 2 | 25 | | 0.05 | | Firmicutes;Clostridia;Clostridiales |
| 12257 | 4 | | 6 | 6 | 9 | 0 | 0 | 0 | 0 | 25 | | 0.05 | | Bacteroidetes;Bacteroidetes;Bacteroidales;Prevotellaceae |
| 2927 | 2 | | 7 | 6 | 10 | 0 | 0 | 0 | 0 | 25 | | 0.05 | | Bacteroidetes;Bacteroidetes;Bacteroidales;Prevotellaceae |
| 1019 | 0 | | 2 | 1 | 11 | 2 | 4 | 3 | 2 | 25 | | 0.05 | | Firmicutes;Clostridia;Clostridiales;Ruminococcaceae |
| 4721 | 0 | | 2 | 1 | 12 | 2 | 4 | 4 | 0 | 25 | | 0.05 | | Firmicutes;Clostridia;Clostridiales;Lachnospiraceae |
| 19814 | 0 | | 0 | 0 | 0 | 5 | 10 | 5 | 4 | 24 | | 0.05 | | Bacteroidetes;Bacteroidetes;Bacteroidales;Prevotellaceae;Prevotella |
| 4599 | 0 | | 2 | 0 | 0 | 3 | 8 | 7 | 4 | 24 | | 0.05 | | Bacteroidetes |
| 10728 | 1 | | 2 | 0 | 0 | 5 | 3 | 6 | 7 | 24 | | 0.05 | | Firmicutes;Clostridia;Clostridiales;Ruminococcaceae |
| 17333 | 0 | | 2 | 2 | 0 | 5 | 5 | 5 | 5 | 24 | | 0.05 | | Bacteria |
| 7300 | 1 | | 2 | 0 | 1 | 1 | 13 | 2 | 4 | 24 | | 0.05 | | Bacteroidetes;Bacteroidetes;Bacteroidales;Prevotellaceae |
| 6696 | 2 | | 4 | 0 | 1 | 5 | 8 | 2 | 2 | 24 | | 0.05 | | Firmicutes;Clostridia;Clostridiales;Ruminococcaceae |
| 14346 | 0 | | 0 | 0 | 2 | 3 | 6 | 6 | 7 | 24 | | 0.05 | | Bacteroidetes;Bacteroidetes;Bacteroidales |
| 9799 | 2 | | 3 | 1 | 2 | 4 | 2 | 4 | 6 | 24 | | 0.05 | | Firmicutes;Clostridia;Clostridiales;Incertae Sedis XIII;Anaerovorax |
| 11277 | 3 | | 2 | 3 | 3 | 3 | 4 | 4 | 2 | 24 | | 0.05 | | Proteobacteria;Deltaproteobacteria;Desulfuromonales;Desulfuromonaceae;Pelobacter |
| 7754 | 6 | | 5 | 4 | 6 | 0 | 0 | 1 | 2 | 24 | | 0.05 | | Firmicutes;Clostridia;Clostridiales |
| 8005 | 3 | | 7 | 8 | 6 | 0 | 0 | 0 | 0 | 24 | | 0.05 | | Firmicutes;Clostridia;Clostridiales;Lachnospiraceae;Lachnospiraceae Incertae Sedis |
| 12390 | 2 | | 6 | 7 | 9 | 0 | 0 | 0 | 0 | 24 | | 0.05 | | Bacteroidetes |
| 4358 | 0 | | 0 | 1 | 0 | 5 | 7 | 3 | 7 | 23 | | 0.05 | | TM7;TM7_genera_incertae_sedis |
| 4104 | 0 | | 3 | 1 | 0 | 1 | 8 | 8 | 2 | 23 | | 0.05 | | Bacteroidetes;Bacteroidetes;Bacteroidales |
| 6235 | 1 | | 1 | 2 | 0 | 4 | 8 | 5 | 2 | 23 | | 0.05 | | Firmicutes;Clostridia;Clostridiales |
| 21611 | 0 | | 3 | 0 | 2 | 5 | 2 | 5 | 6 | 23 | | 0.05 | | Firmicutes;Clostridia;Clostridiales |
| 14227 | 2 | | 3 | 1 | 2 | 1 | 2 | 8 | 4 | 23 | | 0.05 | | Planctomycetes;Planctomycetacia;Planctomycetales;Planctomycetaceae |
| 13051 | 0 | | 3 | 2 | 2 | 4 | 2 | 5 | 5 | 23 | | 0.05 | | Firmicutes;Clostridia;Clostridiales;Ruminococcaceae;Ruminococcaceae Incertae Sedis |
| 4099 | 1 | | 1 | 3 | 2 | 1 | 5 | 3 | 7 | 23 | | 0.05 | | Bacteria |
| 14815 | 2 | | 1 | 3 | 3 | 2 | 2 | 4 | 6 | 23 | | 0.05 | | Firmicutes;Clostridia;Clostridiales;Lachnospiraceae;Lachnospiraceae Incertae Sedis |
| 17719 | 1 | | 4 | 5 | 3 | 1 | 4 | 2 | 3 | 23 | | 0.05 | | Bacteria |
| 13603 | 2 | | 5 | 7 | 3 | 0 | 3 | 2 | 1 | 23 | | 0.05 | | Bacteria |
| 21173 | 1 | | 3 | 2 | 5 | 1 | 3 | 4 | 4 | 23 | | 0.05 | | Firmicutes;Clostridia;Clostridiales;Lachnospiraceae |
| 19295 | 5 | | 4 | 8 | 6 | 0 | 0 | 0 | 0 | 23 | | 0.05 | | Bacteroidetes;Bacteroidetes;Bacteroidales;Prevotellaceae |
| 22181 | 1 | | 7 | 9 | 6 | 0 | 0 | 0 | 0 | 23 | | 0.05 | | Bacteroidetes |
| 12513 | 1 | | 2 | 5 | 7 | 1 | 4 | 2 | 1 | 23 | | 0.05 | | Firmicutes;Clostridia;Clostridiales;Lachnospiraceae |
| 8832 | 0 | | 4 | 1 | 8 | 4 | 2 | 2 | 2 | 23 | | 0.05 | | Firmicutes;Clostridia;Clostridiales;Lachnospiraceae |
| 18725 | 3 | | 6 | 5 | 9 | 0 | 0 | 0 | 0 | 23 | | 0.05 | | Bacteroidetes;Bacteroidetes;Bacteroidales |
| 3443 | 0 | | 0 | 0 | 0 | 4 | 5 | 5 | 8 | 22 | | 0.05 | | Bacteroidetes;Bacteroidetes;Bacteroidales;Prevotellaceae;Prevotella |
| 2973 | 0 | | 0 | 0 | 0 | 7 | 4 | 2 | 9 | 22 | | 0.05 | | Bacteroidetes;Bacteroidetes;Bacteroidales;Prevotellaceae;Prevotella |
| 14496 | 0 | | 0 | 0 | 0 | 3 | 6 | 4 | 9 | 22 | | 0.05 | | Bacteroidetes;Bacteroidetes;Bacteroidales |
| 1205 | 0 | | 0 | 2 | 0 | 0 | 6 | 6 | 8 | 22 | | 0.05 | | Bacteroidetes;Bacteroidetes;Bacteroidales;Prevotellaceae;Prevotella |
| 11013 | 1 | | 5 | 0 | 1 | 1 | 5 | 6 | 3 | 22 | | 0.05 | | Bacteroidetes;Bacteroidetes;Bacteroidales |
| 5591 | 0 | | 1 | 1 | 1 | 2 | 9 | 2 | 6 | 22 | | 0.05 | | Bacteroidetes;Sphingobacteria;Sphingobacteriales |
| 19403 | 4 | | 7 | 9 | 1 | 0 | 0 | 1 | 0 | 22 | | 0.05 | | Firmicutes;Clostridia;Clostridiales;Ruminococcaceae |
| 8607 | 0 | | 4 | 1 | 2 | 1 | 2 | 10 | 2 | 22 | | 0.05 | | Bacteria |
| 22049 | 1 | | 2 | 4 | 2 | 2 | 1 | 4 | 6 | 22 | | 0.05 | | Firmicutes;Clostridia;Clostridiales;Incertae Sedis XIII;Anaerovorax |
| 18698 | 0 | | 2 | 3 | 3 | 2 | 2 | 3 | 7 | 22 | | 0.05 | | Bacteria |
| 19988 | 2 | | 2 | 3 | 3 | 4 | 2 | 3 | 3 | 22 | | 0.05 | | Chloroflexi;Anaerolineae |
| 18763 | 2 | | 6 | 10 | 3 | 0 | 0 | 0 | 1 | 22 | | 0.05 | | Bacteroidetes;Bacteroidetes;Bacteroidales;Prevotellaceae |
| 21364 | 5 | | 6 | 3 | 4 | 0 | 0 | 2 | 2 | 22 | | 0.05 | | Firmicutes;Clostridia |
| 12252 | 2 | | 6 | 4 | 4 | 2 | 3 | 1 | 0 | 22 | | 0.05 | | Firmicutes;Clostridia;Clostridiales |
| 23621 | 2 | | 3 | 5 | 4 | 1 | 4 | 0 | 3 | 22 | | 0.05 | | Firmicutes;Clostridia;Clostridiales |
| 20249 | 1 | | 1 | 2 | 5 | 2 | 3 | 4 | 4 | 22 | | 0.05 | | Bacteria |
| 434 | 7 | | 6 | 1 | 6 | 0 | 0 | 0 | 2 | 22 | | 0.05 | | Bacteria |
| 3562 | 4 | | 5 | 1 | 7 | 1 | 3 | 1 | 0 | 22 | | 0.05 | | Firmicutes;Clostridia;Clostridiales |
| 9254 | 3 | | 4 | 5 | 7 | 3 | 0 | 0 | 0 | 22 | | 0.05 | | Bacteroidetes;Bacteroidetes;Bacteroidales |
| 6018 | 0 | | 0 | 0 | 0 | 6 | 6 | 5 | 4 | 21 | | 0.04 | | Bacteroidetes;Bacteroidetes;Bacteroidales;Prevotellaceae |
| 21803 | 0 | | 0 | 0 | 0 | 4 | 4 | 7 | 6 | 21 | | 0.04 | | Bacteroidetes;Bacteroidetes;Bacteroidales;Prevotellaceae |
| 10065 | 0 | | 0 | 0 | 0 | 2 | 4 | 8 | 7 | 21 | | 0.04 | | Bacteroidetes;Bacteroidetes;Bacteroidales;Prevotellaceae |
| 11481 | 0 | | 1 | 0 | 0 | 4 | 7 | 5 | 4 | 21 | | 0.04 | | Bacteroidetes;Bacteroidetes;Bacteroidales |
| 17477 | 0 | | 1 | 0 | 0 | 2 | 8 | 4 | 6 | 21 | | 0.04 | | Firmicutes;Clostridia;Clostridiales |
| 23254 | 0 | | 6 | 4 | 0 | 3 | 2 | 5 | 1 | 21 | | 0.04 | | Firmicutes;Clostridia;Clostridiales;Ruminococcaceae |
| 4765 | 0 | | 2 | 0 | 3 | 4 | 1 | 6 | 5 | 21 | | 0.04 | | Firmicutes;Clostridia |
| 15121 | 3 | | 6 | 4 | 3 | 2 | 1 | 0 | 2 | 21 | | 0.04 | | Firmicutes;Clostridia;Clostridiales |
| 1635 | 2 | | 3 | 2 | 4 | 1 | 1 | 4 | 4 | 21 | | 0.04 | | Firmicutes;Clostridia;Clostridiales;Ruminococcaceae |
| 135 | 1 | | 6 | 4 | 4 | 1 | 2 | 2 | 1 | 21 | | 0.04 | | Bacteroidetes |
| 9196 | 0 | | 10 | 0 | 5 | 0 | 1 | 2 | 3 | 21 | | 0.04 | | Bacteroidetes;Bacteroidetes;Bacteroidales |
| 7780 | 8 | | 2 | 1 | 5 | 1 | 2 | 0 | 2 | 21 | | 0.04 | | Firmicutes;Clostridia;Clostridiales;Lachnospiraceae |
| 1196 | 0 | | 8 | 4 | 5 | 0 | 0 | 3 | 1 | 21 | | 0.04 | | Firmicutes;Clostridia;Clostridiales;Veillonellaceae;Succiniclasticum |
| 8583 | 3 | | 8 | 3 | 7 | 0 | 0 | 0 | 0 | 21 | | 0.04 | | Bacteroidetes;Bacteroidetes;Bacteroidales;Prevotellaceae;Hallella |
| 9727 | 6 | | 4 | 4 | 7 | 0 | 0 | 0 | 0 | 21 | | 0.04 | | Firmicutes;Clostridia;Clostridiales;Ruminococcaceae |
| 2321 | 1 | | 2 | 0 | 0 | 3 | 8 | 3 | 3 | 20 | | 0.04 | | Firmicutes;Clostridia;Clostridiales;Ruminococcaceae |
| 1685 | 1 | | 6 | 0 | 0 | 0 | 1 | 9 | 3 | 20 | | 0.04 | | Bacteroidetes;Bacteroidetes;Bacteroidales |
| 18990 | 0 | | 2 | 1 | 0 | 4 | 4 | 4 | 5 | 20 | | 0.04 | | Bacteroidetes;Bacteroidetes;Bacteroidales;Prevotellaceae |
| 15657 | 0 | | 0 | 1 | 1 | 3 | 2 | 4 | 9 | 20 | | 0.04 | | Bacteroidetes;Bacteroidetes;Bacteroidales |
| 17158 | 3 | | 7 | 8 | 2 | 0 | 0 | 0 | 0 | 20 | | 0.04 | | Firmicutes;Clostridia;Clostridiales;Lachnospiraceae |
| 16961 | 0 | | 2 | 3 | 3 | 4 | 3 | 4 | 1 | 20 | | 0.04 | | Firmicutes;Clostridia;Clostridiales;Ruminococcaceae |
| 19203 | 0 | | 3 | 3 | 3 | 4 | 4 | 1 | 2 | 20 | | 0.04 | | Bacteroidetes;Bacteroidetes;Bacteroidales;Prevotellaceae;Prevotella |
| 2796 | 2 | | 3 | 4 | 3 | 2 | 1 | 1 | 4 | 20 | | 0.04 | | Bacteroidetes;Bacteroidetes;Bacteroidales |
| 2989 | 8 | | 4 | 1 | 5 | 0 | 0 | 0 | 2 | 20 | | 0.04 | | Firmicutes;Clostridia;Clostridiales;Ruminococcaceae;Ruminococcus |
| 14275 | 0 | | 5 | 5 | 5 | 0 | 4 | 1 | 0 | 20 | | 0.04 | | Bacteria |
| 20325 | 0 | | 7 | 5 | 5 | 1 | 1 | 1 | 0 | 20 | | 0.04 | | Bacteroidetes;Bacteroidetes;Bacteroidales;Prevotellaceae |
| 15112 | 1 | | 2 | 2 | 6 | 1 | 2 | 4 | 2 | 20 | | 0.04 | | Bacteria |
| 10642 | 3 | | 2 | 6 | 6 | 0 | 1 | 1 | 1 | 20 | | 0.04 | | Bacteroidetes |
| 22151 | 4 | | 4 | 3 | 9 | 0 | 0 | 0 | 0 | 20 | | 0.04 | | Bacteroidetes;Bacteroidetes;Bacteroidales;Prevotellaceae |
| 23293 | 0 | | 0 | 0 | 0 | 2 | 5 | 6 | 6 | 19 | | 0.04 | | Bacteroidetes;Bacteroidetes;Bacteroidales;Prevotellaceae;Prevotella |
| 15379 | 0 | | 0 | 0 | 0 | 4 | 5 | 2 | 8 | 19 | | 0.04 | | Bacteroidetes;Bacteroidetes;Bacteroidales;Prevotellaceae;Prevotella |
| 9854 | 0 | | 1 | 2 | 0 | 2 | 5 | 6 | 3 | 19 | | 0.04 | | Bacteroidetes;Bacteroidetes;Bacteroidales |
| 189 | 0 | | 2 | 0 | 1 | 2 | 6 | 3 | 5 | 19 | | 0.04 | | Firmicutes;Clostridia;Clostridiales;Ruminococcaceae |
| 11696 | 3 | | 3 | 0 | 1 | 2 | 2 | 2 | 6 | 19 | | 0.04 | | Firmicutes;Clostridia;Clostridiales;Ruminococcaceae |
| 19989 | 0 | | 0 | 1 | 1 | 0 | 4 | 4 | 9 | 19 | | 0.04 | | Firmicutes |
| 19226 | 1 | | 1 | 1 | 1 | 5 | 4 | 2 | 4 | 19 | | 0.04 | | Firmicutes;Clostridia;Clostridiales |
| 3418 | 2 | | 2 | 1 | 1 | 3 | 2 | 5 | 3 | 19 | | 0.04 | | Firmicutes;Clostridia;Clostridiales;Lachnospiraceae |
| 6644 | 0 | | 4 | 3 | 1 | 2 | 3 | 1 | 5 | 19 | | 0.04 | | Bacteroidetes;Bacteroidetes;Bacteroidales;Prevotellaceae;Prevotella |
| 2436 | 0 | | 0 | 0 | 2 | 3 | 2 | 4 | 8 | 19 | | 0.04 | | Firmicutes;Clostridia;Clostridiales;Ruminococcaceae;Ruminococcus |
| 4508 | 1 | | 1 | 2 | 2 | 3 | 4 | 4 | 2 | 19 | | 0.04 | | Firmicutes;Clostridia;Clostridiales;Lachnospiraceae |
| 8797 | 2 | | 4 | 6 | 2 | 3 | 0 | 1 | 1 | 19 | | 0.04 | | Firmicutes;Clostridia;Clostridiales;Ruminococcaceae |
| 318 | 2 | | 1 | 7 | 2 | 1 | 1 | 1 | 4 | 19 | | 0.04 | | Chloroflexi;Anaerolineae |
| 14904 | 1 | | 1 | 0 | 3 | 4 | 2 | 5 | 3 | 19 | | 0.04 | | Firmicutes;Clostridia;Clostridiales;Ruminococcaceae |
| 17301 | 3 | | 2 | 2 | 3 | 1 | 5 | 2 | 1 | 19 | | 0.04 | | Firmicutes;Clostridia;Clostridiales;Lachnospiraceae;Lachnospiraceae Incertae Sedis |
| 9613 | 2 | | 4 | 0 | 4 | 2 | 4 | 3 | 0 | 19 | | 0.04 | | Firmicutes;Clostridia;Clostridiales;Incertae Sedis XIII;Anaerovorax |
| 19468 | 1 | | 3 | 1 | 4 | 1 | 2 | 3 | 4 | 19 | | 0.04 | | Bacteria |
| 10979 | 1 | | 5 | 1 | 4 | 0 | 2 | 0 | 6 | 19 | | 0.04 | | Firmicutes;Clostridia;Clostridiales |
| 18850 | 0 | | 1 | 4 | 5 | 1 | 4 | 3 | 1 | 19 | | 0.04 | | Firmicutes;Clostridia;Clostridiales |
| 184 | 2 | | 3 | 1 | 8 | 0 | 2 | 0 | 3 | 19 | | 0.04 | | Firmicutes;Clostridia;Clostridiales |
| 19900 | 0 | | 0 | 0 | 0 | 6 | 6 | 2 | 4 | 18 | | 0.04 | | Bacteroidetes;Bacteroidetes;Bacteroidales;Prevotellaceae |
| 16581 | 0 | | 0 | 0 | 0 | 2 | 6 | 4 | 6 | 18 | | 0.04 | | Firmicutes;Clostridia;Clostridiales;Lachnospiraceae |
| 7445 | 1 | | 0 | 0 | 0 | 5 | 5 | 4 | 3 | 18 | | 0.04 | | Bacteroidetes;Bacteroidetes;Bacteroidales;Prevotellaceae;Prevotella |
| 12681 | 0 | | 1 | 0 | 1 | 1 | 10 | 5 | 0 | 18 | | 0.04 | | Bacteroidetes;Bacteroidetes;Bacteroidales |
| 319 | 3 | | 3 | 2 | 1 | 2 | 2 | 2 | 3 | 18 | | 0.04 | | Firmicutes;Clostridia;Clostridiales;Lachnospiraceae;Pseudobutyrivibrio |
| 2587 | 0 | | 0 | 0 | 2 | 3 | 5 | 4 | 4 | 18 | | 0.04 | | Bacteroidetes;Bacteroidetes;Bacteroidales |
| 19692 | 1 | | 7 | 0 | 2 | 1 | 1 | 3 | 3 | 18 | | 0.04 | | Firmicutes;Clostridia;Clostridiales;Lachnospiraceae |
| 12294 | 3 | | 1 | 1 | 2 | 1 | 3 | 3 | 4 | 18 | | 0.04 | | Chloroflexi;Anaerolineae |
| 10820 | 0 | | 0 | 2 | 2 | 3 | 5 | 2 | 4 | 18 | | 0.04 | | Bacteroidetes |
| 17948 | 1 | | 2 | 2 | 2 | 4 | 3 | 2 | 2 | 18 | | 0.04 | | Firmicutes;Clostridia;Clostridiales;Ruminococcaceae |
| 19119 | 3 | | 7 | 6 | 2 | 0 | 0 | 0 | 0 | 18 | | 0.04 | | Bacteroidetes;Bacteroidetes;Bacteroidales |
| 19716 | 1 | | 1 | 2 | 3 | 1 | 8 | 1 | 1 | 18 | | 0.04 | | Bacteroidetes;Bacteroidetes;Bacteroidales |
| 13048 | 1 | | 1 | 2 | 3 | 1 | 3 | 2 | 5 | 18 | | 0.04 | | Firmicutes;Clostridia;Clostridiales;Ruminococcaceae |
| 8466 | 2 | | 2 | 2 | 3 | 1 | 2 | 1 | 5 | 18 | | 0.04 | | Firmicutes;Clostridia;Clostridiales;Lachnospiraceae |
| 17593 | 3 | | 2 | 3 | 3 | 2 | 1 | 3 | 1 | 18 | | 0.04 | | Firmicutes;Clostridia;Clostridiales |
| 2106 | 2 | | 3 | 2 | 4 | 2 | 3 | 0 | 2 | 18 | | 0.04 | | Planctomycetes;Planctomycetacia;Planctomycetales;Planctomycetaceae |
| 9408 | 4 | | 2 | 3 | 4 | 0 | 2 | 1 | 2 | 18 | | 0.04 | | Bacteria |
| 18284 | 0 | | 5 | 3 | 4 | 3 | 0 | 1 | 2 | 18 | | 0.04 | | Firmicutes;Clostridia;Clostridiales;Veillonellaceae;Succiniclasticum |
| 19330 | 2 | | 5 | 3 | 4 | 2 | 2 | 0 | 0 | 18 | | 0.04 | | Chloroflexi;Anaerolineae |
| 19481 | 2 | | 0 | 4 | 4 | 0 | 2 | 2 | 4 | 18 | | 0.04 | | Bacteroidetes |
| 15790 | 6 | | 2 | 3 | 5 | 0 | 1 | 0 | 1 | 18 | | 0.04 | | Firmicutes;Clostridia |
| 12040 | 2 | | 3 | 3 | 5 | 2 | 2 | 1 | 0 | 18 | | 0.04 | | Firmicutes;Clostridia;Clostridiales;Lachnospiraceae |
| 16369 | 1 | | 8 | 3 | 5 | 1 | 0 | 0 | 0 | 18 | | 0.04 | | Bacteroidetes;Bacteroidetes;Bacteroidales;Prevotellaceae;Prevotella |
| 465 | 0 | | 1 | 4 | 6 | 3 | 2 | 1 | 1 | 18 | | 0.04 | | Firmicutes;Clostridia;Clostridiales;Incertae Sedis XIII |
| 18904 | 0 | | 0 | 0 | 0 | 3 | 6 | 5 | 3 | 17 | | 0.04 | | Bacteria |
| 15651 | 0 | | 0 | 0 | 0 | 4 | 3 | 7 | 3 | 17 | | 0.04 | | Bacteroidetes |
| 5881 | 0 | | 0 | 0 | 0 | 7 | 1 | 5 | 4 | 17 | | 0.04 | | Bacteroidetes;Bacteroidetes;Bacteroidales;Prevotellaceae |
| 18211 | 0 | | 0 | 0 | 0 | 2 | 6 | 5 | 4 | 17 | | 0.04 | | Bacteroidetes;Bacteroidetes;Bacteroidales;Prevotellaceae;Prevotella |
| 21944 | 1 | | 0 | 0 | 0 | 3 | 1 | 6 | 6 | 17 | | 0.04 | | Bacteroidetes;Bacteroidetes;Bacteroidales |
| 19 | 1 | | 1 | 0 | 0 | 1 | 4 | 5 | 5 | 17 | | 0.04 | | Firmicutes |
| 13996 | 0 | | 0 | 1 | 0 | 3 | 8 | 4 | 1 | 17 | | 0.04 | | Firmicutes;Clostridia;Clostridiales;Lachnospiraceae |
| 3193 | 0 | | 0 | 1 | 0 | 5 | 1 | 7 | 3 | 17 | | 0.04 | | Firmicutes;Clostridia;Clostridiales;Lachnospiraceae |
| 1307 | 0 | | 0 | 1 | 0 | 3 | 3 | 4 | 6 | 17 | | 0.04 | | Bacteroidetes;Bacteroidetes;Bacteroidales |
| 133 | 1 | | 1 | 1 | 0 | 3 | 9 | 0 | 2 | 17 | | 0.04 | | Bacteroidetes;Bacteroidetes;Bacteroidales |
| 6955 | 1 | | 1 | 1 | 0 | 4 | 1 | 7 | 2 | 17 | | 0.04 | | TM7;TM7_genera_incertae_sedis |
| 14005 | 3 | | 1 | 1 | 0 | 1 | 3 | 4 | 4 | 17 | | 0.04 | | Bacteria |
| 65 | 1 | | 4 | 2 | 1 | 2 | 1 | 5 | 1 | 17 | | 0.04 | | Bacteroidetes |
| 18054 | 0 | | 1 | 1 | 2 | 3 | 3 | 4 | 3 | 17 | | 0.04 | | Bacteroidetes;Bacteroidetes;Bacteroidales;Prevotellaceae;Prevotella |
| 22197 | 0 | | 0 | 3 | 2 | 1 | 5 | 2 | 4 | 17 | | 0.04 | | Bacteroidetes;Bacteroidetes;Bacteroidales;Prevotellaceae |
| 15270 | 2 | | 3 | 3 | 2 | 1 | 4 | 2 | 0 | 17 | | 0.04 | | Firmicutes;Clostridia;Clostridiales;Lachnospiraceae |
| 10998 | 4 | | 5 | 4 | 2 | 0 | 1 | 1 | 0 | 17 | | 0.04 | | Bacteroidetes |
| 13905 | 0 | | 0 | 1 | 3 | 2 | 5 | 5 | 1 | 17 | | 0.04 | | Firmicutes;Clostridia;Clostridiales |
| 18788 | 0 | | 3 | 1 | 3 | 3 | 3 | 1 | 3 | 17 | | 0.04 | | Firmicutes |
| 8957 | 2 | | 0 | 2 | 3 | 2 | 2 | 4 | 2 | 17 | | 0.04 | | Firmicutes;Clostridia;Clostridiales;Veillonellaceae |
| 21966 | 5 | | 4 | 2 | 3 | 0 | 2 | 0 | 1 | 17 | | 0.04 | | Firmicutes;Clostridia;Clostridiales;Lachnospiraceae |
| 17911 | 3 | | 6 | 5 | 3 | 0 | 0 | 0 | 0 | 17 | | 0.04 | | Bacteroidetes;Bacteroidetes;Bacteroidales |
| 22287 | 0 | | 3 | 2 | 4 | 1 | 2 | 1 | 4 | 17 | | 0.04 | | Bacteria |
| 7399 | 3 | | 4 | 5 | 4 | 1 | 0 | 0 | 0 | 17 | | 0.04 | | Firmicutes;Clostridia;Clostridiales |
| 18971 | 1 | | 7 | 4 | 5 | 0 | 0 | 0 | 0 | 17 | | 0.04 | | Bacteroidetes;Bacteroidetes;Bacteroidales |
| 1161 | 1 | | 2 | 4 | 6 | 1 | 1 | 2 | 0 | 17 | | 0.04 | | Firmicutes;Clostridia;Clostridiales;Lachnospiraceae |
| 19981 | 0 | | 0 | 0 | 0 | 1 | 3 | 4 | 8 | 16 | | 0.03 | | Bacteroidetes;Bacteroidetes;Bacteroidales |
| 4464 | 2 | | 0 | 0 | 0 | 4 | 3 | 2 | 5 | 16 | | 0.03 | | Bacteria |
| 5800 | 0 | | 1 | 0 | 0 | 2 | 5 | 4 | 4 | 16 | | 0.03 | | Firmicutes;Clostridia;Clostridiales |
| 11636 | 0 | | 1 | 0 | 1 | 5 | 2 | 3 | 4 | 16 | | 0.03 | | Bacteroidetes;Bacteroidetes;Bacteroidales;Prevotellaceae;Prevotella |
| 19047 | 1 | | 1 | 1 | 1 | 2 | 4 | 2 | 4 | 16 | | 0.03 | | Firmicutes;Clostridia;Clostridiales;Veillonellaceae |
| 18800 | 0 | | 3 | 1 | 1 | 2 | 3 | 2 | 4 | 16 | | 0.03 | | Firmicutes;Clostridia;Clostridiales;Lachnospiraceae |
| 19897 | 2 | | 4 | 2 | 1 | 0 | 2 | 1 | 4 | 16 | | 0.03 | | Firmicutes;Clostridia;Clostridiales;Lachnospiraceae |
| 8049 | 0 | | 0 | 0 | 2 | 0 | 2 | 5 | 7 | 16 | | 0.03 | | Firmicutes;Clostridia;Clostridiales;Lachnospiraceae |
| 14114 | 2 | | 2 | 3 | 2 | 3 | 1 | 2 | 1 | 16 | | 0.03 | | Bacteria |
| 7332 | 0 | | 4 | 3 | 2 | 2 | 2 | 2 | 1 | 16 | | 0.03 | | Firmicutes;Clostridia;Clostridiales;Veillonellaceae |
| 18722 | 2 | | 0 | 0 | 3 | 1 | 5 | 3 | 2 | 16 | | 0.03 | | Firmicutes;Clostridia;Clostridiales |
| 2810 | 0 | | 3 | 2 | 3 | 3 | 1 | 3 | 1 | 16 | | 0.03 | | Firmicutes;Clostridia;Clostridiales |
| 8194 | 1 | | 3 | 3 | 3 | 2 | 0 | 2 | 2 | 16 | | 0.03 | | Firmicutes;Clostridia;Clostridiales;Lachnospiraceae;Lachnospiraceae Incertae Sedis |
| 9958 | 4 | | 3 | 2 | 4 | 1 | 0 | 1 | 1 | 16 | | 0.03 | | Firmicutes;Clostridia;Clostridiales;Lachnospiraceae |
| 19799 | 1 | | 1 | 4 | 4 | 2 | 2 | 2 | 0 | 16 | | 0.03 | | Bacteroidetes;Bacteroidetes;Bacteroidales |
| 17320 | 2 | | 3 | 4 | 4 | 0 | 2 | 1 | 0 | 16 | | 0.03 | | Bacteria |
| 7174 | 2 | | 1 | 5 | 4 | 2 | 1 | 0 | 1 | 16 | | 0.03 | | Bacteria |
| 17673 | 1 | | 2 | 1 | 5 | 2 | 2 | 1 | 2 | 16 | | 0.03 | | Firmicutes |
| 14124 | 0 | | 0 | 0 | 0 | 1 | 6 | 2 | 6 | 15 | | 0.03 | | Firmicutes;Clostridia;Clostridiales;Ruminococcaceae |
| 3249 | 0 | | 1 | 0 | 0 | 2 | 2 | 9 | 1 | 15 | | 0.03 | | Bacteroidetes |
| 3896 | 1 | | 1 | 0 | 0 | 2 | 5 | 1 | 5 | 15 | | 0.03 | | Bacteroidetes;Bacteroidetes;Bacteroidales;Prevotellaceae;Prevotella |
| 11085 | 0 | | 0 | 1 | 0 | 2 | 5 | 4 | 3 | 15 | | 0.03 | | Firmicutes;Clostridia;Clostridiales;Lachnospiraceae;Lachnospiraceae Incertae Sedis |
| 16314 | 1 | | 0 | 1 | 0 | 4 | 2 | 6 | 1 | 15 | | 0.03 | | Firmicutes;Clostridia;Clostridiales;Lachnospiraceae |
| 2544 | 1 | | 1 | 1 | 0 | 0 | 3 | 5 | 4 | 15 | | 0.03 | | Bacteroidetes;Bacteroidetes;Bacteroidales;Prevotellaceae |
| 9375 | 3 | | 2 | 1 | 0 | 1 | 4 | 1 | 3 | 15 | | 0.03 | | Firmicutes;Clostridia;Clostridiales;Ruminococcaceae |
| 16002 | 2 | | 1 | 0 | 1 | 3 | 1 | 4 | 3 | 15 | | 0.03 | | Bacteroidetes;Bacteroidetes;Bacteroidales |
| 22231 | 0 | | 0 | 1 | 1 | 7 | 1 | 3 | 2 | 15 | | 0.03 | | Bacteroidetes;Bacteroidetes;Bacteroidales;Prevotellaceae;Hallella |
| 10003 | 0 | | 1 | 1 | 1 | 0 | 4 | 4 | 4 | 15 | | 0.03 | | Bacteroidetes |
| 1630 | 3 | | 4 | 1 | 1 | 1 | 3 | 1 | 1 | 15 | | 0.03 | | Firmicutes;Clostridia;Clostridiales;Ruminococcaceae |
| 1002 | 0 | | 2 | 0 | 2 | 2 | 4 | 1 | 4 | 15 | | 0.03 | | Firmicutes;Clostridia;Clostridiales |
| 19757 | 1 | | 2 | 0 | 2 | 1 | 4 | 3 | 2 | 15 | | 0.03 | | Bacteroidetes;Bacteroidetes;Bacteroidales |
| 4211 | 4 | | 5 | 1 | 2 | 1 | 1 | 1 | 0 | 15 | | 0.03 | | Firmicutes;Clostridia;Clostridiales;Lachnospiraceae |
| 15214 | 0 | | 0 | 2 | 2 | 0 | 3 | 2 | 6 | 15 | | 0.03 | | Bacteroidetes;Bacteroidetes;Bacteroidales |
| 10637 | 0 | | 3 | 2 | 2 | 3 | 1 | 2 | 2 | 15 | | 0.03 | | Firmicutes;Clostridia;Clostridiales |
| 5867 | 1 | | 4 | 2 | 2 | 0 | 2 | 2 | 2 | 15 | | 0.03 | | Firmicutes;Clostridia;Clostridiales |
| 15610 | 5 | | 3 | 3 | 2 | 0 | 2 | 0 | 0 | 15 | | 0.03 | | Firmicutes;Clostridia;Clostridiales;Lachnospiraceae;Syntrophococcus |
| 5493 | 0 | | 2 | 5 | 2 | 2 | 2 | 0 | 2 | 15 | | 0.03 | | Firmicutes |
| 23698 | 2 | | 2 | 0 | 3 | 2 | 1 | 0 | 5 | 15 | | 0.03 | | Firmicutes;Clostridia;Clostridiales;Ruminococcaceae |
| 7644 | 1 | | 0 | 1 | 3 | 0 | 6 | 3 | 1 | 15 | | 0.03 | | Bacteroidetes;Bacteroidetes;Bacteroidales |
| 15695 | 2 | | 4 | 2 | 3 | 2 | 0 | 2 | 0 | 15 | | 0.03 | | Firmicutes;Clostridia;Clostridiales;Ruminococcaceae |
| 14288 | 0 | | 3 | 3 | 3 | 1 | 1 | 4 | 0 | 15 | | 0.03 | | Firmicutes;Clostridia |
| 22468 | 2 | | 4 | 4 | 3 | 0 | 1 | 1 | 0 | 15 | | 0.03 | | Firmicutes;Clostridia;Clostridiales |
| 20822 | 4 | | 2 | 0 | 4 | 1 | 2 | 1 | 1 | 15 | | 0.03 | | Firmicutes;Clostridia |
| 7756 | 2 | | 0 | 1 | 4 | 0 | 5 | 3 | 0 | 15 | | 0.03 | | Firmicutes;Clostridia;Clostridiales |
| 23188 | 1 | | 6 | 1 | 4 | 0 | 1 | 2 | 0 | 15 | | 0.03 | | Firmicutes;Clostridia |
| 16272 | 1 | | 3 | 2 | 4 | 1 | 0 | 2 | 2 | 15 | | 0.03 | | Firmicutes;Clostridia;Clostridiales;Ruminococcaceae |
| 1267 | 3 | | 5 | 3 | 4 | 0 | 0 | 0 | 0 | 15 | | 0.03 | | Bacteria |
| 6959 | 2 | | 3 | 1 | 6 | 0 | 0 | 1 | 2 | 15 | | 0.03 | | Firmicutes;Clostridia;Clostridiales |
| 21002 | 1 | | 6 | 2 | 6 | 0 | 0 | 0 | 0 | 15 | | 0.03 | | Firmicutes;Clostridia;Clostridiales;Lachnospiraceae |
| 20170 | 0 | | 0 | 0 | 0 | 4 | 5 | 3 | 2 | 14 | | 0.03 | | Bacteria |
| 8830 | 0 | | 0 | 0 | 0 | 5 | 2 | 4 | 3 | 14 | | 0.03 | | Bacteroidetes;Bacteroidetes;Bacteroidales;Prevotellaceae;Prevotella |
| 17698 | 0 | | 0 | 0 | 0 | 1 | 6 | 3 | 4 | 14 | | 0.03 | | Firmicutes;Clostridia;Clostridiales;Ruminococcaceae |
| 13952 | 3 | | 0 | 0 | 0 | 3 | 3 | 4 | 1 | 14 | | 0.03 | | Bacteria |
| 412 | 0 | | 1 | 0 | 0 | 2 | 5 | 1 | 5 | 14 | | 0.03 | | Bacteroidetes;Bacteroidetes;Bacteroidales;Prevotellaceae;Prevotella |
| 12007 | 2 | | 2 | 0 | 0 | 0 | 3 | 3 | 4 | 14 | | 0.03 | | Bacteria |
| 3058 | 0 | | 1 | 1 | 0 | 3 | 5 | 2 | 2 | 14 | | 0.03 | | Firmicutes;Clostridia;Clostridiales;Ruminococcaceae;Anaerotruncus |
| 21695 | 1 | | 1 | 2 | 0 | 2 | 2 | 1 | 5 | 14 | | 0.03 | | Firmicutes;Clostridia;Clostridiales |
| 18141 | 2 | | 0 | 0 | 1 | 1 | 5 | 3 | 2 | 14 | | 0.03 | | Bacteria |
| 11022 | 0 | | 1 | 0 | 1 | 3 | 5 | 0 | 4 | 14 | | 0.03 | | Bacteroidetes;Bacteroidetes;Bacteroidales |
| 767 | 1 | | 1 | 0 | 1 | 1 | 2 | 4 | 4 | 14 | | 0.03 | | Bacteria |
| 22894 | 0 | | 1 | 1 | 1 | 4 | 2 | 3 | 2 | 14 | | 0.03 | | Firmicutes;Clostridia |
| 19025 | 2 | | 1 | 1 | 1 | 2 | 1 | 2 | 4 | 14 | | 0.03 | | Bacteroidetes;Bacteroidetes;Bacteroidales |
| 19645 | 0 | | 7 | 1 | 1 | 1 | 2 | 2 | 0 | 14 | | 0.03 | | Firmicutes;Clostridia |
| 20903 | 3 | | 7 | 3 | 1 | 0 | 0 | 0 | 0 | 14 | | 0.03 | | Bacteroidetes |
| 10924 | 1 | | 0 | 0 | 2 | 3 | 1 | 2 | 5 | 14 | | 0.03 | | Firmicutes;Clostridia;Clostridiales;Ruminococcaceae |
| 20339 | 1 | | 1 | 0 | 2 | 2 | 4 | 2 | 2 | 14 | | 0.03 | | Bacteria |
| 16572 | 2 | | 1 | 1 | 2 | 4 | 1 | 2 | 1 | 14 | | 0.03 | | Bacteroidetes;Bacteroidetes;Bacteroidales |
| 21868 | 2 | | 1 | 1 | 2 | 0 | 2 | 4 | 2 | 14 | | 0.03 | | Bacteroidetes |
| 12368 | 2 | | 2 | 1 | 2 | 1 | 2 | 1 | 3 | 14 | | 0.03 | | Bacteroidetes;Bacteroidetes;Bacteroidales;Prevotellaceae |
| 4657 | 1 | | 0 | 2 | 2 | 1 | 4 | 3 | 1 | 14 | | 0.03 | | Firmicutes;Clostridia;Clostridiales;Ruminococcaceae |
| 9907 | 0 | | 1 | 3 | 2 | 1 | 1 | 3 | 3 | 14 | | 0.03 | | Firmicutes;Clostridia;Clostridiales;Ruminococcaceae;Ruminococcus |
| 18796 | 0 | | 2 | 0 | 3 | 2 | 2 | 1 | 4 | 14 | | 0.03 | | Firmicutes;Clostridia;Clostridiales;Incertae Sedis XIII;Anaerovorax |
| 5437 | 1 | | 0 | 1 | 3 | 0 | 3 | 2 | 4 | 14 | | 0.03 | | Firmicutes;Clostridia;Clostridiales |
| 9530 | 0 | | 1 | 1 | 3 | 2 | 3 | 1 | 3 | 14 | | 0.03 | | Bacteria |
| 18643 | 2 | | 1 | 3 | 3 | 1 | 3 | 1 | 0 | 14 | | 0.03 | | Firmicutes;Clostridia;Clostridiales;Ruminococcaceae |
| 4174 | 0 | | 3 | 3 | 3 | 2 | 1 | 1 | 1 | 14 | | 0.03 | | Firmicutes;Clostridia;Clostridiales |
| 8507 | 0 | | 2 | 4 | 3 | 1 | 2 | 1 | 1 | 14 | | 0.03 | | Bacteroidetes;Bacteroidetes;Bacteroidales;Prevotellaceae;Prevotella |
| 15343 | 0 | | 1 | 1 | 4 | 2 | 4 | 0 | 2 | 14 | | 0.03 | | Bacteria |
| 15364 | 2 | | 1 | 1 | 4 | 0 | 1 | 3 | 2 | 14 | | 0.03 | | Firmicutes;Clostridia;Clostridiales |
| 15199 | 3 | | 1 | 1 | 4 | 4 | 0 | 0 | 1 | 14 | | 0.03 | | Firmicutes;Clostridia;Clostridiales;Lachnospiraceae |
| 18799 | 3 | | 5 | 2 | 4 | 0 | 0 | 0 | 0 | 14 | | 0.03 | | Bacteroidetes;Bacteroidetes;Bacteroidales;Prevotellaceae |
| 6241 | 2 | | 1 | 3 | 4 | 0 | 2 | 1 | 1 | 14 | | 0.03 | | Firmicutes;Clostridia;Clostridiales;Ruminococcaceae |
| 5130 | 3 | | 1 | 4 | 4 | 1 | 0 | 1 | 0 | 14 | | 0.03 | | Firmicutes;Clostridia;Clostridiales;Ruminococcaceae |
| 2476 | 1 | | 2 | 6 | 5 | 0 | 0 | 0 | 0 | 14 | | 0.03 | | Bacteroidetes;Bacteroidetes;Bacteroidales;Prevotellaceae |
| 4993 | 1 | | 4 | 2 | 7 | 0 | 0 | 0 | 0 | 14 | | 0.03 | | Bacteroidetes;Bacteroidetes;Bacteroidales;Prevotellaceae;Hallella |
| 9158 | 0 | | 0 | 0 | 0 | 2 | 7 | 1 | 3 | 13 | | 0.03 | | Bacteroidetes;Bacteroidetes;Bacteroidales;Prevotellaceae;Prevotella |
| 23174 | 0 | | 0 | 0 | 0 | 2 | 3 | 5 | 3 | 13 | | 0.03 | | Firmicutes;Clostridia;Clostridiales;Ruminococcaceae |
| 113 | 0 | | 0 | 0 | 0 | 1 | 4 | 3 | 5 | 13 | | 0.03 | | Bacteria |
| 18918 | 0 | | 1 | 0 | 0 | 1 | 3 | 2 | 6 | 13 | | 0.03 | | Bacteria |
| 4676 | 0 | | 1 | 1 | 0 | 1 | 1 | 3 | 6 | 13 | | 0.03 | | Bacteria |
| 12182 | 0 | | 0 | 0 | 1 | 2 | 6 | 2 | 2 | 13 | | 0.03 | | Bacteroidetes;Bacteroidetes;Bacteroidales;Prevotellaceae;Prevotella |
| 11416 | 0 | | 0 | 0 | 1 | 4 | 2 | 3 | 3 | 13 | | 0.03 | | Firmicutes;Clostridia;Clostridiales |
| 1541 | 1 | | 0 | 0 | 1 | 1 | 4 | 3 | 3 | 13 | | 0.03 | | Firmicutes;Clostridia |
| 12687 | 0 | | 2 | 0 | 1 | 1 | 1 | 2 | 6 | 13 | | 0.03 | | Bacteroidetes |
| 2960 | 3 | | 3 | 0 | 1 | 0 | 3 | 1 | 2 | 13 | | 0.03 | | Bacteroidetes;Bacteroidetes;Bacteroidales;Prevotellaceae |
| 21085 | 0 | | 0 | 1 | 1 | 3 | 2 | 3 | 3 | 13 | | 0.03 | | Bacteroidetes |
| 10203 | 4 | | 4 | 1 | 1 | 2 | 0 | 0 | 1 | 13 | | 0.03 | | Firmicutes;Clostridia;Clostridiales;Lachnospiraceae |
| 22592 | 0 | | 0 | 2 | 1 | 3 | 3 | 1 | 3 | 13 | | 0.03 | | Firmicutes;Clostridia;Clostridiales;Lachnospiraceae;Lachnospiraceae Incertae Sedis |
| 20869 | 0 | | 0 | 2 | 1 | 1 | 3 | 3 | 3 | 13 | | 0.03 | | Firmicutes;Clostridia;Clostridiales |
| 14026 | 1 | | 1 | 2 | 1 | 1 | 1 | 2 | 4 | 13 | | 0.03 | | Bacteria |
| 1611 | 1 | | 5 | 6 | 1 | 0 | 0 | 0 | 0 | 13 | | 0.03 | | Bacteroidetes |
| 21350 | 1 | | 4 | 1 | 2 | 0 | 2 | 1 | 2 | 13 | | 0.03 | | Firmicutes;Clostridia;Clostridiales;Ruminococcaceae |
| 6055 | 2 | | 0 | 2 | 2 | 2 | 4 | 1 | 0 | 13 | | 0.03 | | Firmicutes;Clostridia;Clostridiales;Lachnospiraceae |
| 8565 | 0 | | 1 | 2 | 2 | 0 | 3 | 3 | 2 | 13 | | 0.03 | | Firmicutes;Clostridia;Clostridiales;Lachnospiraceae |
| 18487 | 2 | | 5 | 2 | 2 | 0 | 0 | 2 | 0 | 13 | | 0.03 | | Firmicutes;Clostridia;Clostridiales |
| 19905 | 1 | | 6 | 4 | 2 | 0 | 0 | 0 | 0 | 13 | | 0.03 | | Bacteria |
| 17451 | 1 | | 3 | 3 | 3 | 0 | 3 | 0 | 0 | 13 | | 0.03 | | Firmicutes;Clostridia |
| 20944 | 1 | | 1 | 1 | 4 | 0 | 1 | 3 | 2 | 13 | | 0.03 | | Bacteria |
| 9079 | 2 | | 5 | 2 | 4 | 0 | 0 | 0 | 0 | 13 | | 0.03 | | Bacteroidetes |
| 1064 | 0 | | 2 | 0 | 5 | 1 | 3 | 1 | 1 | 13 | | 0.03 | | Firmicutes;Clostridia;Clostridiales;Ruminococcaceae |
| 2282 | 2 | | 3 | 1 | 5 | 1 | 0 | 1 | 0 | 13 | | 0.03 | | Firmicutes;Clostridia;Clostridiales;Lachnospiraceae |
| 4766 | 0 | | 0 | 0 | 0 | 1 | 6 | 2 | 3 | 12 | | 0.03 | | Bacteroidetes;Bacteroidetes;Bacteroidales |
| 13647 | 0 | | 0 | 0 | 0 | 3 | 3 | 3 | 3 | 12 | | 0.03 | | Bacteria |
| 4578 | 0 | | 0 | 0 | 0 | 1 | 5 | 3 | 3 | 12 | | 0.03 | | Bacteroidetes;Bacteroidetes;Bacteroidales;Prevotellaceae;Prevotella |
| 22244 | 0 | | 0 | 0 | 0 | 3 | 1 | 5 | 3 | 12 | | 0.03 | | Bacteroidetes;Bacteroidetes;Bacteroidales |
| 16965 | 0 | | 0 | 0 | 0 | 2 | 4 | 2 | 4 | 12 | | 0.03 | | Bacteroidetes;Bacteroidetes;Bacteroidales |
| 357 | 1 | | 0 | 0 | 0 | 4 | 2 | 4 | 1 | 12 | | 0.03 | | Firmicutes;Clostridia;Clostridiales;Incertae Sedis XIII;Anaerovorax |
| 22763 | 1 | | 0 | 0 | 0 | 0 | 2 | 3 | 6 | 12 | | 0.03 | | Bacteria |
| 8183 | 0 | | 1 | 0 | 0 | 9 | 1 | 1 | 0 | 12 | | 0.03 | | Bacteroidetes |
| 11870 | 0 | | 0 | 2 | 0 | 1 | 1 | 4 | 4 | 12 | | 0.03 | | Bacteroidetes;Bacteroidetes;Bacteroidales |
| 9160 | 0 | | 0 | 2 | 0 | 2 | 1 | 2 | 5 | 12 | | 0.03 | | Bacteroidetes;Bacteroidetes;Bacteroidales |
| 22042 | 1 | | 2 | 2 | 0 | 1 | 2 | 2 | 2 | 12 | | 0.03 | | Firmicutes;Clostridia;Clostridiales |
| 19115 | 1 | | 2 | 2 | 0 | 2 | 0 | 2 | 3 | 12 | | 0.03 | | Firmicutes;Clostridia;Clostridiales |
| 5874 | 0 | | 0 | 0 | 1 | 0 | 2 | 4 | 5 | 12 | | 0.03 | | Bacteria |
| 14110 | 0 | | 1 | 0 | 1 | 2 | 3 | 1 | 4 | 12 | | 0.03 | | Bacteria |
| 10477 | 1 | | 1 | 0 | 1 | 0 | 5 | 2 | 2 | 12 | | 0.03 | | Chloroflexi;Anaerolineae |
| 3613 | 0 | | 4 | 0 | 1 | 0 | 4 | 1 | 2 | 12 | | 0.03 | | Firmicutes;Clostridia;Clostridiales;Ruminococcaceae |
| 17716 | 0 | | 2 | 1 | 1 | 1 | 4 | 2 | 1 | 12 | | 0.03 | | Bacteria |
| 20055 | 1 | | 1 | 2 | 1 | 1 | 3 | 2 | 1 | 12 | | 0.03 | | Bacteroidetes;Bacteroidetes;Bacteroidales |
| 13021 | 0 | | 4 | 2 | 1 | 0 | 5 | 0 | 0 | 12 | | 0.03 | | Firmicutes;Clostridia;Clostridiales;Ruminococcaceae |
| 13983 | 1 | | 4 | 2 | 1 | 1 | 1 | 1 | 1 | 12 | | 0.03 | | Firmicutes;Clostridia;Clostridiales;Lachnospiraceae;Syntrophococcus |
| 399 | 4 | | 3 | 3 | 1 | 0 | 0 | 1 | 0 | 12 | | 0.03 | | Firmicutes;Clostridia;Clostridiales;Lachnospiraceae |
| 14998 | 1 | | 0 | 4 | 1 | 1 | 3 | 0 | 2 | 12 | | 0.03 | | Firmicutes;Clostridia;Clostridiales;Ruminococcaceae |
| 10711 | 0 | | 0 | 0 | 2 | 4 | 3 | 1 | 2 | 12 | | 0.03 | | Bacteroidetes;Bacteroidetes;Bacteroidales |
| 10287 | 1 | | 0 | 0 | 2 | 4 | 1 | 2 | 2 | 12 | | 0.03 | | Bacteroidetes |
| 1545 | 1 | | 0 | 0 | 2 | 0 | 1 | 2 | 6 | 12 | | 0.03 | | Bacteroidetes;Bacteroidetes;Bacteroidales;Prevotellaceae |
| 10194 | 2 | | 1 | 0 | 2 | 0 | 4 | 2 | 1 | 12 | | 0.03 | | Firmicutes;Clostridia;Clostridiales;Lachnospiraceae |
| 3997 | 0 | | 0 | 1 | 2 | 0 | 5 | 3 | 1 | 12 | | 0.03 | | Bacteroidetes;Bacteroidetes;Bacteroidales |
| 4693 | 0 | | 2 | 1 | 2 | 0 | 3 | 0 | 4 | 12 | | 0.03 | | Firmicutes;Clostridia;Clostridiales;Ruminococcaceae |
| 6723 | 0 | | 0 | 2 | 2 | 2 | 3 | 1 | 2 | 12 | | 0.03 | | Bacteria |
| 10164 | 0 | | 1 | 2 | 2 | 1 | 1 | 1 | 4 | 12 | | 0.03 | | Bacteroidetes;Bacteroidetes;Bacteroidales;Prevotellaceae |
| 2245 | 1 | | 4 | 3 | 2 | 1 | 0 | 0 | 1 | 12 | | 0.03 | | Bacteroidetes |
| 1763 | 1 | | 6 | 3 | 2 | 0 | 0 | 0 | 0 | 12 | | 0.03 | | Firmicutes;Clostridia;Clostridiales;Ruminococcaceae;Ruminococcus |
| 8125 | 2 | | 0 | 0 | 3 | 3 | 1 | 0 | 3 | 12 | | 0.03 | | Bacteria |
| 14066 | 0 | | 2 | 0 | 3 | 1 | 3 | 0 | 3 | 12 | | 0.03 | | Firmicutes;Clostridia;Clostridiales |
| 17933 | 0 | | 0 | 2 | 3 | 1 | 1 | 1 | 4 | 12 | | 0.03 | | Firmicutes;Clostridia;Clostridiales;Ruminococcaceae |
| 3437 | 1 | | 1 | 3 | 3 | 0 | 1 | 2 | 1 | 12 | | 0.03 | | Bacteroidetes;Bacteroidetes;Bacteroidales |
| 13188 | 1 | | 4 | 4 | 3 | 0 | 0 | 0 | 0 | 12 | | 0.03 | | Bacteroidetes;Bacteroidetes;Bacteroidales;Prevotellaceae |
| 23153 | 1 | | 4 | 4 | 3 | 0 | 0 | 0 | 0 | 12 | | 0.03 | | Bacteroidetes;Bacteroidetes;Bacteroidales;Prevotellaceae |
| 8279 | 0 | | 0 | 0 | 4 | 1 | 4 | 2 | 1 | 12 | | 0.03 | | Firmicutes;Clostridia;Clostridiales |
| 2660 | 0 | | 0 | 1 | 4 | 1 | 4 | 0 | 2 | 12 | | 0.03 | | Firmicutes;Clostridia;Clostridiales;Lachnospiraceae |
| 627 | 2 | | 2 | 2 | 4 | 0 | 1 | 1 | 0 | 12 | | 0.03 | | Firmicutes;Clostridia;Clostridiales;Incertae Sedis XIII;Anaerovorax |
| 13969 | 0 | | 1 | 3 | 4 | 0 | 1 | 1 | 2 | 12 | | 0.03 | | Firmicutes;Clostridia;Clostridiales |
| 3492 | 3 | | 2 | 3 | 4 | 0 | 0 | 0 | 0 | 12 | | 0.03 | | Bacteroidetes |
| 12384 | 0 | | 2 | 4 | 4 | 1 | 1 | 0 | 0 | 12 | | 0.03 | | Firmicutes;Clostridia;Clostridiales;Lachnospiraceae |
| 4809 | 1 | | 3 | 4 | 4 | 0 | 0 | 0 | 0 | 12 | | 0.03 | | Bacteroidetes |
| 19698 | 0 | | 2 | 2 | 5 | 1 | 0 | 2 | 0 | 12 | | 0.03 | | Firmicutes;Clostridia;Clostridiales;Ruminococcaceae |
| 18401 | 1 | | 2 | 2 | 5 | 0 | 1 | 0 | 1 | 12 | | 0.03 | | Bacteroidetes;Bacteroidetes;Bacteroidales;Prevotellaceae |
| 9878 | 3 | | 2 | 2 | 5 | 0 | 0 | 0 | 0 | 12 | | 0.03 | | Bacteria |
| 4954 | 2 | | 2 | 2 | 6 | 0 | 0 | 0 | 0 | 12 | | 0.03 | | Bacteroidetes;Bacteroidetes;Bacteroidales |
| 23659 | 0 | | 0 | 0 | 0 | 3 | 3 | 1 | 4 | 11 | | 0.02 | | Firmicutes;Clostridia;Clostridiales;Lachnospiraceae |
| 2985 | 0 | | 0 | 0 | 0 | 3 | 2 | 2 | 4 | 11 | | 0.02 | | Bacteroidetes;Bacteroidetes;Bacteroidales;Prevotellaceae;Prevotella |
| 11222 | 0 | | 1 | 0 | 0 | 1 | 5 | 3 | 1 | 11 | | 0.02 | | Bacteria |
| 23604 | 1 | | 1 | 0 | 0 | 1 | 5 | 1 | 2 | 11 | | 0.02 | | Bacteroidetes |
| 13896 | 1 | | 1 | 0 | 0 | 1 | 2 | 4 | 2 | 11 | | 0.02 | | Firmicutes;Clostridia;Clostridiales;Ruminococcaceae;Ruminococcus |
| 3295 | 0 | | 2 | 0 | 0 | 1 | 4 | 2 | 2 | 11 | | 0.02 | | Bacteria |
| 19685 | 0 | | 2 | 0 | 0 | 1 | 4 | 1 | 3 | 11 | | 0.02 | | Firmicutes;Clostridia;Clostridiales;Lachnospiraceae |
| 7893 | 1 | | 2 | 0 | 0 | 4 | 1 | 2 | 1 | 11 | | 0.02 | | Firmicutes;Clostridia;Clostridiales |
| 19821 | 8 | | 0 | 1 | 0 | 1 | 1 | 0 | 0 | 11 | | 0.02 | | Bacteroidetes;Bacteroidetes;Bacteroidales |
| 23257 | 1 | | 3 | 1 | 0 | 2 | 3 | 1 | 0 | 11 | | 0.02 | | Spirochaetes;Spirochaetes;Spirochaetales;Spirochaetaceae;Treponema |
| 14190 | 0 | | 0 | 2 | 0 | 3 | 2 | 2 | 2 | 11 | | 0.02 | | Bacteroidetes;Bacteroidetes;Bacteroidales |
| 4628 | 0 | | 0 | 0 | 1 | 2 | 4 | 2 | 2 | 11 | | 0.02 | | Firmicutes |
| 5380 | 0 | | 0 | 0 | 1 | 1 | 0 | 4 | 5 | 11 | | 0.02 | | Firmicutes;Clostridia;Clostridiales;Ruminococcaceae |
| 18111 | 0 | | 1 | 0 | 1 | 2 | 3 | 1 | 3 | 11 | | 0.02 | | Firmicutes;Clostridia;Clostridiales |
| 5674 | 0 | | 2 | 1 | 1 | 0 | 3 | 2 | 2 | 11 | | 0.02 | | Spirochaetes;Spirochaetes;Spirochaetales;Spirochaetaceae;Treponema |
| 19104 | 0 | | 2 | 1 | 1 | 1 | 3 | 0 | 3 | 11 | | 0.02 | | Bacteria |
| 58 | 1 | | 2 | 1 | 1 | 0 | 3 | 1 | 2 | 11 | | 0.02 | | Bacteroidetes;Bacteroidetes;Bacteroidales |
| 7961 | 0 | | 0 | 2 | 1 | 3 | 2 | 1 | 2 | 11 | | 0.02 | | Bacteroidetes;Bacteroidetes;Bacteroidales |
| 1747 | 0 | | 1 | 2 | 1 | 5 | 0 | 1 | 1 | 11 | | 0.02 | | Bacteroidetes |
| 20858 | 0 | | 2 | 2 | 1 | 2 | 1 | 0 | 3 | 11 | | 0.02 | | Bacteria |
| 21481 | 0 | | 3 | 2 | 1 | 1 | 1 | 3 | 0 | 11 | | 0.02 | | Firmicutes;Clostridia;Clostridiales;Ruminococcaceae |
| 16806 | 0 | | 2 | 3 | 1 | 1 | 1 | 1 | 2 | 11 | | 0.02 | | Firmicutes;Clostridia;Clostridiales;Lachnospiraceae |
| 19015 | 0 | | 0 | 0 | 2 | 3 | 0 | 2 | 4 | 11 | | 0.02 | | Firmicutes |
| 12329 | 1 | | 1 | 0 | 2 | 1 | 3 | 2 | 1 | 11 | | 0.02 | | Bacteroidetes;Bacteroidetes;Bacteroidales |
| 979 | 0 | | 2 | 0 | 2 | 3 | 2 | 1 | 1 | 11 | | 0.02 | | Firmicutes;Clostridia;Clostridiales;Ruminococcaceae |
| 19140 | 1 | | 2 | 0 | 2 | 1 | 3 | 1 | 1 | 11 | | 0.02 | | Firmicutes;Clostridia;Clostridiales |
| 15174 | 1 | | 3 | 0 | 2 | 1 | 2 | 1 | 1 | 11 | | 0.02 | | Firmicutes;Clostridia;Clostridiales |
| 20153 | 0 | | 0 | 1 | 2 | 1 | 4 | 1 | 2 | 11 | | 0.02 | | Bacteroidetes;Bacteroidetes;Bacteroidales |
| 15406 | 0 | | 0 | 1 | 2 | 2 | 2 | 2 | 2 | 11 | | 0.02 | | Firmicutes;Clostridia;Clostridiales;Lachnospiraceae |
| 9027 | 0 | | 0 | 1 | 2 | 1 | 1 | 1 | 5 | 11 | | 0.02 | | Firmicutes;Clostridia;Clostridiales;Veillonellaceae |
| 3263 | 2 | | 6 | 1 | 2 | 0 | 0 | 0 | 0 | 11 | | 0.02 | | Bacteroidetes;Bacteroidetes;Bacteroidales |
| 11143 | 0 | | 1 | 4 | 2 | 3 | 1 | 0 | 0 | 11 | | 0.02 | | Firmicutes;Clostridia |
| 11719 | 2 | | 3 | 4 | 2 | 0 | 0 | 0 | 0 | 11 | | 0.02 | | Bacteroidetes;Bacteroidetes;Bacteroidales;Prevotellaceae |
| 4884 | 1 | | 0 | 0 | 3 | 2 | 1 | 2 | 2 | 11 | | 0.02 | | Firmicutes;Clostridia;Clostridiales;Lachnospiraceae |
| 2665 | 1 | | 1 | 0 | 3 | 0 | 1 | 3 | 2 | 11 | | 0.02 | | Firmicutes;Clostridia;Clostridiales;Ruminococcaceae |
| 16990 | 0 | | 1 | 1 | 3 | 1 | 0 | 3 | 2 | 11 | | 0.02 | | Firmicutes;Clostridia;Clostridiales;Ruminococcaceae |
| 23403 | 1 | | 1 | 1 | 3 | 1 | 1 | 1 | 2 | 11 | | 0.02 | | Bacteroidetes;Bacteroidetes;Bacteroidales |
| 20581 | 4 | | 3 | 1 | 3 | 0 | 0 | 0 | 0 | 11 | | 0.02 | | Bacteroidetes;Bacteroidetes;Bacteroidales |
| 3742 | 2 | | 4 | 1 | 3 | 0 | 1 | 0 | 0 | 11 | | 0.02 | | Bacteroidetes |
| 4516 | 1 | | 2 | 5 | 3 | 0 | 0 | 0 | 0 | 11 | | 0.02 | | Bacteroidetes;Bacteroidetes;Bacteroidales |
| 23650 | 1 | | 1 | 6 | 3 | 0 | 0 | 0 | 0 | 11 | | 0.02 | | Bacteroidetes |
| 14491 | 1 | | 4 | 2 | 4 | 0 | 0 | 0 | 0 | 11 | | 0.02 | | Bacteroidetes |
| 3964 | 1 | | 1 | 2 | 7 | 0 | 0 | 0 | 0 | 11 | | 0.02 | | Bacteria |
| 20305 | 0 | | 0 | 0 | 0 | 4 | 4 | 1 | 1 | 10 | | 0.02 | | Firmicutes;Clostridia;Clostridiales |
| 18651 | 0 | | 0 | 0 | 0 | 2 | 4 | 3 | 1 | 10 | | 0.02 | | Firmicutes;Clostridia;Clostridiales;Ruminococcaceae |
| 17035 | 0 | | 0 | 0 | 0 | 1 | 4 | 4 | 1 | 10 | | 0.02 | | Bacteroidetes;Bacteroidetes;Bacteroidales;Prevotellaceae;Prevotella |
| 13058 | 0 | | 0 | 0 | 0 | 3 | 2 | 3 | 2 | 10 | | 0.02 | | Bacteroidetes;Bacteroidetes;Bacteroidales;Prevotellaceae |
| 15430 | 0 | | 0 | 0 | 0 | 4 | 2 | 1 | 3 | 10 | | 0.02 | | Bacteroidetes;Bacteroidetes;Bacteroidales;Prevotellaceae |
| 23229 | 0 | | 0 | 0 | 0 | 2 | 3 | 2 | 3 | 10 | | 0.02 | | Bacteroidetes;Bacteroidetes;Bacteroidales;Prevotellaceae;Prevotella |
| 11715 | 0 | | 0 | 0 | 0 | 1 | 1 | 5 | 3 | 10 | | 0.02 | | Firmicutes;Clostridia;Clostridiales;Ruminococcaceae |
| 506 | 0 | | 0 | 0 | 0 | 2 | 1 | 3 | 4 | 10 | | 0.02 | | Bacteroidetes;Bacteroidetes;Bacteroidales;Prevotellaceae |
| 20004 | 0 | | 0 | 0 | 0 | 2 | 2 | 1 | 5 | 10 | | 0.02 | | Bacteroidetes;Bacteroidetes;Bacteroidales;Prevotellaceae |
| 7799 | 1 | | 0 | 0 | 0 | 4 | 1 | 0 | 4 | 10 | | 0.02 | | TM7;TM7_genera_incertae_sedis |
| 13903 | 1 | | 0 | 0 | 0 | 0 | 3 | 1 | 5 | 10 | | 0.02 | | Bacteroidetes;Bacteroidetes;Bacteroidales;Prevotellaceae;Prevotella |
| 7548 | 0 | | 1 | 0 | 0 | 1 | 3 | 3 | 2 | 10 | | 0.02 | | Bacteroidetes;Bacteroidetes;Bacteroidales;Prevotellaceae;Prevotella |
| 1753 | 0 | | 1 | 0 | 0 | 2 | 3 | 1 | 3 | 10 | | 0.02 | | Bacteroidetes;Bacteroidetes;Bacteroidales |
| 12077 | 0 | | 2 | 0 | 0 | 3 | 3 | 2 | 0 | 10 | | 0.02 | | Bacteroidetes;Bacteroidetes;Bacteroidales |
| 2147 | 0 | | 2 | 0 | 0 | 2 | 1 | 5 | 0 | 10 | | 0.02 | | Bacteroidetes;Bacteroidetes;Bacteroidales |
| 13344 | 0 | | 0 | 1 | 0 | 4 | 0 | 2 | 3 | 10 | | 0.02 | | Firmicutes;Clostridia |
| 5963 | 2 | | 0 | 1 | 0 | 1 | 1 | 3 | 2 | 10 | | 0.02 | | Firmicutes;Clostridia |
| 19052 | 0 | | 0 | 2 | 0 | 1 | 3 | 1 | 3 | 10 | | 0.02 | | Bacteroidetes |
| 6111 | 1 | | 0 | 2 | 0 | 1 | 3 | 2 | 1 | 10 | | 0.02 | | Bacteroidetes |
| 11851 | 0 | | 5 | 2 | 0 | 0 | 0 | 1 | 2 | 10 | | 0.02 | | Firmicutes;Clostridia;Clostridiales;Ruminococcaceae |
| 7035 | 0 | | 0 | 0 | 1 | 2 | 3 | 1 | 3 | 10 | | 0.02 | | Firmicutes;Clostridia;Clostridiales |
| 21360 | 1 | | 0 | 0 | 1 | 4 | 2 | 2 | 0 | 10 | | 0.02 | | Firmicutes;Clostridia |
| 2137 | 1 | | 0 | 0 | 1 | 0 | 5 | 3 | 0 | 10 | | 0.02 | | Bacteroidetes |
| 9995 | 2 | | 0 | 0 | 1 | 1 | 1 | 5 | 0 | 10 | | 0.02 | | Firmicutes;Clostridia;Clostridiales;Lachnospiraceae |
| 12395 | 2 | | 2 | 0 | 1 | 1 | 3 | 1 | 0 | 10 | | 0.02 | | TM7;TM7_genera_incertae_sedis |
| 5916 | 0 | | 0 | 1 | 1 | 2 | 2 | 3 | 1 | 10 | | 0.02 | | Bacteria |
| 21000 | 0 | | 0 | 1 | 1 | 2 | 1 | 3 | 2 | 10 | | 0.02 | | Firmicutes;Clostridia;Clostridiales;Lachnospiraceae |
| 16586 | 0 | | 0 | 1 | 1 | 1 | 1 | 2 | 4 | 10 | | 0.02 | | Bacteria |
| 10012 | 2 | | 0 | 1 | 1 | 2 | 2 | 1 | 1 | 10 | | 0.02 | | Bacteria |
| 9994 | 0 | | 1 | 1 | 1 | 2 | 0 | 2 | 3 | 10 | | 0.02 | | Bacteroidetes;Bacteroidetes;Bacteroidales |
| 5681 | 0 | | 1 | 1 | 1 | 1 | 1 | 1 | 4 | 10 | | 0.02 | | Firmicutes;Clostridia;Clostridiales |
| 4135 | 1 | | 1 | 1 | 1 | 0 | 4 | 1 | 1 | 10 | | 0.02 | | Firmicutes;Clostridia;Clostridiales;Incertae Sedis XIII;Anaerovorax |
| 20733 | 2 | | 2 | 5 | 1 | 0 | 0 | 0 | 0 | 10 | | 0.02 | | Bacteroidetes |
| 2981 | 0 | | 2 | 6 | 1 | 0 | 0 | 0 | 1 | 10 | | 0.02 | | Firmicutes;Clostridia;Clostridiales;Ruminococcaceae;Ruminococcus |
| 23210 | 0 | | 0 | 0 | 2 | 2 | 3 | 1 | 2 | 10 | | 0.02 | | Bacteroidetes |
| 2553 | 1 | | 0 | 0 | 2 | 0 | 3 | 2 | 2 | 10 | | 0.02 | | Firmicutes;Clostridia;Clostridiales;Lachnospiraceae |
| 16235 | 0 | | 2 | 0 | 2 | 0 | 2 | 2 | 2 | 10 | | 0.02 | | Firmicutes;Clostridia;Clostridiales;Ruminococcaceae |
| 5449 | 1 | | 3 | 0 | 2 | 2 | 1 | 0 | 1 | 10 | | 0.02 | | Firmicutes;Clostridia;Clostridiales;Ruminococcaceae |
| 11629 | 0 | | 2 | 1 | 2 | 1 | 2 | 0 | 2 | 10 | | 0.02 | | Bacteria |
| 7495 | 0 | | 1 | 2 | 2 | 0 | 2 | 1 | 2 | 10 | | 0.02 | | Firmicutes;Clostridia;Clostridiales;Lachnospiraceae |
| 13303 | 1 | | 0 | 3 | 2 | 0 | 1 | 2 | 1 | 10 | | 0.02 | | Firmicutes;Clostridia;Clostridiales |
| 3040 | 2 | | 3 | 3 | 2 | 0 | 0 | 0 | 0 | 10 | | 0.02 | | Bacteroidetes |
| 6417 | 1 | | 1 | 4 | 2 | 1 | 0 | 1 | 0 | 10 | | 0.02 | | Firmicutes;Clostridia;Clostridiales |
| 18818 | 1 | | 2 | 5 | 2 | 0 | 0 | 0 | 0 | 10 | | 0.02 | | Bacteroidetes |
| 19449 | 1 | | 0 | 0 | 3 | 0 | 4 | 2 | 0 | 10 | | 0.02 | | Firmicutes;Clostridia;Clostridiales;Lachnospiraceae |
| 3793 | 0 | | 1 | 0 | 3 | 2 | 1 | 0 | 3 | 10 | | 0.02 | | Bacteroidetes |
| 19174 | 1 | | 2 | 0 | 3 | 3 | 0 | 1 | 0 | 10 | | 0.02 | | Firmicutes;Clostridia;Clostridiales;Incertae Sedis XIII;Anaerovorax |
| 8033 | 0 | | 1 | 1 | 3 | 0 | 3 | 1 | 1 | 10 | | 0.02 | | Firmicutes;Clostridia;Clostridiales;Ruminococcaceae;Ruminococcus |
| 303 | 4 | | 1 | 1 | 3 | 0 | 0 | 1 | 0 | 10 | | 0.02 | | Bacteroidetes;Bacteroidetes;Bacteroidales;Prevotellaceae |
| 5457 | 0 | | 2 | 1 | 3 | 0 | 0 | 1 | 3 | 10 | | 0.02 | | Firmicutes;Clostridia;Clostridiales;Lachnospiraceae |
| 5016 | 2 | | 1 | 2 | 3 | 1 | 0 | 0 | 1 | 10 | | 0.02 | | Bacteroidetes;Bacteroidetes;Bacteroidales;Prevotellaceae |
| 1414 | 1 | | 4 | 2 | 3 | 0 | 0 | 0 | 0 | 10 | | 0.02 | | TM7;TM7_genera_incertae_sedis |
| 7896 | 1 | | 1 | 5 | 3 | 0 | 0 | 0 | 0 | 10 | | 0.02 | | Bacteroidetes;Bacteroidetes;Bacteroidales;Porphyromonadaceae |
| 1081 | 2 | | 2 | 0 | 4 | 0 | 2 | 0 | 0 | 10 | | 0.02 | | Firmicutes;Erysipelotrichi;Erysipelotrichales;Erysipelotrichaceae;Bulleidia |
| 23673 | 0 | | 4 | 1 | 4 | 0 | 1 | 0 | 0 | 10 | | 0.02 | | Firmicutes;Clostridia;Clostridiales;Ruminococcaceae |
| 23538 | 0 | | 1 | 0 | 5 | 0 | 2 | 1 | 1 | 10 | | 0.02 | | Firmicutes;Clostridia;Clostridiales;Ruminococcaceae |
| 20492 | 0 | | 0 | 1 | 5 | 0 | 2 | 1 | 1 | 10 | | 0.02 | | Firmicutes;Clostridia;Clostridiales;Ruminococcaceae |
| 12058 | 0 | | 0 | 0 | 0 | 4 | 3 | 1 | 1 | 9 | | 0.02 | | Firmicutes;Clostridia;Clostridiales;Ruminococcaceae |
| 7497 | 0 | | 0 | 0 | 0 | 5 | 1 | 2 | 1 | 9 | | 0.02 | | Bacteria |
| 3319 | 0 | | 0 | 0 | 0 | 1 | 5 | 2 | 1 | 9 | | 0.02 | | Firmicutes |
| 10638 | 0 | | 0 | 0 | 0 | 2 | 3 | 3 | 1 | 9 | | 0.02 | | Bacteroidetes;Bacteroidetes;Bacteroidales;Prevotellaceae |
| 1717 | 0 | | 0 | 0 | 0 | 2 | 2 | 3 | 2 | 9 | | 0.02 | | Bacteroidetes;Bacteroidetes;Bacteroidales;Prevotellaceae;Prevotella |
| 6430 | 0 | | 0 | 0 | 0 | 3 | 2 | 1 | 3 | 9 | | 0.02 | | Bacteroidetes;Bacteroidetes;Bacteroidales;Prevotellaceae;Prevotella |
| 8739 | 0 | | 0 | 0 | 0 | 1 | 3 | 2 | 3 | 9 | | 0.02 | | Bacteroidetes;Bacteroidetes;Bacteroidales;Prevotellaceae;Prevotella |
| 17809 | 0 | | 0 | 0 | 0 | 1 | 2 | 3 | 3 | 9 | | 0.02 | | Bacteroidetes;Bacteroidetes;Bacteroidales |
| 15534 | 0 | | 0 | 0 | 0 | 2 | 2 | 1 | 4 | 9 | | 0.02 | | Firmicutes;Clostridia;Clostridiales |
| 11071 | 0 | | 0 | 0 | 0 | 1 | 2 | 2 | 4 | 9 | | 0.02 | | Bacteria |
| 23504 | 0 | | 0 | 0 | 0 | 1 | 1 | 3 | 4 | 9 | | 0.02 | | Bacteroidetes;Bacteroidetes;Bacteroidales;Prevotellaceae;Prevotella |
| 17897 | 1 | | 0 | 0 | 0 | 1 | 2 | 2 | 3 | 9 | | 0.02 | | Bacteria |
| 15438 | 3 | | 0 | 0 | 0 | 1 | 3 | 0 | 2 | 9 | | 0.02 | | Firmicutes;Clostridia;Clostridiales;Lachnospiraceae |
| 12138 | 0 | | 1 | 0 | 0 | 2 | 3 | 1 | 2 | 9 | | 0.02 | | Bacteroidetes;Bacteroidetes;Bacteroidales;Prevotellaceae;Prevotella |
| 16463 | 0 | | 1 | 0 | 0 | 2 | 3 | 1 | 2 | 9 | | 0.02 | | Bacteroidetes;Bacteroidetes;Bacteroidales;Prevotellaceae;Prevotella |
| 18209 | 0 | | 1 | 0 | 0 | 0 | 2 | 4 | 2 | 9 | | 0.02 | | Firmicutes;Clostridia;Clostridiales;Lachnospiraceae |
| 2501 | 0 | | 1 | 0 | 0 | 2 | 1 | 1 | 4 | 9 | | 0.02 | | Bacteria |
| 3626 | 2 | | 1 | 0 | 0 | 1 | 2 | 1 | 2 | 9 | | 0.02 | | Bacteria |
| 8457 | 0 | | 2 | 0 | 0 | 2 | 3 | 0 | 2 | 9 | | 0.02 | | TM7;TM7_genera_incertae_sedis |
| 6072 | 1 | | 0 | 1 | 0 | 0 | 1 | 3 | 3 | 9 | | 0.02 | | TM7;TM7_genera_incertae_sedis |
| 13637 | 0 | | 2 | 1 | 0 | 2 | 1 | 1 | 2 | 9 | | 0.02 | | Firmicutes;Clostridia |
| 16395 | 0 | | 0 | 2 | 0 | 3 | 1 | 1 | 2 | 9 | | 0.02 | | Bacteroidetes;Bacteroidetes;Bacteroidales;Prevotellaceae |
| 5002 | 0 | | 2 | 3 | 0 | 2 | 1 | 0 | 1 | 9 | | 0.02 | | Firmicutes;Clostridia;Clostridiales |
| 16729 | 0 | | 0 | 0 | 1 | 3 | 3 | 1 | 1 | 9 | | 0.02 | | Bacteroidetes;Bacteroidetes;Bacteroidales;Prevotellaceae;Prevotella |
| 19205 | 0 | | 1 | 0 | 1 | 0 | 4 | 2 | 1 | 9 | | 0.02 | | Firmicutes;Clostridia;Clostridiales;Ruminococcaceae |
| 3182 | 0 | | 1 | 0 | 1 | 1 | 0 | 2 | 4 | 9 | | 0.02 | | Firmicutes;Clostridia;Clostridiales;Ruminococcaceae;Anaerotruncus |
| 6231 | 1 | | 1 | 0 | 1 | 0 | 3 | 1 | 2 | 9 | | 0.02 | | Bacteria |
| 18921 | 3 | | 1 | 0 | 1 | 1 | 0 | 2 | 1 | 9 | | 0.02 | | Bacteroidetes;Bacteroidetes;Bacteroidales;Prevotellaceae;Prevotella |
| 11098 | 0 | | 0 | 1 | 1 | 1 | 3 | 2 | 1 | 9 | | 0.02 | | Bacteroidetes;Bacteroidetes;Bacteroidales |
| 16815 | 1 | | 0 | 1 | 1 | 2 | 3 | 1 | 0 | 9 | | 0.02 | | Firmicutes;Clostridia;Clostridiales;Lachnospiraceae |
| 19219 | 3 | | 0 | 1 | 1 | 1 | 1 | 0 | 2 | 9 | | 0.02 | | Firmicutes;Clostridia;Clostridiales;Ruminococcaceae |
| 10666 | 0 | | 1 | 1 | 1 | 3 | 0 | 3 | 0 | 9 | | 0.02 | | Bacteria |
| 1934 | 0 | | 1 | 1 | 1 | 1 | 1 | 2 | 2 | 9 | | 0.02 | | Actinobacteria;Actinobacteria;Coriobacteridae;Coriobacteriales;Coriobacterineae;Coriobacteriaceae;Eggerthella |
| 21292 | 1 | | 1 | 1 | 1 | 0 | 2 | 2 | 1 | 9 | | 0.02 | | Bacteria |
| 17227 | 2 | | 1 | 1 | 1 | 1 | 0 | 1 | 2 | 9 | | 0.02 | | Chloroflexi;Anaerolineae |
| 16448 | 0 | | 2 | 1 | 1 | 1 | 2 | 1 | 1 | 9 | | 0.02 | | Firmicutes;Clostridia;Clostridiales |
| 20251 | 0 | | 3 | 1 | 1 | 1 | 3 | 0 | 0 | 9 | | 0.02 | | Bacteroidetes |
| 12360 | 1 | | 3 | 1 | 1 | 1 | 1 | 0 | 1 | 9 | | 0.02 | | Bacteria |
| 5833 | 1 | | 0 | 2 | 1 | 1 | 1 | 1 | 2 | 9 | | 0.02 | | Firmicutes;Clostridia |
| 20616 | 1 | | 1 | 2 | 1 | 1 | 1 | 1 | 1 | 9 | | 0.02 | | Bacteroidetes;Bacteroidetes;Bacteroidales |
| 9632 | 3 | | 1 | 2 | 1 | 1 | 0 | 0 | 1 | 9 | | 0.02 | | Firmicutes;Clostridia;Clostridiales |
| 6962 | 0 | | 1 | 3 | 1 | 1 | 0 | 1 | 2 | 9 | | 0.02 | | Bacteroidetes |
| 13087 | 1 | | 1 | 4 | 1 | 1 | 0 | 1 | 0 | 9 | | 0.02 | | Firmicutes;Clostridia;Clostridiales |
| 1135 | 2 | | 1 | 0 | 2 | 1 | 1 | 2 | 0 | 9 | | 0.02 | | Bacteroidetes |
| 6512 | 3 | | 0 | 1 | 2 | 0 | 1 | 1 | 1 | 9 | | 0.02 | | Firmicutes;Clostridia;Clostridiales;Lachnospiraceae |
| 12892 | 3 | | 1 | 1 | 2 | 0 | 1 | 0 | 1 | 9 | | 0.02 | | Planctomycetes;Planctomycetacia;Planctomycetales;Planctomycetaceae |
| 17966 | 1 | | 2 | 1 | 2 | 1 | 0 | 2 | 0 | 9 | | 0.02 | | Firmicutes;Clostridia;Clostridiales;Lachnospiraceae |
| 7452 | 0 | | 3 | 2 | 2 | 0 | 2 | 0 | 0 | 9 | | 0.02 | | Firmicutes;Clostridia;Clostridiales |
| 12473 | 0 | | 4 | 2 | 2 | 0 | 1 | 0 | 0 | 9 | | 0.02 | | Firmicutes;Clostridia;Clostridiales |
| 8028 | 0 | | 0 | 3 | 2 | 1 | 2 | 1 | 0 | 9 | | 0.02 | | Firmicutes;Clostridia;Clostridiales;Incertae Sedis XIII |
| 1287 | 0 | | 1 | 0 | 3 | 2 | 1 | 2 | 0 | 9 | | 0.02 | | Firmicutes;Clostridia;Clostridiales;Lachnospiraceae |
| 12971 | 1 | | 3 | 0 | 3 | 2 | 0 | 0 | 0 | 9 | | 0.02 | | Firmicutes;Clostridia;Clostridiales;Lachnospiraceae |
| 4500 | 2 | | 2 | 1 | 3 | 1 | 0 | 0 | 0 | 9 | | 0.02 | | Firmicutes;Clostridia;Clostridiales;Ruminococcaceae;Ruminococcus |
| 11398 | 0 | | 2 | 3 | 3 | 1 | 0 | 0 | 0 | 9 | | 0.02 | | Firmicutes;Clostridia;Clostridiales |
| 10935 | 1 | | 0 | 4 | 3 | 0 | 0 | 0 | 1 | 9 | | 0.02 | | Firmicutes;Clostridia;Clostridiales;Lachnospiraceae |
| 16055 | 1 | | 2 | 1 | 4 | 0 | 0 | 0 | 1 | 9 | | 0.02 | | Firmicutes;Clostridia;Clostridiales;Incertae Sedis XIII;Anaerovorax |
| 23718 | 1 | | 1 | 2 | 4 | 0 | 0 | 1 | 0 | 9 | | 0.02 | | Firmicutes;Clostridia;Clostridiales |
| 22650 | 2 | | 1 | 2 | 4 | 0 | 0 | 0 | 0 | 9 | | 0.02 | | Bacteroidetes |
| 11181 | 0 | | 1 | 3 | 4 | 1 | 0 | 0 | 0 | 9 | | 0.02 | | Firmicutes;Clostridia;Clostridiales;Ruminococcaceae |
| 2030 | 1 | | 1 | 1 | 5 | 0 | 0 | 0 | 1 | 9 | | 0.02 | | Firmicutes;Clostridia;Clostridiales |
| 8934 | 1 | | 0 | 2 | 5 | 0 | 0 | 1 | 0 | 9 | | 0.02 | | TM7;TM7_genera_incertae_sedis |
| 7517 | 0 | | 0 | 0 | 0 | 2 | 4 | 1 | 1 | 8 | | 0.02 | | Firmicutes;Clostridia |
| 22882 | 0 | | 0 | 0 | 0 | 1 | 4 | 2 | 1 | 8 | | 0.02 | | Bacteroidetes;Bacteroidetes;Bacteroidales;Prevotellaceae;Prevotella |
| 9347 | 0 | | 0 | 0 | 0 | 2 | 2 | 3 | 1 | 8 | | 0.02 | | Bacteroidetes;Bacteroidetes;Bacteroidales;Prevotellaceae;Prevotella |
| 2791 | 0 | | 0 | 0 | 0 | 3 | 2 | 1 | 2 | 8 | | 0.02 | | Bacteria |
| 23328 | 0 | | 0 | 0 | 0 | 3 | 2 | 1 | 2 | 8 | | 0.02 | | Bacteria |
| 12097 | 0 | | 0 | 0 | 0 | 3 | 1 | 2 | 2 | 8 | | 0.02 | | Bacteroidetes;Bacteroidetes;Bacteroidales;Prevotellaceae;Hallella |
| 21573 | 0 | | 0 | 0 | 0 | 3 | 1 | 2 | 2 | 8 | | 0.02 | | Firmicutes |
| 23391 | 0 | | 0 | 0 | 0 | 2 | 2 | 1 | 3 | 8 | | 0.02 | | Bacteroidetes |
| 18179 | 0 | | 0 | 0 | 0 | 2 | 1 | 2 | 3 | 8 | | 0.02 | | Bacteroidetes;Bacteroidetes;Bacteroidales;Prevotellaceae |
| 15824 | 0 | | 0 | 0 | 0 | 1 | 1 | 3 | 3 | 8 | | 0.02 | | TM7;TM7_genera_incertae_sedis |
| 5484 | 0 | | 0 | 0 | 0 | 1 | 2 | 1 | 4 | 8 | | 0.02 | | Bacteroidetes;Bacteroidetes;Bacteroidales |
| 19640 | 0 | | 0 | 0 | 0 | 1 | 1 | 1 | 5 | 8 | | 0.02 | | Bacteroidetes;Bacteroidetes;Bacteroidales;Prevotellaceae;Prevotella |
| 4244 | 1 | | 0 | 0 | 0 | 3 | 2 | 2 | 0 | 8 | | 0.02 | | Bacteria |
| 6541 | 0 | | 1 | 0 | 0 | 1 | 2 | 2 | 2 | 8 | | 0.02 | | Bacteroidetes;Bacteroidetes;Bacteroidales;Prevotellaceae;Prevotella |
| 23034 | 0 | | 1 | 0 | 0 | 1 | 2 | 2 | 2 | 8 | | 0.02 | | Bacteroidetes |
| 22971 | 0 | | 1 | 0 | 0 | 1 | 2 | 1 | 3 | 8 | | 0.02 | | Bacteria |
| 21830 | 0 | | 1 | 0 | 0 | 0 | 1 | 3 | 3 | 8 | | 0.02 | | Bacteroidetes;Bacteroidetes;Bacteroidales;Prevotellaceae;Prevotella |
| 21838 | 0 | | 1 | 0 | 0 | 0 | 1 | 3 | 3 | 8 | | 0.02 | | Bacteroidetes;Bacteroidetes;Bacteroidales;Prevotellaceae |
| 16316 | 1 | | 1 | 0 | 0 | 0 | 3 | 2 | 1 | 8 | | 0.02 | | Bacteroidetes;Bacteroidetes;Bacteroidales |
| 23102 | 1 | | 1 | 0 | 0 | 0 | 3 | 1 | 2 | 8 | | 0.02 | | Bacteria |
| 9646 | 1 | | 1 | 0 | 0 | 0 | 1 | 2 | 3 | 8 | | 0.02 | | Firmicutes;Clostridia;Clostridiales;Ruminococcaceae;Sporobacter |
| 7773 | 0 | | 2 | 0 | 0 | 3 | 2 | 0 | 1 | 8 | | 0.02 | | Firmicutes;Clostridia;Clostridiales;Ruminococcaceae |
| 15588 | 0 | | 0 | 1 | 0 | 3 | 2 | 1 | 1 | 8 | | 0.02 | | Bacteria |
| 295 | 0 | | 0 | 1 | 0 | 4 | 0 | 1 | 2 | 8 | | 0.02 | | Bacteroidetes;Bacteroidetes;Bacteroidales |
| 22020 | 0 | | 1 | 1 | 0 | 0 | 3 | 1 | 2 | 8 | | 0.02 | | Firmicutes;Clostridia |
| 13404 | 1 | | 2 | 1 | 0 | 2 | 1 | 1 | 0 | 8 | | 0.02 | | Firmicutes;Clostridia;Clostridiales |
| 1500 | 0 | | 3 | 1 | 0 | 1 | 0 | 0 | 3 | 8 | | 0.02 | | Bacteroidetes;Bacteroidetes;Bacteroidales |
| 17212 | 1 | | 0 | 2 | 0 | 0 | 2 | 3 | 0 | 8 | | 0.02 | | Bacteroidetes |
| 9274 | 1 | | 1 | 2 | 0 | 2 | 1 | 1 | 0 | 8 | | 0.02 | | Firmicutes;Clostridia;Clostridiales;Ruminococcaceae;Sporobacter |
| 10313 | 0 | | 3 | 2 | 0 | 0 | 0 | 1 | 2 | 8 | | 0.02 | | Firmicutes;Clostridia;Clostridiales;Ruminococcaceae |
| 21832 | 0 | | 0 | 0 | 1 | 3 | 1 | 2 | 1 | 8 | | 0.02 | | Bacteroidetes;Bacteroidetes;Bacteroidales;Rikenellaceae |
| 3825 | 0 | | 0 | 0 | 1 | 2 | 3 | 0 | 2 | 8 | | 0.02 | | Bacteroidetes;Bacteroidetes;Bacteroidales;Prevotellaceae |
| 13366 | 0 | | 0 | 0 | 1 | 2 | 1 | 2 | 2 | 8 | | 0.02 | | Bacteroidetes |
| 10975 | 1 | | 0 | 0 | 1 | 0 | 3 | 1 | 2 | 8 | | 0.02 | | Bacteria |
| 9890 | 1 | | 0 | 0 | 1 | 1 | 1 | 2 | 2 | 8 | | 0.02 | | Firmicutes;Clostridia;Clostridiales |
| 21479 | 1 | | 0 | 0 | 1 | 0 | 1 | 3 | 2 | 8 | | 0.02 | | Bacteroidetes;Bacteroidetes;Bacteroidales |
| 22292 | 2 | | 0 | 0 | 1 | 0 | 1 | 1 | 3 | 8 | | 0.02 | | Firmicutes;Clostridia |
| 18368 | 0 | | 1 | 0 | 1 | 2 | 2 | 1 | 1 | 8 | | 0.02 | | Firmicutes;Clostridia;Clostridiales;Ruminococcaceae |
| 21598 | 0 | | 1 | 0 | 1 | 1 | 3 | 0 | 2 | 8 | | 0.02 | | Bacteria |
| 13948 | 1 | | 1 | 0 | 1 | 1 | 0 | 1 | 3 | 8 | | 0.02 | | Bacteria |
| 10402 | 0 | | 2 | 0 | 1 | 2 | 0 | 3 | 0 | 8 | | 0.02 | | Bacteroidetes;Bacteroidetes;Bacteroidales |
| 14146 | 0 | | 2 | 0 | 1 | 0 | 1 | 0 | 4 | 8 | | 0.02 | | Bacteria |
| 19843 | 1 | | 2 | 0 | 1 | 0 | 0 | 2 | 2 | 8 | | 0.02 | | Firmicutes;Clostridia;Clostridiales |
| 11814 | 0 | | 3 | 0 | 1 | 1 | 2 | 1 | 0 | 8 | | 0.02 | | Firmicutes;Clostridia;Clostridiales |
| 4902 | 0 | | 3 | 0 | 1 | 0 | 1 | 0 | 3 | 8 | | 0.02 | | Firmicutes;Clostridia |
| 13270 | 1 | | 3 | 0 | 1 | 0 | 0 | 1 | 2 | 8 | | 0.02 | | Bacteria |
| 7027 | 0 | | 0 | 1 | 1 | 1 | 0 | 4 | 1 | 8 | | 0.02 | | Firmicutes;Clostridia;Clostridiales;Lachnospiraceae |
| 11164 | 1 | | 0 | 1 | 1 | 1 | 1 | 2 | 1 | 8 | | 0.02 | | Bacteria |
| 18351 | 0 | | 1 | 1 | 1 | 1 | 0 | 2 | 2 | 8 | | 0.02 | | Firmicutes;Clostridia |
| 12335 | 1 | | 1 | 1 | 1 | 0 | 0 | 3 | 1 | 8 | | 0.02 | | Firmicutes;Clostridia;Clostridiales;Ruminococcaceae |
| 15866 | 0 | | 3 | 1 | 1 | 1 | 1 | 0 | 1 | 8 | | 0.02 | | TM7;TM7_genera_incertae_sedis |
| 5666 | 1 | | 5 | 1 | 1 | 0 | 0 | 0 | 0 | 8 | | 0.02 | | Bacteroidetes;Bacteroidetes;Bacteroidales;Prevotellaceae |
| 12613 | 1 | | 5 | 1 | 1 | 0 | 0 | 0 | 0 | 8 | | 0.02 | | Bacteroidetes;Bacteroidetes;Bacteroidales;Prevotellaceae |
| 9564 | 0 | | 0 | 2 | 1 | 0 | 3 | 2 | 0 | 8 | | 0.02 | | Bacteria |
| 6768 | 0 | | 1 | 2 | 1 | 2 | 1 | 0 | 1 | 8 | | 0.02 | | Firmicutes;Clostridia;Clostridiales |
| 19316 | 0 | | 1 | 2 | 1 | 0 | 0 | 1 | 3 | 8 | | 0.02 | | Firmicutes;Clostridia;Clostridiales;Lachnospiraceae |
| 3185 | 1 | | 1 | 2 | 1 | 0 | 2 | 0 | 1 | 8 | | 0.02 | | Firmicutes;Clostridia;Clostridiales;Ruminococcaceae |
| 3855 | 0 | | 2 | 2 | 1 | 0 | 1 | 1 | 1 | 8 | | 0.02 | | Bacteroidetes;Bacteroidetes;Bacteroidales |
| 20398 | 0 | | 0 | 3 | 1 | 1 | 3 | 0 | 0 | 8 | | 0.02 | | Firmicutes;Clostridia;Clostridiales |
| 4876 | 0 | | 2 | 3 | 1 | 0 | 0 | 1 | 1 | 8 | | 0.02 | | Firmicutes;Clostridia;Clostridiales;Lachnospiraceae |
| 11354 | 1 | | 0 | 0 | 2 | 2 | 1 | 2 | 0 | 8 | | 0.02 | | Bacteroidetes |
| 13979 | 0 | | 1 | 0 | 2 | 2 | 2 | 0 | 1 | 8 | | 0.02 | | Firmicutes;Clostridia;Clostridiales;Lachnospiraceae |
| 6552 | 1 | | 1 | 0 | 2 | 0 | 1 | 2 | 1 | 8 | | 0.02 | | Firmicutes;Clostridia;Clostridiales;Ruminococcaceae |
| 6721 | 1 | | 1 | 0 | 2 | 0 | 2 | 0 | 2 | 8 | | 0.02 | | Bacteroidetes;Bacteroidetes;Bacteroidales |
| 18762 | 1 | | 2 | 0 | 2 | 1 | 1 | 1 | 0 | 8 | | 0.02 | | Firmicutes;Clostridia;Clostridiales;Ruminococcaceae |
| 431 | 0 | | 0 | 1 | 2 | 0 | 2 | 2 | 1 | 8 | | 0.02 | | Bacteria |
| 18592 | 2 | | 0 | 1 | 2 | 2 | 1 | 0 | 0 | 8 | | 0.02 | | Bacteroidetes |
| 10146 | 2 | | 0 | 1 | 2 | 2 | 0 | 1 | 0 | 8 | | 0.02 | | Firmicutes;Clostridia;Clostridiales;Lachnospiraceae |
| 21091 | 0 | | 1 | 1 | 2 | 1 | 0 | 2 | 1 | 8 | | 0.02 | | Firmicutes;Clostridia;Clostridiales;Ruminococcaceae |
| 1563 | 1 | | 1 | 1 | 2 | 1 | 0 | 2 | 0 | 8 | | 0.02 | | Firmicutes;Clostridia;Clostridiales;Ruminococcaceae |
| 15716 | 2 | | 1 | 1 | 2 | 0 | 0 | 1 | 1 | 8 | | 0.02 | | Firmicutes;Clostridia;Clostridiales |
| 20283 | 4 | | 1 | 1 | 2 | 0 | 0 | 0 | 0 | 8 | | 0.02 | | Firmicutes;Clostridia;Clostridiales |
| 5212 | 0 | | 2 | 1 | 2 | 0 | 1 | 1 | 1 | 8 | | 0.02 | | Firmicutes;Clostridia;Clostridiales;Ruminococcaceae |
| 7552 | 1 | | 4 | 1 | 2 | 0 | 0 | 0 | 0 | 8 | | 0.02 | | Firmicutes;Clostridia;Clostridiales;Ruminococcaceae |
| 17024 | 1 | | 4 | 1 | 2 | 0 | 0 | 0 | 0 | 8 | | 0.02 | | Bacteroidetes;Bacteroidetes;Bacteroidales;Prevotellaceae |
| 4333 | 1 | | 0 | 2 | 2 | 0 | 0 | 1 | 2 | 8 | | 0.02 | | Bacteroidetes |
| 8945 | 1 | | 1 | 2 | 2 | 0 | 1 | 0 | 1 | 8 | | 0.02 | | Firmicutes;Clostridia;Clostridiales |
| 14595 | 2 | | 1 | 2 | 2 | 0 | 1 | 0 | 0 | 8 | | 0.02 | | Bacteria |
| 19898 | 1 | | 1 | 3 | 2 | 0 | 0 | 1 | 0 | 8 | | 0.02 | | Bacteroidetes |
| 2933 | 2 | | 1 | 3 | 2 | 0 | 0 | 0 | 0 | 8 | | 0.02 | | Bacteroidetes;Bacteroidetes;Bacteroidales;Prevotellaceae |
| 12938 | 2 | | 1 | 3 | 2 | 0 | 0 | 0 | 0 | 8 | | 0.02 | | Bacteroidetes;Bacteroidetes;Bacteroidales;Prevotellaceae;Hallella |
| 17886 | 0 | | 2 | 3 | 2 | 0 | 1 | 0 | 0 | 8 | | 0.02 | | Bacteroidetes |
| 10782 | 1 | | 2 | 3 | 2 | 0 | 0 | 0 | 0 | 8 | | 0.02 | | Bacteroidetes;Bacteroidetes;Bacteroidales |
| 12284 | 1 | | 1 | 0 | 3 | 0 | 1 | 1 | 1 | 8 | | 0.02 | | Firmicutes;Clostridia;Clostridiales;Ruminococcaceae;Ruminococcus |
| 15431 | 0 | | 0 | 1 | 3 | 2 | 1 | 1 | 0 | 8 | | 0.02 | | Chloroflexi;Anaerolineae |
| 18380 | 1 | | 0 | 1 | 3 | 0 | 2 | 0 | 1 | 8 | | 0.02 | | Firmicutes;Clostridia;Clostridiales;Ruminococcaceae |
| 20036 | 1 | | 2 | 2 | 3 | 0 | 0 | 0 | 0 | 8 | | 0.02 | | Bacteroidetes |
| 8292 | 1 | | 1 | 0 | 5 | 0 | 0 | 0 | 1 | 8 | | 0.02 | | Bacteria |
| 6100 | 0 | | 0 | 0 | 0 | 3 | 1 | 2 | 1 | 7 | | 0.01 | | Bacteria |
| 7577 | 0 | | 0 | 0 | 0 | 1 | 3 | 2 | 1 | 7 | | 0.01 | | Bacteroidetes;Bacteroidetes;Bacteroidales;Prevotellaceae;Prevotella |
| 4921 | 0 | | 0 | 0 | 0 | 3 | 1 | 1 | 2 | 7 | | 0.01 | | Bacteroidetes;Bacteroidetes;Bacteroidales;Prevotellaceae;Prevotella |
| 10197 | 0 | | 0 | 0 | 0 | 2 | 1 | 2 | 2 | 7 | | 0.01 | | Firmicutes;Clostridia;Clostridiales;Ruminococcaceae;Ruminococcus |
| 17687 | 0 | | 0 | 0 | 0 | 1 | 2 | 2 | 2 | 7 | | 0.01 | | Firmicutes;Clostridia;Clostridiales;Ruminococcaceae |
| 18297 | 0 | | 0 | 0 | 0 | 1 | 2 | 2 | 2 | 7 | | 0.01 | | Bacteroidetes;Bacteroidetes;Bacteroidales;Prevotellaceae |
| 18048 | 0 | | 0 | 0 | 0 | 2 | 1 | 1 | 3 | 7 | | 0.01 | | Firmicutes;Clostridia;Clostridiales |
| 19323 | 0 | | 0 | 0 | 0 | 1 | 1 | 2 | 3 | 7 | | 0.01 | | Bacteroidetes;Bacteroidetes;Bacteroidales |
| 10867 | 0 | | 0 | 0 | 0 | 1 | 1 | 1 | 4 | 7 | | 0.01 | | Bacteria |
| 202 | 1 | | 0 | 0 | 0 | 1 | 3 | 1 | 1 | 7 | | 0.01 | | Bacteroidetes |
| 9559 | 1 | | 0 | 0 | 0 | 1 | 3 | 1 | 1 | 7 | | 0.01 | | Firmicutes;Clostridia;Clostridiales;Ruminococcaceae |
| 14522 | 1 | | 0 | 0 | 0 | 1 | 2 | 1 | 2 | 7 | | 0.01 | | Bacteroidetes;Bacteroidetes;Bacteroidales |
| 21470 | 0 | | 1 | 0 | 0 | 2 | 2 | 2 | 0 | 7 | | 0.01 | | Firmicutes;Clostridia;Clostridiales;Lachnospiraceae |
| 968 | 0 | | 1 | 0 | 0 | 2 | 3 | 0 | 1 | 7 | | 0.01 | | Bacteroidetes;Bacteroidetes;Bacteroidales;Prevotellaceae;Prevotella |
| 418 | 0 | | 1 | 0 | 0 | 3 | 0 | 2 | 1 | 7 | | 0.01 | | Firmicutes;Clostridia;Clostridiales;Ruminococcaceae |
| 8566 | 0 | | 1 | 0 | 0 | 1 | 2 | 2 | 1 | 7 | | 0.01 | | Bacteroidetes;Bacteroidetes;Bacteroidales;Prevotellaceae |
| 4139 | 0 | | 1 | 0 | 0 | 1 | 1 | 3 | 1 | 7 | | 0.01 | | Bacteria |
| 719 | 0 | | 1 | 0 | 0 | 1 | 1 | 2 | 2 | 7 | | 0.01 | | Firmicutes;Clostridia;Clostridiales |
| 15423 | 1 | | 1 | 0 | 0 | 3 | 0 | 0 | 2 | 7 | | 0.01 | | Firmicutes;Clostridia;Clostridiales |
| 23530 | 1 | | 1 | 0 | 0 | 1 | 2 | 0 | 2 | 7 | | 0.01 | | Firmicutes;Clostridia;Clostridiales;Lachnospiraceae |
| 19483 | 2 | | 1 | 0 | 0 | 0 | 1 | 0 | 3 | 7 | | 0.01 | | Bacteroidetes;Bacteroidetes;Bacteroidales |
| 16814 | 0 | | 2 | 0 | 0 | 0 | 2 | 2 | 1 | 7 | | 0.01 | | Firmicutes;Clostridia;Clostridiales;Ruminococcaceae |
| 617 | 0 | | 2 | 0 | 0 | 1 | 1 | 1 | 2 | 7 | | 0.01 | | Bacteroidetes;Bacteroidetes;Bacteroidales;Prevotellaceae |
| 1248 | 0 | | 0 | 1 | 0 | 4 | 1 | 0 | 1 | 7 | | 0.01 | | Bacteroidetes;Bacteroidetes;Bacteroidales;Prevotellaceae |
| 11141 | 0 | | 0 | 1 | 0 | 2 | 1 | 2 | 1 | 7 | | 0.01 | | Bacteroidetes;Bacteroidetes;Bacteroidales;Prevotellaceae;Prevotella |
| 16066 | 0 | | 0 | 1 | 0 | 1 | 1 | 3 | 1 | 7 | | 0.01 | | Bacteroidetes;Bacteroidetes;Bacteroidales |
| 815 | 0 | | 0 | 1 | 0 | 1 | 2 | 1 | 2 | 7 | | 0.01 | | Firmicutes;Clostridia;Clostridiales;Ruminococcaceae |
| 8107 | 0 | | 0 | 1 | 0 | 0 | 1 | 2 | 3 | 7 | | 0.01 | | Firmicutes;Clostridia |
| 18276 | 1 | | 0 | 1 | 0 | 1 | 2 | 2 | 0 | 7 | | 0.01 | | Firmicutes;Clostridia;Clostridiales;Lachnospiraceae |
| 14642 | 1 | | 0 | 1 | 0 | 1 | 1 | 2 | 1 | 7 | | 0.01 | | Firmicutes;Clostridia;Clostridiales;Ruminococcaceae |
| 15073 | 0 | | 1 | 1 | 0 | 0 | 2 | 1 | 2 | 7 | | 0.01 | | Firmicutes;Clostridia;Clostridiales;Lachnospiraceae |
| 1560 | 1 | | 2 | 1 | 0 | 0 | 2 | 0 | 1 | 7 | | 0.01 | | Bacteroidetes;Bacteroidetes;Bacteroidales;Prevotellaceae;Hallella |
| 21674 | 2 | | 2 | 1 | 0 | 1 | 0 | 0 | 1 | 7 | | 0.01 | | Firmicutes;Clostridia;Clostridiales;Incertae Sedis XIII;Anaerovorax |
| 9932 | 0 | | 3 | 1 | 0 | 1 | 0 | 2 | 0 | 7 | | 0.01 | | Bacteria |
| 19892 | 0 | | 3 | 1 | 0 | 0 | 0 | 1 | 2 | 7 | | 0.01 | | Bacteria |
| 182 | 0 | | 0 | 2 | 0 | 2 | 2 | 1 | 0 | 7 | | 0.01 | | Firmicutes;Clostridia;Clostridiales;Ruminococcaceae |
| 19637 | 1 | | 0 | 2 | 0 | 0 | 3 | 1 | 0 | 7 | | 0.01 | | Firmicutes;Clostridia;Clostridiales |
| 3308 | 0 | | 1 | 3 | 0 | 0 | 2 | 0 | 1 | 7 | | 0.01 | | Firmicutes;Clostridia;Clostridiales;Ruminococcaceae |
| 7639 | 0 | | 1 | 3 | 0 | 0 | 0 | 1 | 2 | 7 | | 0.01 | | Firmicutes;Clostridia;Clostridiales;Lachnospiraceae |
| 18025 | 0 | | 0 | 0 | 1 | 1 | 2 | 3 | 0 | 7 | | 0.01 | | Bacteroidetes;Bacteroidetes;Bacteroidales |
| 5583 | 0 | | 0 | 0 | 1 | 2 | 1 | 1 | 2 | 7 | | 0.01 | | Firmicutes;Clostridia;Clostridiales;Lachnospiraceae;Butyrivibrio |
| 11863 | 0 | | 0 | 0 | 1 | 1 | 1 | 2 | 2 | 7 | | 0.01 | | Firmicutes;Clostridia;Clostridiales |
| 402 | 1 | | 1 | 0 | 1 | 0 | 2 | 1 | 1 | 7 | | 0.01 | | Firmicutes;Clostridia;Clostridiales;Incertae Sedis XIII;Anaerovorax |
| 16356 | 1 | | 1 | 0 | 1 | 0 | 1 | 1 | 2 | 7 | | 0.01 | | Bacteroidetes;Bacteroidetes;Bacteroidales;Prevotellaceae |
| 5097 | 2 | | 1 | 0 | 1 | 1 | 1 | 1 | 0 | 7 | | 0.01 | | Bacteroidetes;Bacteroidetes;Bacteroidales;Prevotellaceae |
| 12643 | 0 | | 2 | 0 | 1 | 0 | 1 | 2 | 1 | 7 | | 0.01 | | Firmicutes;Clostridia;Clostridiales |
| 12301 | 0 | | 0 | 1 | 1 | 0 | 2 | 2 | 1 | 7 | | 0.01 | | Firmicutes;Clostridia;Clostridiales;Ruminococcaceae |
| 3653 | 1 | | 0 | 1 | 1 | 1 | 2 | 0 | 1 | 7 | | 0.01 | | Firmicutes;Clostridia;Clostridiales;Ruminococcaceae |
| 3991 | 0 | | 1 | 1 | 1 | 0 | 4 | 0 | 0 | 7 | | 0.01 | | Firmicutes;Clostridia;Clostridiales;Lachnospiraceae |
| 14790 | 1 | | 1 | 1 | 1 | 1 | 1 | 0 | 1 | 7 | | 0.01 | | Firmicutes;Clostridia;Clostridiales;Ruminococcaceae |
| 9639 | 2 | | 3 | 1 | 1 | 0 | 0 | 0 | 0 | 7 | | 0.01 | | Bacteroidetes;Bacteroidetes;Bacteroidales;Prevotellaceae |
| 21667 | 0 | | 1 | 2 | 1 | 0 | 0 | 1 | 2 | 7 | | 0.01 | | Firmicutes;Clostridia |
| 12504 | 1 | | 1 | 2 | 1 | 1 | 1 | 0 | 0 | 7 | | 0.01 | | Bacteroidetes;Bacteroidetes;Bacteroidales |
| 18346 | 3 | | 1 | 2 | 1 | 0 | 0 | 0 | 0 | 7 | | 0.01 | | Firmicutes;Clostridia;Clostridiales;Lachnospiraceae |
| 8550 | 1 | | 2 | 3 | 1 | 0 | 0 | 0 | 0 | 7 | | 0.01 | | Bacteroidetes;Bacteroidetes;Bacteroidales |
| 6313 | 1 | | 1 | 4 | 1 | 0 | 0 | 0 | 0 | 7 | | 0.01 | | Bacteroidetes |
| 8803 | 0 | | 0 | 0 | 2 | 1 | 2 | 1 | 1 | 7 | | 0.01 | | Firmicutes;Clostridia;Clostridiales;Ruminococcaceae;Sporobacter |
| 13630 | 0 | | 0 | 0 | 2 | 0 | 2 | 2 | 1 | 7 | | 0.01 | | Bacteroidetes |
| 12903 | 0 | | 0 | 0 | 2 | 0 | 1 | 3 | 1 | 7 | | 0.01 | | Firmicutes;Clostridia;Clostridiales;Ruminococcaceae;Anaerotruncus |
| 15420 | 0 | | 0 | 0 | 2 | 1 | 2 | 0 | 2 | 7 | | 0.01 | | Firmicutes;Clostridia;Clostridiales;Ruminococcaceae |
| 15647 | 0 | | 2 | 0 | 2 | 1 | 1 | 1 | 0 | 7 | | 0.01 | | Firmicutes;Clostridia |
| 17568 | 0 | | 2 | 0 | 2 | 0 | 1 | 0 | 2 | 7 | | 0.01 | | Firmicutes;Clostridia;Clostridiales;Lachnospiraceae |
| 10567 | 1 | | 0 | 1 | 2 | 1 | 1 | 0 | 1 | 7 | | 0.01 | | Bacteria |
| 11606 | 2 | | 0 | 1 | 2 | 0 | 0 | 2 | 0 | 7 | | 0.01 | | Firmicutes;Clostridia;Clostridiales;Lachnospiraceae |
| 2560 | 1 | | 1 | 1 | 2 | 2 | 0 | 0 | 0 | 7 | | 0.01 | | Bacteroidetes;Bacteroidetes;Bacteroidales |
| 15274 | 2 | | 2 | 1 | 2 | 0 | 0 | 0 | 0 | 7 | | 0.01 | | Bacteroidetes;Bacteroidetes;Bacteroidales |
| 12158 | 1 | | 3 | 1 | 2 | 0 | 0 | 0 | 0 | 7 | | 0.01 | | Firmicutes;Clostridia;Clostridiales;Ruminococcaceae |
| 15536 | 1 | | 3 | 1 | 2 | 0 | 0 | 0 | 0 | 7 | | 0.01 | | Bacteroidetes;Bacteroidetes;Bacteroidales;Prevotellaceae |
| 3723 | 0 | | 0 | 2 | 2 | 0 | 0 | 2 | 1 | 7 | | 0.01 | | Firmicutes;Clostridia;Clostridiales;Ruminococcaceae |
| 19431 | 1 | | 0 | 2 | 2 | 0 | 0 | 1 | 1 | 7 | | 0.01 | | Bacteroidetes;Bacteroidetes;Bacteroidales |
| 17202 | 0 | | 1 | 2 | 2 | 0 | 1 | 0 | 1 | 7 | | 0.01 | | Firmicutes;Clostridia;Clostridiales |
| 13743 | 1 | | 1 | 2 | 2 | 0 | 0 | 1 | 0 | 7 | | 0.01 | | Firmicutes;Clostridia;Clostridiales |
| 18779 | 2 | | 1 | 2 | 2 | 0 | 0 | 0 | 0 | 7 | | 0.01 | | Bacteroidetes;Bacteroidetes;Bacteroidales |
| 9545 | 1 | | 1 | 3 | 2 | 0 | 0 | 0 | 0 | 7 | | 0.01 | | Firmicutes;Clostridia;Clostridiales |
| 5945 | 1 | | 1 | 2 | 3 | 0 | 0 | 0 | 0 | 7 | | 0.01 | | Bacteroidetes;Bacteroidetes;Bacteroidales |
| 15620 | 0 | | 0 | 1 | 4 | 1 | 0 | 1 | 0 | 7 | | 0.01 | | Firmicutes;Clostridia;Clostridiales |
| 8547 | 1 | | 1 | 1 | 4 | 0 | 0 | 0 | 0 | 7 | | 0.01 | | Bacteroidetes |
| 2965 | 0 | | 0 | 0 | 0 | 1 | 3 | 1 | 1 | 6 | | 0.01 | | Bacteria |
| 5162 | 0 | | 0 | 0 | 0 | 1 | 3 | 1 | 1 | 6 | | 0.01 | | Bacteroidetes;Bacteroidetes;Bacteroidales;Prevotellaceae;Prevotella |
| 6745 | 0 | | 0 | 0 | 0 | 1 | 3 | 1 | 1 | 6 | | 0.01 | | Bacteroidetes;Bacteroidetes;Bacteroidales;Prevotellaceae;Prevotella |
| 8816 | 0 | | 0 | 0 | 0 | 1 | 1 | 3 | 1 | 6 | | 0.01 | | Bacteroidetes;Bacteroidetes;Bacteroidales;Prevotellaceae;Prevotella |
| 18428 | 0 | | 0 | 0 | 0 | 1 | 1 | 3 | 1 | 6 | | 0.01 | | Firmicutes;Clostridia;Clostridiales;Lachnospiraceae |
| 20107 | 0 | | 0 | 0 | 0 | 1 | 1 | 3 | 1 | 6 | | 0.01 | | Bacteria |
| 5806 | 0 | | 0 | 0 | 0 | 1 | 2 | 1 | 2 | 6 | | 0.01 | | Bacteroidetes |
| 14579 | 0 | | 0 | 0 | 0 | 1 | 2 | 1 | 2 | 6 | | 0.01 | | Firmicutes |
| 20145 | 0 | | 0 | 0 | 0 | 1 | 1 | 2 | 2 | 6 | | 0.01 | | Firmicutes;Clostridia;Clostridiales |
| 425 | 0 | | 0 | 0 | 0 | 1 | 1 | 1 | 3 | 6 | | 0.01 | | Bacteria |
| 7181 | 0 | | 0 | 0 | 0 | 1 | 1 | 1 | 3 | 6 | | 0.01 | | Bacteroidetes;Bacteroidetes;Bacteroidales;Prevotellaceae |
| 7349 | 0 | | 0 | 0 | 0 | 1 | 1 | 1 | 3 | 6 | | 0.01 | | Bacteroidetes;Bacteroidetes;Bacteroidales;Prevotellaceae;Prevotella |
| 8711 | 0 | | 0 | 0 | 0 | 1 | 1 | 1 | 3 | 6 | | 0.01 | | Bacteroidetes;Bacteroidetes;Bacteroidales;Prevotellaceae |
| 9801 | 0 | | 0 | 0 | 0 | 1 | 1 | 1 | 3 | 6 | | 0.01 | | Bacteroidetes;Bacteroidetes;Bacteroidales |
| 19013 | 0 | | 0 | 0 | 0 | 1 | 1 | 1 | 3 | 6 | | 0.01 | | Bacteroidetes;Bacteroidetes;Bacteroidales;Prevotellaceae |
| 22553 | 0 | | 0 | 0 | 0 | 1 | 1 | 1 | 3 | 6 | | 0.01 | | Firmicutes;Clostridia;Clostridiales;Ruminococcaceae |
| 22209 | 1 | | 0 | 0 | 0 | 2 | 1 | 1 | 1 | 6 | | 0.01 | | Firmicutes;Clostridia;Clostridiales;Lachnospiraceae |
| 13246 | 1 | | 0 | 0 | 0 | 1 | 2 | 1 | 1 | 6 | | 0.01 | | Bacteria |
| 16383 | 1 | | 0 | 0 | 0 | 1 | 1 | 2 | 1 | 6 | | 0.01 | | Bacteroidetes |
| 18299 | 1 | | 0 | 0 | 0 | 2 | 1 | 0 | 2 | 6 | | 0.01 | | Bacteroidetes;Bacteroidetes;Bacteroidales;Prevotellaceae;Prevotella |
| 2577 | 2 | | 0 | 0 | 0 | 1 | 2 | 1 | 0 | 6 | | 0.01 | | Bacteroidetes;Bacteroidetes;Bacteroidales |
| 21687 | 2 | | 0 | 0 | 0 | 1 | 2 | 1 | 0 | 6 | | 0.01 | | Bacteroidetes;Bacteroidetes;Bacteroidales |
| 22770 | 2 | | 0 | 0 | 0 | 1 | 2 | 1 | 0 | 6 | | 0.01 | | Bacteroidetes;Bacteroidetes;Bacteroidales;Prevotellaceae |
| 31 | 0 | | 1 | 0 | 0 | 1 | 3 | 0 | 1 | 6 | | 0.01 | | Bacteroidetes |
| 3112 | 0 | | 1 | 0 | 0 | 0 | 1 | 3 | 1 | 6 | | 0.01 | | Bacteroidetes;Bacteroidetes;Bacteroidales |
| 5293 | 0 | | 1 | 0 | 0 | 2 | 0 | 1 | 2 | 6 | | 0.01 | | Bacteroidetes;Bacteroidetes;Bacteroidales;Prevotellaceae;Prevotella |
| 10856 | 1 | | 1 | 0 | 0 | 1 | 0 | 2 | 1 | 6 | | 0.01 | | Bacteroidetes;Bacteroidetes;Bacteroidales |
| 14804 | 1 | | 1 | 0 | 0 | 0 | 1 | 1 | 2 | 6 | | 0.01 | | Bacteroidetes;Bacteroidetes;Bacteroidales;Prevotellaceae;Prevotella |
| 2041 | 2 | | 1 | 0 | 0 | 0 | 0 | 1 | 2 | 6 | | 0.01 | | Firmicutes;Clostridia;Clostridiales |
| 2762 | 1 | | 2 | 0 | 0 | 2 | 0 | 1 | 0 | 6 | | 0.01 | | Firmicutes;Clostridia;Clostridiales;Ruminococcaceae |
| 20824 | 1 | | 2 | 0 | 0 | 1 | 0 | 0 | 2 | 6 | | 0.01 | | Bacteroidetes |
| 439 | 0 | | 0 | 1 | 0 | 2 | 1 | 2 | 0 | 6 | | 0.01 | | Firmicutes;Clostridia;Clostridiales;Ruminococcaceae |
| 2880 | 0 | | 0 | 1 | 0 | 1 | 1 | 3 | 0 | 6 | | 0.01 | | Firmicutes;Clostridia;Clostridiales;Ruminococcaceae |
| 9327 | 0 | | 0 | 1 | 0 | 1 | 1 | 2 | 1 | 6 | | 0.01 | | Firmicutes |
| 5190 | 0 | | 0 | 1 | 0 | 0 | 1 | 3 | 1 | 6 | | 0.01 | | Bacteroidetes;Bacteroidetes;Bacteroidales |
| 2051 | 0 | | 0 | 1 | 0 | 1 | 1 | 1 | 2 | 6 | | 0.01 | | Bacteria |
| 2470 | 0 | | 0 | 1 | 0 | 1 | 0 | 1 | 3 | 6 | | 0.01 | | Firmicutes;Clostridia;Clostridiales;Ruminococcaceae;Sporobacter |
| 6991 | 1 | | 0 | 1 | 0 | 1 | 1 | 1 | 1 | 6 | | 0.01 | | Firmicutes;Clostridia;Clostridiales;Ruminococcaceae |
| 11967 | 1 | | 0 | 1 | 0 | 1 | 1 | 0 | 2 | 6 | | 0.01 | | Bacteroidetes;Bacteroidetes;Bacteroidales;Prevotellaceae |
| 5935 | 0 | | 1 | 1 | 0 | 0 | 0 | 2 | 2 | 6 | | 0.01 | | Bacteroidetes;Bacteroidetes;Bacteroidales |
| 11461 | 1 | | 1 | 1 | 0 | 0 | 2 | 1 | 0 | 6 | | 0.01 | | Firmicutes;Clostridia;Clostridiales;Ruminococcaceae |
| 16851 | 1 | | 1 | 1 | 0 | 0 | 1 | 1 | 1 | 6 | | 0.01 | | Bacteroidetes |
| 17612 | 3 | | 1 | 1 | 0 | 0 | 1 | 0 | 0 | 6 | | 0.01 | | Bacteria |
| 2244 | 0 | | 2 | 1 | 0 | 2 | 0 | 0 | 1 | 6 | | 0.01 | | Bacteroidetes;Bacteroidetes;Bacteroidales;Prevotellaceae;Hallella |
| 3560 | 0 | | 2 | 1 | 0 | 0 | 1 | 1 | 1 | 6 | | 0.01 | | Bacteroidetes |
| 7248 | 2 | | 0 | 2 | 0 | 1 | 0 | 0 | 1 | 6 | | 0.01 | | Bacteria |
| 16944 | 1 | | 1 | 2 | 0 | 0 | 1 | 1 | 0 | 6 | | 0.01 | | Firmicutes;Clostridia |
| 22232 | 1 | | 0 | 3 | 0 | 0 | 1 | 0 | 1 | 6 | | 0.01 | | Bacteria |
| 26 | 0 | | 0 | 0 | 1 | 1 | 2 | 2 | 0 | 6 | | 0.01 | | Bacteria |
| 5632 | 0 | | 0 | 0 | 1 | 0 | 3 | 1 | 1 | 6 | | 0.01 | | Bacteroidetes;Bacteroidetes;Bacteroidales;Prevotellaceae |
| 2071 | 0 | | 0 | 0 | 1 | 2 | 0 | 2 | 1 | 6 | | 0.01 | | Bacteroidetes;Bacteroidetes;Bacteroidales;Prevotellaceae;Prevotella |
| 1380 | 0 | | 0 | 0 | 1 | 1 | 1 | 2 | 1 | 6 | | 0.01 | | Bacteria |
| 15998 | 0 | | 0 | 0 | 1 | 1 | 1 | 2 | 1 | 6 | | 0.01 | | Firmicutes;Clostridia;Clostridiales;Ruminococcaceae |
| 12457 | 0 | | 0 | 0 | 1 | 0 | 2 | 1 | 2 | 6 | | 0.01 | | Bacteroidetes;Bacteroidetes;Bacteroidales |
| 12609 | 0 | | 0 | 0 | 1 | 0 | 1 | 2 | 2 | 6 | | 0.01 | | Bacteria |
| 1123 | 1 | | 0 | 0 | 1 | 2 | 1 | 1 | 0 | 6 | | 0.01 | | Firmicutes;Clostridia;Clostridiales;Incertae Sedis XIII |
| 17314 | 1 | | 0 | 0 | 1 | 2 | 1 | 1 | 0 | 6 | | 0.01 | | Firmicutes;Clostridia;Clostridiales;Lachnospiraceae |
| 19032 | 1 | | 0 | 0 | 1 | 1 | 0 | 2 | 1 | 6 | | 0.01 | | Firmicutes;Clostridia;Clostridiales;Ruminococcaceae |
| 9361 | 2 | | 0 | 0 | 1 | 1 | 0 | 1 | 1 | 6 | | 0.01 | | Firmicutes;Clostridia;Clostridiales;Ruminococcaceae |
| 6818 | 3 | | 0 | 0 | 1 | 1 | 1 | 0 | 0 | 6 | | 0.01 | | Firmicutes;Erysipelotrichi;Erysipelotrichales;Erysipelotrichaceae |
| 7444 | 0 | | 1 | 0 | 1 | 0 | 3 | 0 | 1 | 6 | | 0.01 | | Planctomycetes;Planctomycetacia;Planctomycetales;Planctomycetaceae |
| 12124 | 0 | | 1 | 0 | 1 | 1 | 1 | 1 | 1 | 6 | | 0.01 | | Firmicutes;Clostridia;Clostridiales;Ruminococcaceae;Anaerotruncus |
| 6085 | 1 | | 1 | 0 | 1 | 1 | 1 | 1 | 0 | 6 | | 0.01 | | Bacteroidetes;Bacteroidetes;Bacteroidales |
| 7668 | 1 | | 1 | 0 | 1 | 1 | 0 | 1 | 1 | 6 | | 0.01 | | Bacteria |
| 7488 | 0 | | 2 | 0 | 1 | 1 | 0 | 0 | 2 | 6 | | 0.01 | | Bacteria |
| 21643 | 1 | | 2 | 0 | 1 | 1 | 1 | 0 | 0 | 6 | | 0.01 | | Bacteroidetes |
| 816 | 1 | | 2 | 0 | 1 | 1 | 0 | 0 | 1 | 6 | | 0.01 | | Bacteroidetes |
| 22048 | 0 | | 3 | 0 | 1 | 0 | 0 | 1 | 1 | 6 | | 0.01 | | Bacteroidetes |
| 10284 | 1 | | 3 | 0 | 1 | 0 | 1 | 0 | 0 | 6 | | 0.01 | | Firmicutes;Clostridia;Clostridiales;Lachnospiraceae |
| 8462 | 0 | | 0 | 1 | 1 | 1 | 1 | 2 | 0 | 6 | | 0.01 | | Firmicutes;Clostridia;Clostridiales;Ruminococcaceae |
| 5079 | 0 | | 0 | 1 | 1 | 0 | 3 | 0 | 1 | 6 | | 0.01 | | Firmicutes;Clostridia;Clostridiales |
| 21340 | 1 | | 0 | 1 | 1 | 0 | 1 | 1 | 1 | 6 | | 0.01 | | Bacteroidetes |
| 11176 | 2 | | 0 | 1 | 1 | 0 | 1 | 1 | 0 | 6 | | 0.01 | | Bacteroidetes |
| 13711 | 0 | | 1 | 1 | 1 | 0 | 1 | 2 | 0 | 6 | | 0.01 | | Firmicutes;Erysipelotrichi;Erysipelotrichales;Erysipelotrichaceae |
| 22456 | 0 | | 1 | 1 | 1 | 0 | 1 | 2 | 0 | 6 | | 0.01 | | Firmicutes;Clostridia;Clostridiales |
| 15462 | 0 | | 1 | 1 | 1 | 1 | 0 | 1 | 1 | 6 | | 0.01 | | Firmicutes;Clostridia;Clostridiales |
| 11649 | 1 | | 1 | 1 | 1 | 1 | 1 | 0 | 0 | 6 | | 0.01 | | Bacteroidetes |
| 10462 | 1 | | 1 | 1 | 1 | 0 | 1 | 0 | 1 | 6 | | 0.01 | | Firmicutes;Clostridia;Clostridiales;Lachnospiraceae |
| 3317 | 2 | | 1 | 1 | 1 | 0 | 0 | 1 | 0 | 6 | | 0.01 | | Firmicutes;Clostridia;Clostridiales;Ruminococcaceae |
| 16416 | 2 | | 1 | 1 | 1 | 0 | 0 | 1 | 0 | 6 | | 0.01 | | Bacteroidetes;Bacteroidetes;Bacteroidales |
| 22996 | 1 | | 2 | 1 | 1 | 0 | 0 | 1 | 0 | 6 | | 0.01 | | Firmicutes;Clostridia;Clostridiales |
| 4485 | 2 | | 2 | 1 | 1 | 0 | 0 | 0 | 0 | 6 | | 0.01 | | Bacteroidetes;Bacteroidetes;Bacteroidales;Prevotellaceae |
| 22258 | 1 | | 3 | 1 | 1 | 0 | 0 | 0 | 0 | 6 | | 0.01 | | Bacteroidetes;Bacteroidetes;Bacteroidales |
| 23047 | 1 | | 3 | 1 | 1 | 0 | 0 | 0 | 0 | 6 | | 0.01 | | Bacteroidetes;Bacteroidetes;Bacteroidales;Prevotellaceae |
| 10835 | 0 | | 0 | 2 | 1 | 0 | 1 | 2 | 0 | 6 | | 0.01 | | Firmicutes;Clostridia;Clostridiales |
| 10933 | 0 | | 0 | 2 | 1 | 0 | 0 | 2 | 1 | 6 | | 0.01 | | Firmicutes;Clostridia;Clostridiales;Ruminococcaceae |
| 5285 | 0 | | 0 | 2 | 1 | 0 | 1 | 0 | 2 | 6 | | 0.01 | | Bacteroidetes;Bacteroidetes;Bacteroidales |
| 10406 | 2 | | 0 | 2 | 1 | 0 | 0 | 1 | 0 | 6 | | 0.01 | | Firmicutes;Clostridia;Clostridiales;Ruminococcaceae |
| 555 | 2 | | 0 | 2 | 1 | 0 | 0 | 0 | 1 | 6 | | 0.01 | | Firmicutes;Clostridia;Clostridiales |
| 18664 | 1 | | 1 | 2 | 1 | 0 | 0 | 0 | 1 | 6 | | 0.01 | | Bacteroidetes;Bacteroidetes;Bacteroidales |
| 17137 | 1 | | 2 | 2 | 1 | 0 | 0 | 0 | 0 | 6 | | 0.01 | | Bacteroidetes;Bacteroidetes;Bacteroidales;Prevotellaceae |
| 5256 | 0 | | 1 | 3 | 1 | 0 | 0 | 0 | 1 | 6 | | 0.01 | | Bacteria |
| 14939 | 1 | | 0 | 0 | 2 | 1 | 1 | 1 | 0 | 6 | | 0.01 | | Firmicutes;Clostridia;Clostridiales |
| 5009 | 1 | | 1 | 0 | 2 | 2 | 0 | 0 | 0 | 6 | | 0.01 | | Firmicutes;Clostridia;Clostridiales |
| 18082 | 1 | | 2 | 0 | 2 | 0 | 0 | 0 | 1 | 6 | | 0.01 | | Bacteria |
| 3529 | 0 | | 0 | 1 | 2 | 1 | 0 | 1 | 1 | 6 | | 0.01 | | Bacteroidetes;Bacteroidetes;Bacteroidales |
| 13576 | 1 | | 0 | 1 | 2 | 1 | 0 | 1 | 0 | 6 | | 0.01 | | Firmicutes;Clostridia;Clostridiales;Lachnospiraceae |
| 10750 | 0 | | 1 | 1 | 2 | 1 | 1 | 0 | 0 | 6 | | 0.01 | | Chloroflexi;Anaerolineae |
| 8443 | 0 | | 1 | 1 | 2 | 0 | 1 | 1 | 0 | 6 | | 0.01 | | Bacteroidetes |
| 21735 | 1 | | 1 | 1 | 2 | 0 | 1 | 0 | 0 | 6 | | 0.01 | | Firmicutes;Clostridia;Clostridiales;Ruminococcaceae |
| 17839 | 1 | | 1 | 1 | 2 | 0 | 0 | 1 | 0 | 6 | | 0.01 | | Bacteria |
| 18468 | 0 | | 2 | 1 | 2 | 0 | 0 | 1 | 0 | 6 | | 0.01 | | Firmicutes;Clostridia;Clostridiales;Lachnospiraceae |
| 23660 | 0 | | 2 | 1 | 2 | 0 | 0 | 1 | 0 | 6 | | 0.01 | | Firmicutes;Clostridia;Clostridiales |
| 1886 | 0 | | 1 | 2 | 2 | 0 | 0 | 0 | 1 | 6 | | 0.01 | | Bacteria |
| 406 | 1 | | 1 | 2 | 2 | 0 | 0 | 0 | 0 | 6 | | 0.01 | | Firmicutes;Clostridia;Clostridiales;Ruminococcaceae;Ruminococcus |
| 3405 | 1 | | 1 | 2 | 2 | 0 | 0 | 0 | 0 | 6 | | 0.01 | | Bacteroidetes |
| 18840 | 1 | | 1 | 2 | 2 | 0 | 0 | 0 | 0 | 6 | | 0.01 | | Bacteria |
| 1090 | 0 | | 1 | 1 | 3 | 0 | 1 | 0 | 0 | 6 | | 0.01 | | Firmicutes;Clostridia;Clostridiales;Ruminococcaceae;Sporobacter |
| 745 | 1 | | 1 | 1 | 3 | 0 | 0 | 0 | 0 | 6 | | 0.01 | | Bacteroidetes;Bacteroidetes;Bacteroidales;Prevotellaceae |
| 6705 | 0 | | 0 | 0 | 0 | 2 | 1 | 1 | 1 | 5 | | 0.01 | | Firmicutes;Clostridia;Clostridiales;Ruminococcaceae |
| 9413 | 0 | | 0 | 0 | 0 | 2 | 1 | 1 | 1 | 5 | | 0.01 | | Firmicutes;Clostridia |
| 16258 | 0 | | 0 | 0 | 0 | 2 | 1 | 1 | 1 | 5 | | 0.01 | | Bacteroidetes |
| 22280 | 0 | | 0 | 0 | 0 | 2 | 1 | 1 | 1 | 5 | | 0.01 | | Bacteria |
| 374 | 0 | | 0 | 0 | 0 | 1 | 2 | 1 | 1 | 5 | | 0.01 | | Bacteria |
| 10571 | 0 | | 0 | 0 | 0 | 1 | 2 | 1 | 1 | 5 | | 0.01 | | Bacteroidetes;Bacteroidetes;Bacteroidales |
| 15454 | 0 | | 0 | 0 | 0 | 1 | 2 | 1 | 1 | 5 | | 0.01 | | Bacteroidetes;Bacteroidetes;Bacteroidales;Prevotellaceae |
| 17635 | 0 | | 0 | 0 | 0 | 1 | 2 | 1 | 1 | 5 | | 0.01 | | Bacteroidetes;Bacteroidetes;Bacteroidales |
| 20911 | 0 | | 0 | 0 | 0 | 1 | 2 | 1 | 1 | 5 | | 0.01 | | Bacteroidetes;Bacteroidetes;Bacteroidales;Prevotellaceae |
| 181 | 0 | | 0 | 0 | 0 | 1 | 1 | 2 | 1 | 5 | | 0.01 | | Bacteroidetes;Bacteroidetes;Bacteroidales;Prevotellaceae;Prevotella |
| 12486 | 0 | | 0 | 0 | 0 | 1 | 1 | 1 | 2 | 5 | | 0.01 | | Bacteroidetes |
| 17045 | 0 | | 0 | 0 | 0 | 1 | 1 | 1 | 2 | 5 | | 0.01 | | Bacteroidetes;Bacteroidetes;Bacteroidales;Prevotellaceae |
| 3400 | 1 | | 0 | 0 | 0 | 1 | 2 | 1 | 0 | 5 | | 0.01 | | Bacteroidetes;Bacteroidetes;Bacteroidales;Prevotellaceae |
| 4020 | 1 | | 0 | 0 | 0 | 1 | 2 | 0 | 1 | 5 | | 0.01 | | Firmicutes;Clostridia;Clostridiales;Lachnospiraceae |
| 20359 | 1 | | 0 | 0 | 0 | 0 | 2 | 1 | 1 | 5 | | 0.01 | | Bacteroidetes |
| 10639 | 1 | | 0 | 0 | 0 | 0 | 1 | 2 | 1 | 5 | | 0.01 | | Firmicutes |
| 22416 | 1 | | 0 | 0 | 0 | 1 | 0 | 1 | 2 | 5 | | 0.01 | | Firmicutes;Clostridia;Clostridiales |
| 16562 | 0 | | 1 | 0 | 0 | 2 | 1 | 0 | 1 | 5 | | 0.01 | | Bacteroidetes |
| 21934 | 0 | | 1 | 0 | 0 | 1 | 2 | 0 | 1 | 5 | | 0.01 | | Bacteroidetes |
| 2469 | 0 | | 1 | 0 | 0 | 0 | 2 | 1 | 1 | 5 | | 0.01 | | Firmicutes;Clostridia;Clostridiales;Veillonellaceae |
| 3725 | 0 | | 1 | 0 | 0 | 1 | 0 | 2 | 1 | 5 | | 0.01 | | Bacteroidetes;Bacteroidetes;Bacteroidales;Prevotellaceae;Prevotella |
| 8627 | 0 | | 1 | 0 | 0 | 1 | 0 | 2 | 1 | 5 | | 0.01 | | Bacteroidetes |
| 3413 | 0 | | 1 | 0 | 0 | 1 | 1 | 0 | 2 | 5 | | 0.01 | | Firmicutes;Clostridia;Clostridiales;Ruminococcaceae;Ruminococcus |
| 8850 | 0 | | 1 | 0 | 0 | 0 | 1 | 1 | 2 | 5 | | 0.01 | | Bacteroidetes;Bacteroidetes;Bacteroidales |
| 15474 | 1 | | 1 | 0 | 0 | 1 | 1 | 1 | 0 | 5 | | 0.01 | | Firmicutes;Clostridia;Clostridiales;Lachnospiraceae;Butyrivibrio |
| 14658 | 1 | | 1 | 0 | 0 | 0 | 0 | 1 | 2 | 5 | | 0.01 | | Firmicutes;Clostridia;Clostridiales |
| 4723 | 2 | | 1 | 0 | 0 | 1 | 0 | 0 | 1 | 5 | | 0.01 | | Firmicutes;Clostridia;Clostridiales;Ruminococcaceae |
| 21940 | 0 | | 2 | 0 | 0 | 1 | 0 | 1 | 1 | 5 | | 0.01 | | Firmicutes;Clostridia;Clostridiales;Ruminococcaceae |
| 9637 | 0 | | 0 | 1 | 0 | 2 | 0 | 1 | 1 | 5 | | 0.01 | | Bacteroidetes |
| 19882 | 0 | | 0 | 1 | 0 | 2 | 0 | 1 | 1 | 5 | | 0.01 | | Firmicutes;Clostridia |
| 9555 | 0 | | 0 | 1 | 0 | 1 | 1 | 1 | 1 | 5 | | 0.01 | | Bacteria |
| 21246 | 0 | | 0 | 1 | 0 | 0 | 1 | 2 | 1 | 5 | | 0.01 | | Firmicutes;Clostridia;Clostridiales |
| 6646 | 0 | | 0 | 1 | 0 | 1 | 1 | 0 | 2 | 5 | | 0.01 | | Firmicutes;Clostridia;Clostridiales;Ruminococcaceae |
| 2682 | 2 | | 0 | 1 | 0 | 1 | 1 | 0 | 0 | 5 | | 0.01 | | Firmicutes;Clostridia;Clostridiales;Ruminococcaceae |
| 2864 | 0 | | 1 | 1 | 0 | 1 | 1 | 0 | 1 | 5 | | 0.01 | | Firmicutes;Clostridia;Clostridiales;Ruminococcaceae |
| 6811 | 0 | | 1 | 1 | 0 | 1 | 1 | 0 | 1 | 5 | | 0.01 | | Bacteria |
| 5915 | 0 | | 1 | 1 | 0 | 0 | 2 | 0 | 1 | 5 | | 0.01 | | Firmicutes;Clostridia;Clostridiales |
| 4122 | 0 | | 1 | 1 | 0 | 1 | 0 | 1 | 1 | 5 | | 0.01 | | Firmicutes;Clostridia;Clostridiales |
| 2632 | 0 | | 1 | 1 | 0 | 0 | 1 | 1 | 1 | 5 | | 0.01 | | Bacteroidetes;Bacteroidetes;Bacteroidales |
| 5063 | 0 | | 1 | 1 | 0 | 0 | 1 | 1 | 1 | 5 | | 0.01 | | Firmicutes;Clostridia;Clostridiales |
| 2192 | 1 | | 1 | 1 | 0 | 1 | 1 | 0 | 0 | 5 | | 0.01 | | Firmicutes;Clostridia;Clostridiales;Lachnospiraceae |
| 13166 | 1 | | 1 | 1 | 0 | 0 | 2 | 0 | 0 | 5 | | 0.01 | | Firmicutes;Clostridia;Clostridiales;Ruminococcaceae;Sporobacter |
| 3542 | 2 | | 1 | 1 | 0 | 0 | 0 | 1 | 0 | 5 | | 0.01 | | Bacteroidetes;Bacteroidetes;Bacteroidales |
| 11052 | 0 | | 2 | 1 | 0 | 0 | 0 | 1 | 1 | 5 | | 0.01 | | Firmicutes;Clostridia;Clostridiales;Ruminococcaceae |
| 16419 | 1 | | 2 | 1 | 0 | 0 | 0 | 0 | 1 | 5 | | 0.01 | | Firmicutes;Clostridia;Clostridiales |
| 19547 | 1 | | 2 | 1 | 0 | 0 | 0 | 0 | 1 | 5 | | 0.01 | | Bacteroidetes |
| 4565 | 0 | | 0 | 2 | 0 | 1 | 1 | 0 | 1 | 5 | | 0.01 | | Firmicutes;Clostridia;Clostridiales;Lachnospiraceae |
| 5628 | 1 | | 0 | 2 | 0 | 0 | 0 | 1 | 1 | 5 | | 0.01 | | Bacteria |
| 15049 | 1 | | 1 | 2 | 0 | 0 | 0 | 0 | 1 | 5 | | 0.01 | | Firmicutes;Clostridia;Clostridiales;Incertae Sedis XIII;Anaerovorax |
| 22787 | 0 | | 0 | 0 | 1 | 2 | 1 | 1 | 0 | 5 | | 0.01 | | Bacteroidetes;Bacteroidetes;Bacteroidales |
| 11923 | 0 | | 0 | 0 | 1 | 1 | 2 | 0 | 1 | 5 | | 0.01 | | Firmicutes;Clostridia;Clostridiales;Lachnospiraceae |
| 19945 | 0 | | 0 | 0 | 1 | 1 | 1 | 1 | 1 | 5 | | 0.01 | | Spirochaetes;Spirochaetes;Spirochaetales;Spirochaetaceae;Treponema |
| 20293 | 0 | | 0 | 0 | 1 | 1 | 1 | 0 | 2 | 5 | | 0.01 | | Bacteria |
| 16025 | 0 | | 0 | 0 | 1 | 0 | 1 | 1 | 2 | 5 | | 0.01 | | Firmicutes;Clostridia;Clostridiales;Ruminococcaceae |
| 8815 | 1 | | 0 | 0 | 1 | 2 | 1 | 0 | 0 | 5 | | 0.01 | | Firmicutes;Clostridia;Clostridiales;Lachnospiraceae;Lachnospiraceae Incertae Sedis |
| 18049 | 1 | | 0 | 0 | 1 | 1 | 1 | 1 | 0 | 5 | | 0.01 | | Firmicutes;Clostridia;Clostridiales;Lachnospiraceae;Lachnospiraceae Incertae Sedis |
| 15720 | 1 | | 0 | 0 | 1 | 0 | 1 | 2 | 0 | 5 | | 0.01 | | Firmicutes;Clostridia;Clostridiales;Lachnospiraceae |
| 14062 | 1 | | 0 | 0 | 1 | 2 | 0 | 0 | 1 | 5 | | 0.01 | | Firmicutes;Clostridia;Clostridiales;Ruminococcaceae |
| 4365 | 1 | | 0 | 0 | 1 | 0 | 0 | 2 | 1 | 5 | | 0.01 | | Bacteria |
| 19133 | 0 | | 1 | 0 | 1 | 1 | 1 | 0 | 1 | 5 | | 0.01 | | Bacteroidetes |
| 2442 | 0 | | 1 | 0 | 1 | 1 | 0 | 1 | 1 | 5 | | 0.01 | | Bacteroidetes;Bacteroidetes;Bacteroidales;Prevotellaceae |
| 19335 | 0 | | 1 | 0 | 1 | 1 | 0 | 1 | 1 | 5 | | 0.01 | | Firmicutes;Clostridia;Clostridiales;Lachnospiraceae |
| 12243 | 1 | | 1 | 0 | 1 | 2 | 0 | 0 | 0 | 5 | | 0.01 | | Bacteroidetes;Bacteroidetes;Bacteroidales |
| 16188 | 1 | | 1 | 0 | 1 | 1 | 1 | 0 | 0 | 5 | | 0.01 | | Firmicutes;Clostridia;Clostridiales |
| 7611 | 0 | | 2 | 0 | 1 | 1 | 0 | 1 | 0 | 5 | | 0.01 | | Bacteroidetes;Bacteroidetes;Bacteroidales;Prevotellaceae;Prevotella |
| 14458 | 0 | | 2 | 0 | 1 | 1 | 0 | 0 | 1 | 5 | | 0.01 | | Firmicutes;Clostridia;Clostridiales |
| 15678 | 0 | | 0 | 1 | 1 | 1 | 1 | 0 | 1 | 5 | | 0.01 | | Bacteria |
| 20012 | 0 | | 0 | 1 | 1 | 1 | 1 | 0 | 1 | 5 | | 0.01 | | Bacteroidetes;Bacteroidetes;Bacteroidales;Prevotellaceae;Prevotella |
| 2378 | 0 | | 0 | 1 | 1 | 0 | 1 | 0 | 2 | 5 | | 0.01 | | Firmicutes;Clostridia;Clostridiales;Ruminococcaceae |
| 5404 | 0 | | 0 | 1 | 1 | 0 | 0 | 1 | 2 | 5 | | 0.01 | | Firmicutes |
| 1977 | 1 | | 0 | 1 | 1 | 1 | 1 | 0 | 0 | 5 | | 0.01 | | Firmicutes;Clostridia;Clostridiales |
| 7980 | 1 | | 0 | 1 | 1 | 1 | 1 | 0 | 0 | 5 | | 0.01 | | Bacteroidetes;Bacteroidetes;Bacteroidales |
| 5069 | 0 | | 1 | 1 | 1 | 0 | 1 | 1 | 0 | 5 | | 0.01 | | Bacteroidetes;Bacteroidetes;Bacteroidales |
| 11515 | 0 | | 1 | 1 | 1 | 0 | 0 | 2 | 0 | 5 | | 0.01 | | Firmicutes;Clostridia;Clostridiales;Lachnospiraceae |
| 830 | 0 | | 1 | 1 | 1 | 0 | 1 | 0 | 1 | 5 | | 0.01 | | Firmicutes;Clostridia;Clostridiales;Ruminococcaceae |
| 3587 | 0 | | 1 | 1 | 1 | 0 | 0 | 1 | 1 | 5 | | 0.01 | | Firmicutes |
| 12037 | 1 | | 1 | 1 | 1 | 0 | 1 | 0 | 0 | 5 | | 0.01 | | Firmicutes;Clostridia;Clostridiales |
| 13937 | 1 | | 1 | 1 | 1 | 0 | 0 | 0 | 1 | 5 | | 0.01 | | Bacteria |
| 834 | 2 | | 1 | 1 | 1 | 0 | 0 | 0 | 0 | 5 | | 0.01 | | Bacteroidetes |
| 12276 | 2 | | 1 | 1 | 1 | 0 | 0 | 0 | 0 | 5 | | 0.01 | | Firmicutes;Clostridia;Clostridiales |
| 6957 | 0 | | 2 | 1 | 1 | 0 | 1 | 0 | 0 | 5 | | 0.01 | | Chloroflexi;Anaerolineae |
| 20263 | 0 | | 2 | 1 | 1 | 0 | 0 | 0 | 1 | 5 | | 0.01 | | Firmicutes |
| 8080 | 1 | | 2 | 1 | 1 | 0 | 0 | 0 | 0 | 5 | | 0.01 | | Bacteroidetes;Bacteroidetes;Bacteroidales;Prevotellaceae |
| 19666 | 1 | | 2 | 1 | 1 | 0 | 0 | 0 | 0 | 5 | | 0.01 | | Firmicutes;Clostridia;Clostridiales;Lachnospiraceae |
| 8524 | 0 | | 0 | 2 | 1 | 1 | 0 | 0 | 1 | 5 | | 0.01 | | Bacteria |
| 3899 | 1 | | 0 | 2 | 1 | 0 | 1 | 0 | 0 | 5 | | 0.01 | | Firmicutes;Clostridia;Clostridiales;Ruminococcaceae |
| 4285 | 0 | | 1 | 2 | 1 | 0 | 1 | 0 | 0 | 5 | | 0.01 | | Firmicutes;Clostridia;Clostridiales;Ruminococcaceae |
| 11400 | 1 | | 1 | 2 | 1 | 0 | 0 | 0 | 0 | 5 | | 0.01 | | Bacteroidetes;Bacteroidetes;Bacteroidales;Prevotellaceae |
| 16031 | 1 | | 1 | 2 | 1 | 0 | 0 | 0 | 0 | 5 | | 0.01 | | Firmicutes;Clostridia;Clostridiales;Ruminococcaceae |
| 21462 | 0 | | 0 | 0 | 2 | 1 | 1 | 1 | 0 | 5 | | 0.01 | | Firmicutes;Clostridia;Clostridiales;Lachnospiraceae |
| 7480 | 0 | | 0 | 0 | 2 | 1 | 1 | 0 | 1 | 5 | | 0.01 | | Firmicutes;Clostridia;Clostridiales;Lachnospiraceae;Lachnospiraceae Incertae Sedis |
| 21440 | 0 | | 0 | 0 | 2 | 0 | 1 | 1 | 1 | 5 | | 0.01 | | Firmicutes;Clostridia |
| 23536 | 1 | | 0 | 0 | 2 | 1 | 0 | 0 | 1 | 5 | | 0.01 | | Firmicutes;Clostridia;Clostridiales |
| 7295 | 1 | | 0 | 0 | 2 | 0 | 1 | 0 | 1 | 5 | | 0.01 | | Firmicutes;Clostridia;Clostridiales;Ruminococcaceae;Ruminococcus |
| 22070 | 1 | | 1 | 0 | 2 | 0 | 1 | 0 | 0 | 5 | | 0.01 | | Bacteroidetes |
| 9075 | 1 | | 0 | 1 | 2 | 0 | 0 | 1 | 0 | 5 | | 0.01 | | Firmicutes;Clostridia;Clostridiales;Lachnospiraceae;Lachnospiraceae Incertae Sedis |
| 666 | 1 | | 1 | 1 | 2 | 0 | 0 | 0 | 0 | 5 | | 0.01 | | Bacteroidetes;Bacteroidetes;Bacteroidales |
| 5178 | 1 | | 1 | 1 | 2 | 0 | 0 | 0 | 0 | 5 | | 0.01 | | Bacteroidetes;Bacteroidetes;Bacteroidales;Prevotellaceae |
| 10447 | 1 | | 1 | 1 | 2 | 0 | 0 | 0 | 0 | 5 | | 0.01 | | Bacteroidetes |
| 13123 | 1 | | 1 | 1 | 2 | 0 | 0 | 0 | 0 | 5 | | 0.01 | | Firmicutes;Clostridia;Clostridiales;Ruminococcaceae |
| 843 | 0 | | 0 | 0 | 0 | 1 | 1 | 1 | 1 | 4 | | 0.01 | | Bacteroidetes;Bacteroidetes;Bacteroidales;Prevotellaceae;Prevotella |
| 12237 | 0 | | 0 | 0 | 0 | 1 | 1 | 1 | 1 | 4 | | 0.01 | | Firmicutes;Clostridia;Clostridiales;Ruminococcaceae |
| 12655 | 0 | | 0 | 0 | 0 | 1 | 1 | 1 | 1 | 4 | | 0.01 | | Bacteroidetes;Bacteroidetes;Bacteroidales |
| 16116 | 0 | | 0 | 0 | 0 | 1 | 1 | 1 | 1 | 4 | | 0.01 | | Firmicutes;Clostridia;Clostridiales;Ruminococcaceae;Ruminococcus |
| 16932 | 0 | | 0 | 0 | 0 | 1 | 1 | 1 | 1 | 4 | | 0.01 | | Bacteroidetes |
| 23696 | 0 | | 0 | 0 | 0 | 1 | 1 | 1 | 1 | 4 | | 0.01 | | Bacteroidetes;Bacteroidetes;Bacteroidales |
| 14753 | 1 | | 0 | 0 | 0 | 1 | 1 | 1 | 0 | 4 | | 0.01 | | Firmicutes;Clostridia;Clostridiales;Ruminococcaceae |
| 1716 | 1 | | 0 | 0 | 0 | 1 | 1 | 0 | 1 | 4 | | 0.01 | | Firmicutes;Clostridia;Clostridiales;Ruminococcaceae |
| 9216 | 1 | | 0 | 0 | 0 | 1 | 0 | 1 | 1 | 4 | | 0.01 | | Bacteroidetes;Bacteroidetes;Bacteroidales |
| 19493 | 1 | | 0 | 0 | 0 | 1 | 0 | 1 | 1 | 4 | | 0.01 | | Bacteroidetes |
| 17955 | 0 | | 1 | 0 | 0 | 1 | 1 | 1 | 0 | 4 | | 0.01 | | Bacteria |
| 2272 | 0 | | 1 | 0 | 0 | 1 | 1 | 0 | 1 | 4 | | 0.01 | | Bacteroidetes |
| 6207 | 0 | | 1 | 0 | 0 | 0 | 1 | 1 | 1 | 4 | | 0.01 | | Bacteroidetes;Bacteroidetes;Bacteroidales |
| 4687 | 1 | | 1 | 0 | 0 | 1 | 1 | 0 | 0 | 4 | | 0.01 | | Bacteria |
| 7691 | 1 | | 1 | 0 | 0 | 1 | 0 | 1 | 0 | 4 | | 0.01 | | Firmicutes;Clostridia;Clostridiales;Ruminococcaceae |
| 17337 | 1 | | 1 | 0 | 0 | 0 | 1 | 0 | 1 | 4 | | 0.01 | | Bacteroidetes |
| 2487 | 1 | | 1 | 0 | 0 | 0 | 0 | 1 | 1 | 4 | | 0.01 | | Firmicutes;Clostridia;Clostridiales;Ruminococcaceae;Sporobacter |
| 23612 | 0 | | 0 | 1 | 0 | 1 | 1 | 1 | 0 | 4 | | 0.01 | | Bacteria |
| 11716 | 0 | | 0 | 1 | 0 | 1 | 0 | 1 | 1 | 4 | | 0.01 | | Firmicutes;Clostridia;Clostridiales |
| 1395 | 0 | | 0 | 1 | 0 | 0 | 1 | 1 | 1 | 4 | | 0.01 | | Firmicutes;Clostridia;Clostridiales |
| 6082 | 1 | | 0 | 1 | 0 | 0 | 1 | 0 | 1 | 4 | | 0.01 | | Chloroflexi;Anaerolineae |
| 8725 | 1 | | 0 | 1 | 0 | 0 | 0 | 1 | 1 | 4 | | 0.01 | | Firmicutes;Clostridia;Clostridiales;Lachnospiraceae |
| 14603 | 0 | | 1 | 1 | 0 | 1 | 0 | 0 | 1 | 4 | | 0.01 | | Bacteroidetes;Bacteroidetes;Bacteroidales;Prevotellaceae |
| 14597 | 0 | | 1 | 1 | 0 | 0 | 1 | 0 | 1 | 4 | | 0.01 | | Firmicutes;Clostridia;Clostridiales;Ruminococcaceae |
| 4090 | 0 | | 1 | 1 | 0 | 0 | 0 | 1 | 1 | 4 | | 0.01 | | Bacteria |
| 23246 | 1 | | 1 | 1 | 0 | 0 | 0 | 0 | 1 | 4 | | 0.01 | | Firmicutes;Clostridia;Clostridiales;Ruminococcaceae |
| 7578 | 0 | | 0 | 0 | 1 | 1 | 1 | 1 | 0 | 4 | | 0.01 | | Bacteroidetes |
| 10506 | 0 | | 0 | 0 | 1 | 1 | 1 | 1 | 0 | 4 | | 0.01 | | Bacteroidetes;Bacteroidetes;Bacteroidales;Prevotellaceae |
| 14555 | 0 | | 0 | 0 | 1 | 1 | 1 | 1 | 0 | 4 | | 0.01 | | Firmicutes;Clostridia;Clostridiales;Ruminococcaceae |
| 14931 | 0 | | 0 | 0 | 1 | 1 | 1 | 0 | 1 | 4 | | 0.01 | | Bacteroidetes |
| 3240 | 0 | | 0 | 0 | 1 | 0 | 1 | 1 | 1 | 4 | | 0.01 | | Bacteroidetes;Bacteroidetes;Bacteroidales |
| 6028 | 0 | | 0 | 0 | 1 | 0 | 1 | 1 | 1 | 4 | | 0.01 | | Bacteroidetes;Bacteroidetes;Bacteroidales;Prevotellaceae;Hallella |
| 18134 | 0 | | 0 | 0 | 1 | 0 | 1 | 1 | 1 | 4 | | 0.01 | | Bacteroidetes;Bacteroidetes;Bacteroidales |
| 10077 | 1 | | 0 | 0 | 1 | 1 | 1 | 0 | 0 | 4 | | 0.01 | | Bacteroidetes;Bacteroidetes;Bacteroidales;Prevotellaceae;Prevotella |
| 695 | 1 | | 0 | 0 | 1 | 1 | 0 | 1 | 0 | 4 | | 0.01 | | Firmicutes;Clostridia;Clostridiales |
| 15186 | 1 | | 0 | 0 | 1 | 0 | 1 | 0 | 1 | 4 | | 0.01 | | Chloroflexi;Anaerolineae |
| 8506 | 1 | | 0 | 0 | 1 | 0 | 0 | 1 | 1 | 4 | | 0.01 | | Bacteroidetes;Bacteroidetes;Bacteroidales;Prevotellaceae |
| 20010 | 1 | | 0 | 0 | 1 | 0 | 0 | 1 | 1 | 4 | | 0.01 | | TM7;TM7_genera_incertae_sedis |
| 2339 | 0 | | 1 | 0 | 1 | 1 | 1 | 0 | 0 | 4 | | 0.01 | | Bacteroidetes;Bacteroidetes;Bacteroidales |
| 23145 | 0 | | 1 | 0 | 1 | 1 | 1 | 0 | 0 | 4 | | 0.01 | | Bacteria |
| 15604 | 0 | | 1 | 0 | 1 | 0 | 1 | 1 | 0 | 4 | | 0.01 | | Firmicutes;Clostridia;Clostridiales |
| 656 | 1 | | 1 | 0 | 1 | 0 | 1 | 0 | 0 | 4 | | 0.01 | | Bacteroidetes;Bacteroidetes;Bacteroidales |
| 15828 | 1 | | 1 | 0 | 1 | 0 | 0 | 1 | 0 | 4 | | 0.01 | | Bacteroidetes;Bacteroidetes;Bacteroidales;Prevotellaceae |
| 7523 | 1 | | 1 | 0 | 1 | 0 | 0 | 0 | 1 | 4 | | 0.01 | | Firmicutes;Clostridia;Clostridiales;Ruminococcaceae |
| 10109 | 1 | | 1 | 0 | 1 | 0 | 0 | 0 | 1 | 4 | | 0.01 | | Firmicutes;Clostridia;Clostridiales;Lachnospiraceae |
| 10260 | 1 | | 1 | 0 | 1 | 0 | 0 | 0 | 1 | 4 | | 0.01 | | Bacteroidetes;Bacteroidetes;Bacteroidales |
| 15492 | 0 | | 0 | 1 | 1 | 1 | 0 | 1 | 0 | 4 | | 0.01 | | Firmicutes;Clostridia;Clostridiales |
| 7806 | 0 | | 0 | 1 | 1 | 0 | 1 | 1 | 0 | 4 | | 0.01 | | Firmicutes;Clostridia;Clostridiales;Ruminococcaceae |
| 3488 | 0 | | 0 | 1 | 1 | 0 | 1 | 0 | 1 | 4 | | 0.01 | | Firmicutes;Clostridia;Clostridiales;Ruminococcaceae;Sporobacter |
| 15643 | 1 | | 0 | 1 | 1 | 0 | 1 | 0 | 0 | 4 | | 0.01 | | Bacteria |
| 10137 | 1 | | 0 | 1 | 1 | 0 | 0 | 1 | 0 | 4 | | 0.01 | | Bacteroidetes |
| 17871 | 1 | | 0 | 1 | 1 | 0 | 0 | 1 | 0 | 4 | | 0.01 | | Firmicutes;Clostridia;Clostridiales |
| 3512 | 0 | | 1 | 1 | 1 | 1 | 0 | 0 | 0 | 4 | | 0.01 | | Firmicutes;Clostridia;Clostridiales;Lachnospiraceae |
| 12671 | 0 | | 1 | 1 | 1 | 1 | 0 | 0 | 0 | 4 | | 0.01 | | Bacteroidetes;Bacteroidetes;Bacteroidales;Prevotellaceae |
| 521 | 0 | | 1 | 1 | 1 | 0 | 1 | 0 | 0 | 4 | | 0.01 | | Bacteroidetes;Bacteroidetes;Bacteroidales |
| 12788 | 0 | | 1 | 1 | 1 | 0 | 1 | 0 | 0 | 4 | | 0.01 | | Bacteroidetes;Bacteroidetes;Bacteroidales |
| 20879 | 0 | | 1 | 1 | 1 | 0 | 1 | 0 | 0 | 4 | | 0.01 | | Firmicutes;Clostridia;Clostridiales;Ruminococcaceae |
| 21575 | 0 | | 1 | 1 | 1 | 0 | 0 | 0 | 1 | 4 | | 0.01 | | Bacteroidetes;Bacteroidetes;Bacteroidales |
| 1476 | 1 | | 1 | 1 | 1 | 0 | 0 | 0 | 0 | 4 | | 0.01 | | Bacteroidetes;Bacteroidetes;Bacteroidales |
| 8272 | 1 | | 1 | 1 | 1 | 0 | 0 | 0 | 0 | 4 | | 0.01 | | Bacteroidetes;Bacteroidetes;Bacteroidales;Prevotellaceae;Prevotella |
| 8891 | 1 | | 1 | 1 | 1 | 0 | 0 | 0 | 0 | 4 | | 0.01 | | Bacteria |
| 10948 | 1 | | 1 | 1 | 1 | 0 | 0 | 0 | 0 | 4 | | 0.01 | | Bacteroidetes;Bacteroidetes;Bacteroidales;Prevotellaceae |
| 12872 | 1 | | 1 | 1 | 1 | 0 | 0 | 0 | 0 | 4 | | 0.01 | | Firmicutes |
| 13280 | 1 | | 1 | 1 | 1 | 0 | 0 | 0 | 0 | 4 | | 0.01 | | Bacteroidetes |
| 17488 | 1 | | 1 | 1 | 1 | 0 | 0 | 0 | 0 | 4 | | 0.01 | | Bacteroidetes |
| **Total seqs.** | 3227 | | 5074 | 4383 | 5999 | 5789 | 8238 | 7026 | 8244 | 47980 |  | |  | |
| Total OTUs | | | |  | 1182 |  |  |  |  |  |  | |  | |
| Total OTUs SR1 only | | | |  | 100 (91.5% OTUs shared) | | | |  |  |  | |  | |
| Total OTUs SR2 only | | | |  | 113 (90.4% OTUs shared) | | | |  |  |  | |  | |
| Total OTUs Genus-assignment | | | | | 177 (15%) | |  |  |  |  |  | |  | |
| Total OTUs Family-assignment | | | | | 561 (47%) | |  |  |  |  |  | |  | |
| Total OTUs Order-assignment | | | | | 839 (71%) | |  |  |  |  |  | |  | |
| Total OTUs Phylum-assignment | | | | | 1046 (88%) | |  |  |  |  |  | |  | |

* Hierarchical taxonomic assignment for each OTU calculated using the RDP naïve Bayesian Classifier [13]. Lineages are displayed only where OTUs could be assigned with an 80% bootstrap confidence estimate. SR1 and SR2 indicate animal number and **a-d** indicate PCR replicates used for OTU filtering (see Materials and Methods). Rows highlighted in yellow indicate OTUs shared with all ruminant and foregut samples (see Text and Fig. 1b).
