# Supplementary material for: Metagenomics of the Svalbard Reindeer Rumen Microbiome Reveals Abundance of Polysaccharide Utilization Loci
Source: PLoS One. 2012 Jun 6;7(6):e38571. doi: 10.1371/journal.pone.0038571 (PMC3368933; doi:10.1371/journal.pone.0038571)
Supplement: Table S2 — Phylogenetic profile of the Svalbard reindeer rumen metagenome sequence dataset, based on sequence composition-based binning of assembled scaffolds using PhyloPythiaS. Values described in non-bold text represent sub-category counts for order/family lineages within the Bacteroidetes and Firmicutes. (DOC) [file pone.0038571.s002.doc]

Table S2. Phylogenetic profile of the Svalbard reindeer rumen metagenome sequence dataset, based on sequence composition-based binning of assembled scaffolds using PhyloPythiaS.

| Taxonomic group | # scaf. | % | bp | % |
| --- | --- | --- | --- | --- |
| **Bacteroidetes** | **603** | **44,21** | **2879182** | **52,96** |
| Bacteroidetes (unclassified) | 10 | 0,73 | 32295 | 0,59 |
| Bacteroidales (unclassified) | 184 | 13,49 | 871854 | 16,04 |
| Bacteroidales (unclassified: SRM-1) | 145 | 10,63 | 887812 | 16,33 |
| Porphyromonadaceae (unclassified) | 1 | 0,07 | 2473 | 0,05 |
| Prevotellaceae (unclassified) | 105 | 7,70 | 376623 | 6,93 |
| Rikenellaceae (unclassified) | 14 | 1,03 | 49626 | 0,91 |
| Bacteroidaceae (unclassified) | 143 | 10,48 | 655923 | 12,06 |
| Sphingobacteria; Sphingobacteriales (unclassified) | 0 | 0,00 | 0 | 0,00 |
| Flavobacteria (unclassified) | 1 | 0,07 | 2576 | 0,05 |
| **Actinobacteria** | **2** | **0,15** | **5703** | **0,10** |
| **Chloroflexi** | **0** | **0,00** | **0** | **0,00** |
| **Firmicutes** | **426** | **31,23** | **1501652** | **27,62** |
| unclassified_Firmicutes | 87 | 6,38 | 316457 | 5,82 |
| Clostridiales | 328 | 24,05 | 1151251 | 21,17 |
| **Other** | **322** | **23,61** | **1012743** | **18,63** |
| **Planctomycetes** | **0** | **0,00** | **0** | **0,00** |
| **Proteobacteria** | **10** | **0,73** | **34362** | **0,63** |
| **Spirochaetes** | **1** | **0,07** | **3327** | **0,06** |
| **TM7** | **0** | **0,00** | **0** | **0,00** |
| **TOTAL** | **1364** |  | **5436969** |  |

Values described in non-bold text represent sub-category counts for order/family lineages within the Bacteroidetes and Firmicutes.
