# Supplementary material for: Metagenomics of the Svalbard Reindeer Rumen Microbiome Reveals Abundance of Polysaccharide Utilization Loci
Source: PLoS One. 2012 Jun 6;7(6):e38571. doi: 10.1371/journal.pone.0038571 (PMC3368933; doi:10.1371/journal.pone.0038571)
Supplement: Table S3 — Input clades, sample specific data and genomes/Whole Genome Shotgun submissions (WGS) used for PhyloPythiaS training models. * temporary ncbid for SRM-1. (DOC) [file pone.0038571.s003.doc]

Table S3. Input clades, sample specific data and genomes/Whole Genome Shotgun submissions (WGS) used for PhyloPythiaS training models

| **Taxonomy**  **ID (ncbi)** | **Clades** | **kb of Sample Specific data** | **genomes WGS** |
| --- | --- | --- | --- |
| **84999** | Actinobacteria;Actinobacteria;Coriobacteridae;Coriobacteriales | 22 | 9 |
| **171549** | Bacteroidetes;Bacteroidetes;Bacteroidales | 0 | 40 |
| **927658*** | Bacteroidetes;Bacteroidetes;Bacteroidales: SRM-1 | 223 | 0 |
| **171551** | Bacteroidetes;Bacteroidetes;Bacteroidales;Porphyromonadaceae | 51 | 7 |
| **171552** | Bacteroidetes;Bacteroidetes;Bacteroidales;Prevotellaceae | 1799 | 3 |
| **171550** | Bacteroidetes;Bacteroidetes;Bacteroidales;Rikenellaceae | 222 | 1 |
| **815** | Bacteroidetes;Bacteroidetes;Bacteroidales;Bacteroidaceae | 845 | 28 |
| **768503** | Bacteroidetes;Cytophagia | 8 | 5 |
| **117743** | Bacteroidetes;Flavobacteria | 30 | 22 |
| **200666** | Bacteroidetes;Sphingobacteria;Sphingobacteriales | 15 | 7 |
| **200795** | Chloroflexi | 0 | 12 |
| **1239** | Firmicutes | 675 | 0 |
| **186802** | Firmicutes;Clostridia;Clostridiales | 1014 | 104 |
| **186803** | Firmicutes;Clostridia;Clostridiales;Lachnospiraceae | 208 | 13 |
| **541000** | Firmicutes;Clostridia;Clostridiales;Ruminococcaceae | 333 | 8 |
| **31979** | Firmicutes;Clostridia;Clostridiales;Clostridiaceae | 285 | 59 |
| **186806** | Firmicutes;Clostridia;Clostridiales;Eubacteriaceae | 20 | 4 |
| **186807** | Firmicutes;Clostridia;Clostridiales;Peptococcaceae | 8 | 6 |
| **31977** | Firmicutes;Negativicutes;Selenomonadales;Veillonellaceae | 283 | 8 |
| **128827** | Firmicutes;Erysipelotrichi;Erysipelotrichales;Erysipelotrichaceae | 55 | 8 |
| **91061** | Firmicutes;Bacilli | 88 | 297 |
| **126** | Planctomycetes;Planctomycetacia;Planctomycetales;Planctomycetaceae | 6 | 5 |
| **1224** | Proteobacteria | 83 | 0 |
| **481** | Proteobacteria;Betaproteobacteria;Neisseriales;Neisseriaceae | 4 | 30 |
| **213121** | Proteobacteria;Deltaproteobacteria;Desulfobacterales;Desulfobulbaceae | 0 | 3 |
| **69541** | Proteobacteria;Deltaproteobacteria;Desulfuromonadales | 9 | 10 |
| **72294** | Proteobacteria;Epsilonproteobacteria;Campylobacterales;Campylobacteraceae | 0 | 23 |
| **28211** | Proteobacteria;Alphaproteobacteria | 38 | 188 |
| **1236** | Proteobacteria;Gammaproteobacteria | 46 | 386 |
| **137** | Spirochaetes;Spirochaetes;Spirochaetales;Spirochaetaceae | 0 | 27 |

*temporary ncbid for SRM-1.
