# Supplementary material for: Metagenomics of the Svalbard Reindeer Rumen Microbiome Reveals Abundance of Polysaccharide Utilization Loci
Source: PLoS One. 2012 Jun 6;7(6):e38571. doi: 10.1371/journal.pone.0038571 (PMC3368933; doi:10.1371/journal.pone.0038571)
Supplement: Table S4 — Glycoside hydrolases and related proteins recovered from the putative partial SRM-1 genome. * indicates best match; † indicates contig is linked to an uncultured Bacteroidales bacterium scaffold, based on PhyloPythiaS analysis; +SP indicates signal peptide detected. Taxonomic assignment was predicted using GC%, high coverage (greater than 6x) and PhyloPythiaS binning. (DOC) [file pone.0038571.s004.doc]

# Table S4. Glycoside hydrolases and related proteins recovered from the putative partial SRM-1 genome.

| **IMG Gene Object ID** | | **Protein/Function** | **Contig** | **Length (bp)** | **G+C %** | **Coverage (x)** | **PhyloPythiaS** | **Homolog *Bacteroidales*** |
| --- | --- | --- | --- | --- | --- | --- | --- | --- |
| **Polysaccharide degradation (Glycosyl Hydrolases)** | | | | | | | | |
| **2081793577** | endoglucanase (GH5) ^+SP^ | | C27962 | 3252 | 66 | 6.3 | SRM-1^†^ | yes* |
| **2081773289** | endoglucanase (GH5) ^+SP^ | | C00447 | 2303 | 62 | 7.6 | SRM-1 | yes* |
| **2081777463** | endoglucanase (GH8) ^+SP^ | | C13783 | 6560 | 64 | 8.2 | SRM-1 | yes* |
| **2081794101/2** | endoglucanase (GH9) ^+SP^ | | C03846 | 6288 | 62 | 8.1 | SRM-1 | yes* |
| **2081822908** | endoglucanase (GH9) | | C05058 | 3204 | 61 | 7.2 | Bacteroidales | yes* |
| **2081772468** | cellobiose phosphorylase (GH94) + CBM_X | | C39344 | 1361 | 65 | 11.5 | Bacteria | yes* |
| **2081816268** | beta-1,4-xylanase (GH10) ^+SP^ | | C04888 | 2065 | 56 | 8.5 | Bacteroidales | yes* |
| **2081793578** | beta mannanase (GH26) ^+SP^ | | C27962 | 3252 | 66 | 6.3 | SRM-1^†^ | yes* |
| **2081786863** | beta-xylosidase (GH43) ^+SP^ | | C06524 | 3652 | 64 | 7.8 | Bacteroidales | yes* |
| **2081797802** | beta-xylosidase (GH43) ^+SP^ | | C01379 | 8438 | 62 | 9.0 | SRM-1 | yes* |
| **2081785072** | beta-xylosidase (GH43) + CBM32 ^+SP^ | | C40359 | 3431 | 62 | 9.6 | Bacteroidales | yes* |
| **2081791037** | beta-xylosidase (GH43) ^+SP^ | | C03614 | 2526 | 62 | 8.7 | Bacteroidales | yes* |
| **2081818727** | beta-xylosidase (GH43) + CBM32 + CE1 ^+SP^ | | C00251 | 7394 | 65 | 6.8 | Bacteria | yes* |
| **2081798881** | beta-xylosidase (GH43) ^+SP^ | | C01425 | 2084 | 65 | 9.2 | Bacteroidales | yes* |
| **2081779239** | alpha-L-arabinofuranosidase (GH51) ^+SP^ | | C02223 | 4525 | 58 | 9.7 | Bacteroidales | yes* |
| **2081778079** | alpha-L-arabinofuranosidase (GH51) | | C02222 | 2412 | 59 | 9.1 | Bacteroidales | yes* |
| **2081800454** | alpha-L-arabinofuranosidase (GH54) ^+SP^ | | C47723 | 3689 | 68 | 7.8 | Bacteroidales | no |
| **2081800455** | alpha-L-arabinofuranosidase (GH54) ^+SP^ | | C47723 | 3689 | 68 | 7.8 | Bacteroidales | no |
| **2081800239** | alpha-L-arabinofuranosidase (GH54) | | C47722 | 2063 | 68 | 9.7 | Bacteroidales | no |
| **2081812193** | arabinogalactan endo-1,4-beta-galactosidase (GH53) | | C02372 | 8023 | 61 | 9.1 | Bacteroidales | yes* |
| **2081821365** | alpha-glucuronidase (GH67) ^+SP^ | | C00321 | 3421 | 64 | 7.6 | Bacteroidales | yes* |
| **2081803189** | endopolygalacturonase (GH28) ^+SP^ | | C48145 | 1782 | 54 | 9.7 | Bacteria | yes* |
| **2081817980** | xylanase/chitin deacetylase (CE4) ^+SP^ | | C42966 | 4905 | 63 | 9.4 | SRM-1 | no |
| **2081781003** | acetyl xylan esterase (CE1) | | C54124 | 1693 | 62 | 10.6 | SRM-1 | yes* |
| **2081818726** | acetyl xylan esterase (CE1) + CBM48 ^+SP^ | | C00251 | 7394 | 65 | 6.8 | Bacteria | yes* |
| **2081781411** | polyphenol oxidoreductase laccase | | C19326 | 10787 | 62 | 7.8 | Bacteroidales | yes* |
| **2081781410** | Carbohydrate esterase / xylanase (CE10) ^+SP^ | | C19326 | 10787 | 62 | 7.8 | Bacteroidales | yes* |
| **2081821927** | alpha-glucosidase (GH97) | | C18209 | 3035 | 64 | 7.2 | Bacteroidales^†^ | yes* |
| **2081774052** | alpha-glucosidase (GH97) | | C09250 | 2047 | 62 | 9.2 | Bacteroidales | yes* |
| **2081813346** | alpha-glucosidase (GH97) | | C02373 | 1496 | 65 | 8.3 | Bacteroidales^†^ |  |
| **2081794100** | beta-galactosidase/beta-glucuronidase (GH2) ^+SP^ | | C03846 | 6288 | 62 | 8.1 | SRM-1 | yes* |
| **2081807071** | beta-galactosidase/beta-glucuronidase (GH2) ^+SP^ | | C63740 | 4871 | 62 | 9.2 | SRM-1 | yes* |
| **2081790219** | beta-glucosidase (GH3) | | C40720 | 4569 | 65 | 8.1 | SRM-1 | yes* |
| **2081771365** | beta-glucosidase (GH3) | | C00397 | 5405 | 62 | 7.2 | Bacteroidales | yes* |
| **2081806499** | beta-glucosidase (GH3) ^+SP^ | | C04450 | 5174 | 65 | 6.5 | SRM-1 | yes* |
| **2081777240** | beta-glucosidase (GH3) | | C13781 | 5264 | 60 | 8.4 | SRM-1 | yes* |
| **2081811987** | beta-glucosidase (GH3) ^+SP^ | | C04698 | 1783 | 63 | 8.5 | SRM-1 | yes* |
| **2081811986** | beta-glucanase (GH16) | | C04698 | 1783 | 63 | 8.5 | SRM-1 | yes* |
| **2081800021** | alpha-amylase (GH13) | | C07269 | 3618 | 61 | 10.2 | SRM-1 | yes* |
| **2081812195** | alpha-amylase (GH13) ^+SP^ | | C02372 | 8023 | 61 | 9.1 | Bacteroidales | yes* |
| **2081812194** | alpha-amylase (GH13) ^+SP^ | | C02372 | 8023 | 61 | 9.1 | Bacteroidales | yes* |
| **2081812192** | alpha-amylase (GH13) ^+SP^ | | C02372 | 8023 | 61 | 9.1 | Bacteroidales | yes* |
| **2081801787** | transglycosylase SLT domain (GH23) ^+SP^ | | C41598 | 5837 | 64 | 10.7 | Bacteroidales | yes* |
| **2081790218** | O-Glycosyl hydrolase (GH30) ^+SP^ | | C40720 | 4569 | 65 | 8.1 | SRM-1 | yes* |
| **2081776918** | alpha-glucosidase (GH31) | | C08502 | 5658 | 62 | 6.5 | SRM-1 | yes* |
| **2081777232** | alpha-glucosidase (GH31) | | C02930 | 5348 | 55 | 6.0 | Bacteroidales | yes* |
| **2081806494** | beta-fructosidase (GH32) ^+SP^ | | C01699 | 4310 | 57 | 8.8 | Bacteroidales | yes* |
| **2081806495** | beta-fructosidase (GH32) ^+SP^ | | C01699 | 4310 | 57 | 8.8 | Bacteroidales | yes* |
| **2081819210** | beta-galactosidase (GH42) | | C51647 | 3055 | 56 | 7.5 | Bacteria | yes* |
| **2081799922** | alpha-amylase (GH57) | | C07268 | 10700 | 61 | 9.0 | SRM-1 | yes* |
| **2081799921** | alpha-glucan phosphorylases (GT35) | | C07268 | 10700 | 61 | 9.0 | SRM-1 | yes* |
| **2081799923** | glycosyltransferase (GT1) | | C07268 | 10700 | 61 | 9.0 | SRM-1 | yes* |
| **Polysaccharide Utilization Loci (PUL)** | | |  |  |  |  |  |  |
| **2081811883** | TonB dependent receptor (SusC) ^+SP^ | | C04697 | 8924 | 61 | 10.6 | SRM-1^†^ | yes* |
| **2081811884** | SusD ^+SP^ | | C04697 | 8924 | 61 | 10.6 | SRM-1^†^ | yes* |
| **2081811531** | TonB dependent receptor (SusC) ^+SP^ | | C04694 | 9509 | 54 | 11.3 | SRM-1 | yes* |
| **2081782876** | TonB dependent receptor (SusC) ^+SP^ | | C00077 | 8964 | 62 | 7.1 | Bacteroidales | yes* |
| **2081782875** | SusD ^+SP^ | | C00077 | 8964 | 62 | 7.1 | Bacteroidales | yes* |
| **2081782874** | TonB dependent receptor (SusC) ^+SP^ | | C00077 | 8964 | 62 | 7.1 | Bacteroidales | yes* |
| **2081823321** | TonB dependent receptor (SusC) ^+SP^ | | C00367 | 4416 | 61 | 11.0 | SRM-1 | yes* |
| **2081821152** | TonB dependent receptor (SusC) ^+SP^ | | C00318 | 4931 | 63 | 8.4 | SRM-1 | yes* |
| **2081795695** | TonB dependent receptor (SusC) ^+SP^ | | C08609 | 7532 | 58 | 7.9 | Bacteroidales | yes* |
| **2081795694** | SusD ^+SP^ | | C08609 | 7532 | 58 | 7.9 | Bacteroidales | yes* |
| **2081792856** | TonB dependent receptor (SusC) ^+SP^ | | C10567 | 7735 | 63 | 8.4 | SRM-1 | yes* |
| **2081778283** | SusD | | C00674 | 6185 | 61 | 6.3 | SRM-1 | yes* |
| **2081782765** | TonB dependent receptor (SusC) ^+SP^ | | C02146 | 9315 | 62 | 9.4 | Bacteroidales | yes* |
| **2081782766** | SusD ^+SP^ | | C02146 | 9315 | 62 | 9.4 | Bacteroidales | yes* |
| **2081818315** | TonB dependent receptor (SusC) | | C43585 | 3837 | 60 | 6.3 | SRM-1 |  |
| **2081818314** | SusD ^+SP^ | | C43585 | 3837 | 60 | 6.3 | SRM-1 |  |

* indicates best match; † indicates contig is linked to an uncultured *Bacteroidales* bacterium scaffold, based on PhyloPythiaS analysis; +SP indicates signal peptide detected. Taxonomic assignment was predicted using GC%, high coverage (greater than 6x) and PhyloPythiaS binning.
