# Supplementary material for: Metagenomics of the Svalbard Reindeer Rumen Microbiome Reveals Abundance of Polysaccharide Utilization Loci
Source: PLoS One. 2012 Jun 6;7(6):e38571. doi: 10.1371/journal.pone.0038571 (PMC3368933; doi:10.1371/journal.pone.0038571)
Supplement: Table S5 — A cellulase-linked PUL encoded within the as-yet uncultured Bacteroidales phylotype AC2a genome bin, reconstructed from the rumen metagenome [7] . * Gene ID’s are in the following format: NODE_ORF (see [7]). All data downloaded from ftp://ftp.jgi-psf.org/pub/rnd2/Cow_Rumen/† Sample ID and Substrate are as in Figure 3 and Table S6 from Hess et al. [7]. (DOC) [file pone.0038571.s005.doc]

**Table S5. A cellulase-linked PUL encoded within the as-yet uncultured Bacteroidales phylotype AC2a genome bin, reconstructed from the rumen metagenome [7].**

| **Gene ID*** | **PFAM ID** | **E-value** | **CAZy ID** | **Putative function** | **Sample ID (Acc. num.) †** | **Substrate†** |
| --- | --- | --- | --- | --- | --- | --- |
| **2527869_58650** | PF12833 | 5.2e-23 |  | Gene expression regulation |  |  |
| **2527869_58640** | PF06204  PF06165  PF06205 | 2.2e-23  1.4e-27  9.7e-25 | GH94 | Cellobiose phosphorylase |  |  |
| **2527869_58630** | PF00150 | 6.0e-52 | GH5 | Cellulase | TW-33 (ADX05734) | CMC |
| **2527869_58620** | PF00593 | 2.3e-13 |  | SusC-like |  |  |
| **2527869_58610** | PF12771 | 2.2e-29 |  | SusD-like |  |  |
| **2527869_58600** | PF01833 | 5.1e-06 |  | SusF-like |  |  |
| **2527869_58590** | PF13347 | 1.4e-90 |  | Sugar transport protein |  |  |
| **2527869_58580** | PF00759  PF02927 | 2.3e-73  3.0e-14 | GH9 | Cellulase  N-terminal ig-like domain | TW-64 (ADX05733) | Avicel, *Miscanthus* |

* Gene ID’s are in the following format: NODE_ORF (see [7]). All data downloaded from <ftp://ftp.jgi-psf.org/pub/rnd2/Cow_Rumen/> † Sample ID and Substrate are as in Figure 3 and Table S6 from Hess et al. [7].
