# Supplementary figures and images for: Metagenomics of the Svalbard Reindeer Rumen Microbiome Reveals Abundance of Polysaccharide Utilization Loci
Source: PLoS One. 2012 Jun 6;7(6):e38571. doi: 10.1371/journal.pone.0038571 (PMC3368933; doi:10.1371/journal.pone.0038571)

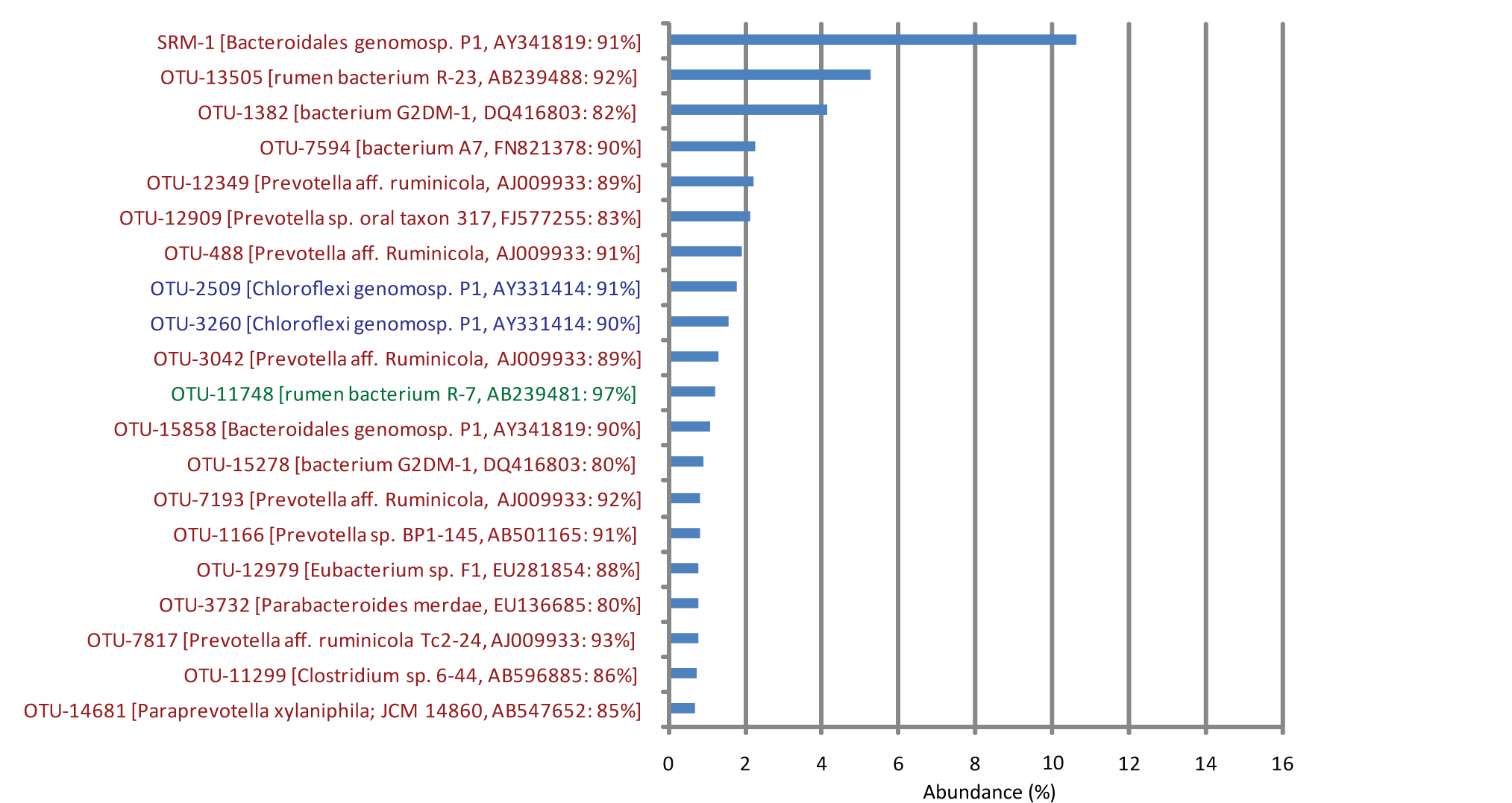

Supplement: Figure S1 — Relative abundance of the 20 most dominant bacterial taxa in the Svalbard reindeer rumen microbiome. Percentages are calculated against the total number of 16S rRNA gene sequences recovered. The closest cultured relative of each OTU and the sequence similarity % ID is indicated in parentheses. The lineage of each OTU is indicated by colour of text: Bacteroidetes maroon, Chloroflexi blue and Firmicutes green. (TIF) [file pone.0038571.s006.tif]
