# Supplementary material for: Correction: Metagenomics of the Svalbard Reindeer Rumen Microbiome Reveals Abundance of Polysaccharide Utilization Loci
Source: PLoS One. 2014 Jul 31;9(7):e104612. doi: 10.1371/journal.pone.0104612 (PMC4117644; doi:10.1371/journal.pone.0104612)
Supplement: File S1 — Corrected Table 2. (DOC) [file pone.0104612.s001.doc]

# Table 2. Summary of glycoside hydrolases and Sus proteins encoded within scaffolds reconstructed from sequenced fosmids selected from functional genomic screens.

| **Scaffold** | **GH2** | **GH3** | **GH5** | **GH9** | **GH10** | **GH16** | **GH20** | **GH23** | **GH26** | **GH28** | **GH29** | **GH31** | **GH33** | **GH35** | **GH43** | **GH51** | **GH53** | **GH55** | **GH74** | **GH76** | **GH78** | **GH94** | **GH109** | **GH117** | **PL1** | **PL22** | **CE1** | **CE7** | **CE11** | ***susC*** | ***susD*** | ***susE/F*** | **Binning** | **Sb.** |
| --- | --- | --- | --- | --- | --- | --- | --- | --- | --- | --- | --- | --- | --- | --- | --- | --- | --- | --- | --- | --- | --- | --- | --- | --- | --- | --- | --- | --- | --- | --- | --- | --- | --- | --- |
| **Sc00001** |  |  |  |  |  |  |  |  |  |  |  |  |  |  |  |  |  |  |  |  |  |  |  |  |  |  |  |  |  |  |  |  | SRM-1 | **C** |
| **Sc00002** | 1 |  | 1 |  |  |  |  | 1 | 2 |  |  |  |  |  |  |  |  |  |  |  |  |  |  |  |  |  |  | 2 | 1 | 1 | 1 | 2 | Prevotellaceae | **C** |
| **Sc00003** |  |  | 2 |  |  |  |  |  | 1 |  | 1 |  |  |  |  |  |  |  |  |  |  |  |  |  |  |  | 1 |  |  |  |  |  | Bacteroidaceae | **C** |
| **Sc00004** | 1 |  | 1 | 1 |  |  |  | 1 |  |  | 1 |  |  | 1 | 1 |  |  |  |  |  |  |  | 1 |  |  |  | 1 |  |  | 2 |  |  | Bacteroidaceae | **C** |
| **Sc00005** |  |  | 1 |  |  |  | 1 |  |  |  |  |  |  |  |  |  |  |  |  |  |  |  |  | 1 |  |  |  |  |  | 1 | 1 | 2 | Prevotellaceae | **C** |
| **Sc00006** |  |  | 2^BACON^ |  |  |  |  |  |  |  |  |  |  |  |  |  |  |  |  |  |  |  |  |  |  |  |  |  |  | 1 | 1 |  | Rikenellaceae | **C** |
| **Sc00007** | 1 | 3 |  |  | 1 |  |  |  |  |  |  |  |  |  | 1 |  |  |  |  |  |  | 1^CBM_X^ |  |  |  |  | 3 |  |  |  |  |  | Bacteroidaceae | **X** |
| **Sc00008** | 1 |  | 2^BACON^ |  |  |  |  |  |  | 1 |  |  | 1 |  |  | 1^CBM4^ |  | 1 | 1 |  |  |  |  |  | 2 |  |  |  |  | 1 | 1 | 2 | Prevotellaceae | **C** |
| **Sc00009** |  |  | 1 |  |  |  |  |  |  |  |  |  | 1 |  |  |  |  |  |  |  |  |  |  |  |  | 1 | 1 |  |  |  |  |  | Rikenellaceae | **C** |
| **Sc00010** | 1 |  | 3^BACON^ |  |  |  |  |  | 1 |  |  |  |  |  | 1 |  |  |  |  |  |  | 1^CBM_X^ |  |  |  |  | 1 | 1 |  | 2 | 2 | 1 | SRM-1 | **C** |
| **Sc00011** |  |  | 1 |  |  |  |  |  |  |  |  |  |  |  |  |  |  |  |  |  |  |  |  |  |  |  |  |  |  |  |  |  | Bacteroidaceae | **C** |
| **Sc00012** | 1 |  | 2 |  |  |  |  |  | 1^BACON^ |  |  |  |  |  | 1 |  |  |  |  |  |  |  |  |  |  |  | 1 |  |  |  |  |  | Bacteroidaceae | **C** |
| **Sc00013** |  |  |  |  |  |  |  |  |  |  |  |  |  |  |  | 1 |  |  |  |  |  |  |  |  |  |  |  |  |  |  |  |  | SRM-1 | **C** |
| **Sc00015** |  |  |  |  | 1^CBM6x3^ |  |  |  |  | 1 |  | 1 |  |  |  |  |  |  |  |  | 1 |  |  |  |  |  |  |  |  |  |  |  | Prevotellaceae | **X** |
| **Sc00016** |  |  | 1^BACON^ |  |  |  |  |  |  |  |  |  |  |  |  |  |  |  |  | 2 | 1 |  |  |  |  |  |  |  |  | 1 | 1 | 2 | Clostridiales | **C** |
| **Sc00017** |  |  | 1 |  |  |  |  |  |  |  |  |  |  |  |  |  |  |  |  |  |  |  |  |  |  |  |  |  |  |  |  |  | Ruminococcaceae | **C** |
| **Sc00018** |  |  |  |  | 1^CBM6^ |  |  |  |  |  |  |  |  |  | 2^CBM6^ | 1^CBM4^ |  |  |  |  |  |  |  |  |  |  |  |  |  |  |  |  | Prevotellaceae | **X** |
| **Sc00019** |  | 1 |  |  |  |  |  |  |  |  |  |  |  |  |  |  |  |  |  |  |  |  |  |  |  |  |  |  |  |  |  |  | SRM-1 | **C** |
| **Sc00020** |  |  | 2 ^BACON^ |  |  |  |  |  | 2 |  |  |  |  |  | 1 |  |  |  |  | 1 |  |  |  |  |  |  |  | 1 |  | 1 | 1 | 1 | Prevotellaceae | **C** |
| **Sc00021** |  |  |  |  |  |  |  |  |  |  |  |  |  |  |  |  | 1 |  |  |  |  |  |  |  |  |  |  |  |  | 1 | 1 | 1 | Bacteroidaceae | **C** |
| **Sc00022** |  |  | 1 |  |  | 1 |  |  |  |  |  |  |  |  |  |  |  |  |  |  |  |  |  |  |  |  |  |  |  |  |  |  | δ-proteobacteria | **C** |
| **Sc00023** |  |  | 1 |  |  |  |  |  |  |  | 1 | 1 |  |  |  |  |  |  |  |  |  |  |  |  |  |  |  |  |  |  |  |  | Clostridiales | **C** |
| **Sc00024** |  |  |  |  |  | 1 |  |  |  |  |  |  |  |  |  |  |  |  |  |  |  |  |  |  |  |  |  |  |  |  |  |  | Prevotellaceae | **C** |
| **Sc00025** |  | 1 | 2 ^BACON^ |  |  |  |  |  | 1 ^BACON^ |  |  |  |  |  | 1 |  |  |  |  |  |  |  |  |  |  |  |  |  |  | 1 | 1 | 2 | Bacteroidaceae | **C** |
| **Sc00026** |  |  | 2 ^BACON^ |  |  |  |  |  | 2 ^BACON^ |  |  |  |  |  |  |  |  |  |  |  |  |  |  |  |  |  |  |  |  | 1 | 1 | 2 | Bacteroidaceae | **C** |
| **Sc00028** |  |  | 2 ^BACON^ |  |  |  |  |  |  |  |  |  |  |  |  |  |  |  |  |  |  |  |  |  |  |  |  |  |  |  |  |  | Bacteroidaceae | **C** |
| **Sc00031** | 1 | 1 | 1 |  |  |  |  |  | 1 |  |  | 2 |  |  |  |  |  |  |  |  |  |  |  |  |  |  |  |  |  |  |  |  | Rikenellaceae | **C** |
